# Supplementary material for: Does bone mineral density improve the predictive accuracy of fracture risk assessment? A prospective cohort study in Northern Denmark
Source: BMJ Open. 2018 Apr 12;8(4):e018898. doi: 10.1136/bmjopen-2017-018898 (PMC5898344; doi:10.1136/bmjopen-2017-018898)
Supplement: Supplementary data [file bmjopen-2017-018898supp001.pdf]

Supplementary Information

Beta coefficients from each Cox regression model were used to create each fracture risk prediction model.

Once all 5 models were finalised, their beta coefficients were used to create 5 risk prediction models and calculate risk of fracture for each patient, using the following general equation:

$$\widehat{risk} = 1 - S_0(t)^{exp(\sum_{i=1}^{p} \beta_i X_i - \sum_{i=1}^{p} \beta_i \bar{X}_i)}$$

Where $\mathbf{S_0(t)}$ is the baseline survival rate at follow up time, $\mathbf{t}$(for this example, a follow up time of 10 years will be used); beta ($\boldsymbol{\beta_i}$) are the regression coefficients for each included risk factor in the model ($\mathbf{i}$); $\mathbf{X_i}$ is the observed data value for each risk factor; $\mathbf{\bar{X}_i}$ is the corresponding mean for each risk factor; and $\mathbf{p}$ is the total number of risk factors included in the model. Table A1 shows the formula for each risk prediction model explicitly.

Supplementary Table 1. Risk equations to calculate 4 year risk based on patient characteristics for each developed risk model.

**General Risk Equation to calculate 4 year risk**	$1 - 0.89^{exp(\sum_{i=1}^{p} \beta_i X_i - \sum_{i=1}^{p} \beta_i \bar{X}_i)}$
**Risk Model**	**Equation**
Model 1	where $\sum_{i=1}^{p} \beta_i X_i =$<br>0.0237745*age+-0.2826461*gender+-0.0225011*BMI+1.585278*previous fracture+<br>0.0762559*parental hip fracture+0.1138883*smoking status+0.0773898*glucocorticoid use+<br>0.3465287*alcohol consumption+0.0936966*rheumatoid arthritis+<br>-0.0069432*secondary osteoporosis+-0.4535108*(previous fracture*time)<br>where $\sum_{i=1}^{p} \beta_i \bar{X}_i =$<br>0.0237745*mean age+-0.2826461*mean gender+-0.0225011*mean BMI+<br>1.585278*mean previous fracture+0.0762559*mean parental hip fracture+<br>0.1138883*mean smoking status+0.0773898*mean glucocorticoid use+<br>0.3465287*mean alcohol consumption+0.0936966*mean rheumatoid arthritis+<br>-0.0069432*mean secondary osteoporosis+-0.4535108*mean (previous fracture*time)
Model 2	where $\sum_{i=1}^{p} \beta_i X_i =$<br>0.0186827*age+-0.228784*gender+-0.0113651*BMI+1.540559*previous fracture+<br>0.092011*parental hip fracture+0.0732564*smoking status+0.0508706*glucocorticoid use+<br>0.3649544*alcohol consumption+0.0854353*rheumatoid arthritis+ -0.0346885*secondary osteoporosis+0.5568944*osteoporosis+-0.4481145*(previous fracture*time)<br>where $\sum_{i=1}^{p} \beta_i \bar{X}_i =$<br>0.0186827*mean age+-0.228784*mean gender+-0.0113651*mean BMI+<br>1.540559*mean previous fracture+0.092011*mean parental hip fracture+<br>0.0732564*mean smoking status+0.0508706*mean glucocorticoid use+<br>0.3649544*mean alcohol consumption+0.0854353*mean rheumatoid arthritis+<br>-0.0346885*mean secondary osteoporosis+0.5568944*mean osteoporosis+<br>-0.4481145*mean (previous fracture*time)

[Table continues on the next page]

**Risk Model cont.**	**Equation**
Model 3	where $\sum_{i=1}^{p} \boldsymbol{\beta_i X_i} =$<br>0.0071931*age+-0.1615582*gender+0.0268478*BMI+1.39069*previous fracture+<br>0.1000272*parental hip fracture+0.0192416*smoking status+0.0374944*glucocorticoid use+<br>0.3774416*alcohol consumption+0.1097646*rheumatoid arthritis+ -0.0932063*secondary<br>osteoporosis+-0.5110986*t-score+-0.4404955*(previous fracture*time)<br>where $\sum_{i=1}^{p} \boldsymbol{\beta_i \bar{X}_i} =$<br>0.0071931*mean age+-0.1615582*mean gender+0.0268478*mean BMI+<br>1.39069*mean previous fracture+0.1000272*mean parental hip fracture+<br>0.0192416*mean smoking status+0.0374944*mean glucocorticoid use+<br>0.3774416*mean alcohol consumption+0.1097646*mean rheumatoid arthritis+<br>-0.0932063*mean secondary osteoporosis+-0.5110986*mean t-score+<br>-0.4404955*mean (previous fracture*time)

Supplementary Table 2. Crude fracture incidence rates for the derivation and validation datasets.

Risk Factor		Derivation			Validation		
		No of incident cases	Total Person years	Crude Incidence Rate per 10000 person years (95% CI)	No of incident cases	Total Person years	Crude Incidence Rate per 10000 person years (95% CI)
Age Category	40-49	17	1169.9	145.31 (90.33 to 233.74)	12	557.8	215.15 (122.19 to 378.84)
	50-59	70	2534.7	276.17 (218.49 to 349.07)	33	1311.0	251.71 (178.95 to 354.06)
	60-69	93	3062.9	303.63 (247.79 to 372.06)	42	1453.9	288.87 (213.48 to 390.88)
	70-79	83	1906.0	435.46 (351.17 to 539.98)	49	958.7	511.11 (386.29 to 676.26)
	80-89	52	652.7	796.75 (607.13 to 1045.59)	16	347.4	460.56 (282.15 to 751.77)
	90-99	1	26.6	376.51 (53.04 to 2672.85)	-	-	-
Osteoporotic - Hip	No	245	8475.9	289.05 (255.03 to 327.61)	123	4165.1	295.31 (247.48 to 352.40)
	Yes	71	876.8	809.73 (641.68 to 1021.78)	29	463.8	625.28 (434.52 to 899.78)
Osteoporotic - Spine	No	191	7025.8	271.86 (235.91 to 313.28)	111	3475.6	319.37 (265.16 to 384.67)
	Yes	119	2149.6	553.59 (462.55 to 662.55)	39	1089.1	358.08 (261.63 to 490.10)
Gender	Female	266	7417.5	358.61 (318.01 to 404.40)	129	3679.8	350.56 (295.00 to 416.59)
	Male	50	1935.3	258.36 (195.82 to 340.88)	23	949.0	242.36 (161.05 to 364.71)
Parental History Hip Fracture	No	220	6281.5	350.24 (306.88 to 399.71)	118	3108.7	379.58 (316.92 to 454.64)
	Yes	96	3071.3	312.57 (255.90 to 381.79)	34	1520.2	223.66 (159.81 to 313.02)

[Table continues on the next page]

Risk Factor cont.		Derivation No of incident cases	Derivation Total Person years	Derivation Crude Incidence Rate per 10000 person years (95% CI)	Validation No of incident cases	Validation Total Person years	Validation Crude Incidence Rate per 10000 person years (95% CI)
Current Smoker	No	240	7279.1	329.71 (290.53 to 374.18)	103	3513.1	293.19 (241.70 to 355.65)
	Yes	76	2073.7	366.5 (292.71 to 458.90)	49	1115.8	439.16 (331.91 to 581.07)
Alcohol Consumption more than 3 units per day	No	293	8875.9	330.11 (294.39 to 370.15)	140	4399.7	318.2 (269.63 to 375.53)
	Yes	23	476.9	482.33 (320.52 to 725.83)	12	229.2	523.66 (297.39 to 922.09)
Glucocorticoid Use (3 months)	No	279	8184.5	340.89 (303.15 to 383.33)	132	3993.1	330.57 (278.73 to 392.06)
	Yes	37	1168.3	316.7 (229.47 to 437.11)	20	635.8	314.57 (202.95 to 487.59)
Menopause	No	68	1962.8	346.44 (273.15 to 439.39)	29	928.9	312.19 (216.94 to 449.24)
	Yes	198	5454.7	362.99 (315.79 to 417.24)	100	2750.9	363.52 (298.82 to 442.23)
Premature Menopause (<45 years)	No	280	8175.4	342.49 (304.64 to 385.05)	127	4032.1	314.97 (264.69 to 374.81)
	Yes	36	1177.4	305.76 (220.56 to 423.89)	25	596.8	418.92 (283.07 to 619.97)
BMI - low (<18.5)	No	289	8876.1	325.59 (290.14 to 365.38)	141	4367.0	322.87 (273.75 to 380.82)
	Yes	11	187.0	588.16 (325.72 to 1062.04)	4	124.0	322.63 (121.09 to 859.62)
Rheumatoid Arthritis	No	289	8457.8	341.70 (304.49 to 383.45)	139	4135.1	336.15 (284.66 to 396.94)
	Yes	27	895.0	301.69 (206.90 to 439.93)	13	493.8	263.29 (152.88 to 453.43)

[Table continues on the next page]

		Derivation			Validation		
**Risk Factor cont.**		**No of incident cases**	**Total Person years**	**Crude Incidence Rate per 10000 person years (95% CI)**	**No of incident cases**	**Total Person years**	**Crude Incidence Rate per 10000 person years (95% CI)**
Secondary Osteoporosis	No	262	7846.5	333.9 (295.83 to 376.89)	122	3853.4	316.60 (265.12 to 378.07)
	Yes	54	1506.2	358.51 (274.58 to 468.10)	30	775.4	386.89 (270.51 to 553.34)
Previous Fracture	No	144	6832.0	210.77 (179.01 to 248.17)	63	3319.4	189.79 (148.26 to 242.95)
	Yes	172	2520.8	682.32 (587.61 to 792.31)	89	1309.4	679.69 (552.18 to 836.64)
Previous Fracture, detail	None	144	6832.0	210.77 (179.01 to 248.17)	63	3319.4	189.79 (148.26 to 242.95)
	1 fracture	105	1919.6	546.99 (451.76 to 662.29)	52	1049.8	495.36 (377.47 to 650.07)
	2-4 fractures	57	557.9	1021.62 (788.04 to 1324.45)	33	236.2	1397.28 (993.37 to 1965.44)
	5+ fractures	10	43.2	2311.27 (1243.59 to 4295.60)	4	23.5	1701.81 (638.72 to 4534.31)
**Total**		**316**	**9352.8**	**337.87 (302.60 to 377.25)**	**152**	**4628.8**	**328.38 (280.11 to 384.96)**

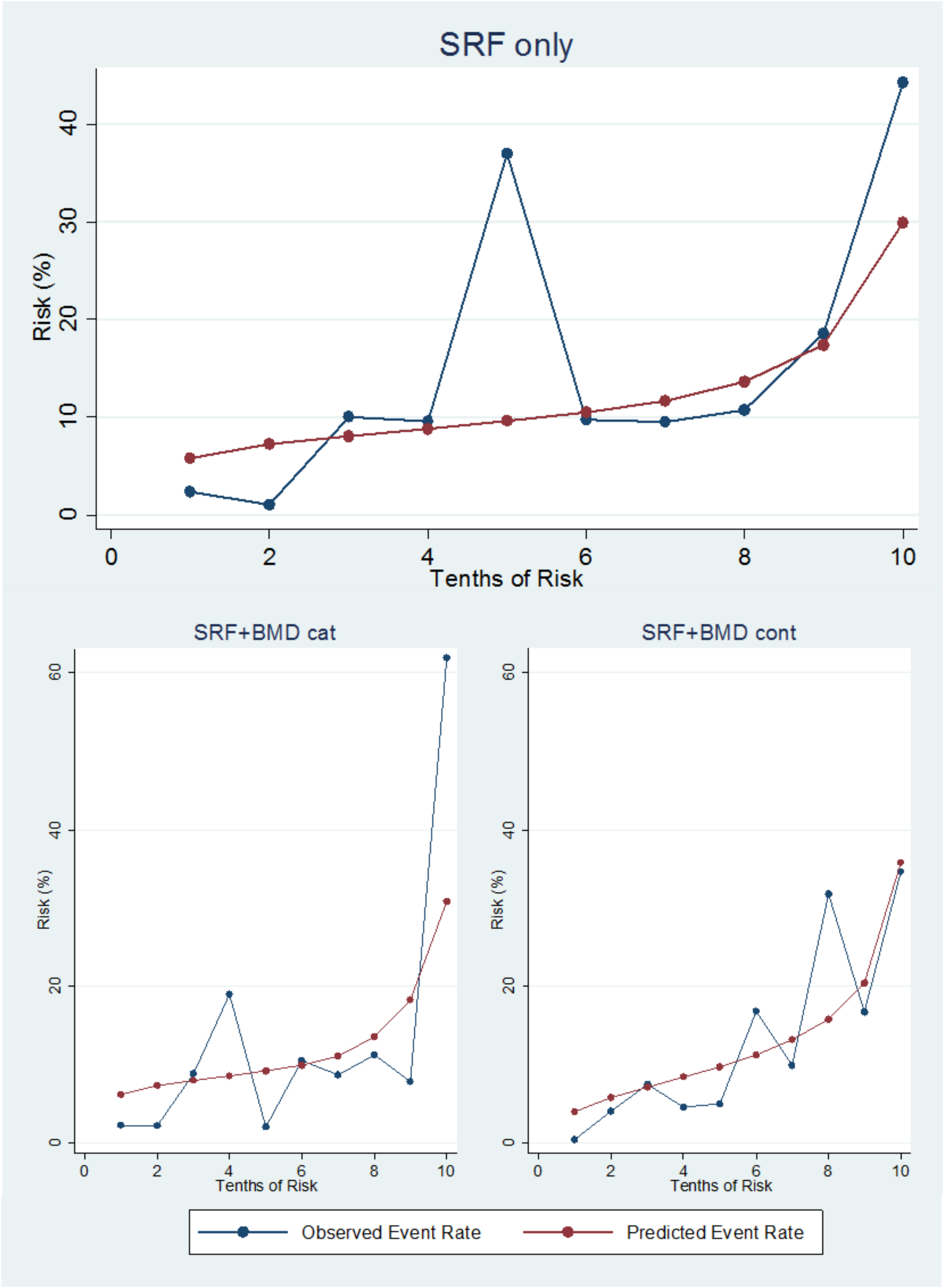

Supplementary Figure 1 Predicted and Observed risk by 10th of predicted risk for each risk prediction model in the derivation dataset.
