# Supplementary material for: Does bone mineral density improve the predictive accuracy of fracture risk assessment? A prospective cohort study in Northern Denmark
Source: BMJ Open. 2018 Apr 12;8(4):e018898. doi: 10.1136/bmjopen-2017-018898 (PMC5898344; doi:10.1136/bmjopen-2017-018898)
Supplement: Author's manuscript [file bmjopen-2017-018898.draft_revisions.pdf]

BMJ Open is committed to open peer review. As part of this commitment we make the peer review history of every article we publish publicly available.

When an article is published we post the peer reviewers' comments and the authors' responses online. We also post the versions of the paper that were used during peer review. These are the versions that the peer review comments apply to.

The versions of the paper that follow are the versions that were submitted during the peer review process. They are not the versions of record or the final published versions. They should not be cited or distributed as the published version of this manuscript.

BMJ Open is an open access journal and the full, final, typeset and author-corrected version of record of the manuscript is available on our site with no access controls, subscription charges or pay-per-view fees (http://bmjopen.bmj.com).

If you have any questions on BMJ Open's open peer review process please email info.bmjopen@bmj.com

BMJ Open

Does Bone Mineral Density Improve the Predictive Accuracy of Fracture Risk Assessment? A Prospective Cohort Study in Northern Denmark.

Journal:	*BMJ Open*
Manuscript ID	bmjopen-2017-018898
Article Type:	Research
Date Submitted by the Author:	28-Jul-2017
Complete List of Authors:	Dhiman, Paula; University of Nottingham, School of Medicine<br>Andersen, Stig; Aalborg Universitetshospital, Department of Clinical Medicine<br>Vestergaard, Peter; Aalborg Universitetshospital, Department of Clinical Medicine and Endocrinology<br>Masud, Tahir ; University of Nottingham School of Medicine<br>Qureshi, Nadeem; University of Nottingham, Division of Primary Care
Keywords:	risk prediction, osteoporosis, fracture, predictive accuracy

SCHOLARONE™
Manuscripts

For peer review only

For peer review only - http://bmjopen.bmj.com/site/about/guidelines.xhtml

Title

Does Bone Mineral Density Improve the Predictive Accuracy of Fracture Risk Assessment? A Prospective Cohort Study in Northern Denmark.

Authors list (names, affiliations, and position)

Paula Dhiman, Stig Andersen, Peter Vestergaard, Tahir Masud, Nadeem Qureshi

Division of Primary Care, University of Nottingham, Nottingham, NG7 2RD, UK

Paula Dhiman (Research Fellow)

Geriatric Medicine, Department of Clinical Medicine, Aalborg University Hospital, Hobrovej 18-22 Aalborg University Hospital, 9000 Aalborg, Denmark

Stig Andersen (Clinical Professor)

Department of Clinical Medicine and Endocrinology, Aalborg University Hospital Mølleparkvej 4, DK-9000 Aalborg, Denmark

Peter Vestergaard (Clinical Professor)

Geriatric Medicine, South Block, B Floor, Queens Medical Centre Campus, University of Nottingham, Nottingham NG7 2UH, UK

Tahir Masud (Honorary Professor)

Division of Primary Care, University of Nottingham, Nottingham, NG7 2RD, UK

Nadeem Qureshi (Clinical Professor)

Correspondence to: P Dhiman paula.dhiman@nottingham.ac.uk

Copyright

The Corresponding Author has the right to grant on behalf of all authors and does grant on behalf of all authors, a worldwide licence to the Publishers and its licensees in perpetuity, in all forms, formats and media (whether known now or created in the future), to i) publish, reproduce, distribute, display and store the Contribution, ii) translate the Contribution into other languages, create adaptations, reprints, include within collections and create summaries, extracts and/or, abstracts of the Contribution, iii) create any other derivative work(s) based on the Contribution, iv) to exploit all subsidiary rights in the Contribution, v) the inclusion of electronic links from the Contribution to third party material where-ever it may be located; and, vi) licence any third party to do any or all of the above.

Exclusive Licence Statement

The Corresponding Author has the right to grant on behalf of all authors and does grant on behalf of all authors, an exclusive licence (or non exclusive for government employees) on a worldwide basis to the BMJ Publishing Group Ltd to permit this article (if accepted) to be published in BMJ editions and any other BMJPGL products and sublicences such use and exploit all subsidiary rights, as set out in our licence.

For peer review only - http://bmjopen.bmj.com/site/about/guidelines.xhtml

For peer review only

Competing Interests

We have read and understood BMJ policy on declaration of interests and declare that we have no competing interests.

All authors have completed the ICMJE uniform disclosure form at www.icmje.org/coi_disclosure.pdf and declare: no support from any organisation for the submitted work; no financial relationships with any organisations that might have an interest in the submitted work in the previous three years; no other relationships or activities that could appear to have influenced the submitted work.

Transparency declaration

The lead author (Dr Dhiman) affirms that this manuscript is an honest, accurate, and transparent account of the study being reported; that no important aspects of the study have been omitted; and that any discrepancies from the study as planned (and, if relevant, registered) have been explained.

Ethical Approval

Ethics approval was given through the Region of North Jutland's from the Danish Data Protection Agency ("paraplyanmeldelse 2008-58-0028").

Funding

This study was funded through the National Institute for Health Research (NIHR), School for Primary Care Research (SPCR).

NIHR acknowledgement

This paper presents independent research funded by the National Institute for Health Research School for Primary Care Research (NIHR SPCR).

Disclaimer

The views expressed are those of the author(s) and not necessarily those of the NIHR, the NHS or the Department of Health.

Patient Involvement

Patients were not involved in the development of this research question.

Data Sharing Statement

Data sharing: Technical appendix and statistical code is available from the corresponding author at paula.dhiman@nottingham.ac.uk. Due to restrictions by the Danish Data Protection Agency, data can only be shared on an aggregated level and by special permission.

Contributorship

Contributors: PD wrote the statistical analysis plan, cleaned and analysed the data, and drafted and revised the paper. PV and SA provided the AURORA dataset for analysis and linked patients to the National Patient Registry of Denmark, they also reviewed and revised the draft paper. NQ and TM provided clinical expertise, and reviewed and revised the draft paper.

For peer review only

For peer review only - http://bmjopen.bmj.com/site/about/guidelines.xhtml

Abstract:

Objective: To evaluate the added predictive accuracy of bone mineral density (BMD) to fracture risk assessment.

Design: Prospective cohort study using data between 01/01/2010 and 31/12/2012.

Setting: North Denmark Osteoporosis Clinic of referred patients presenting with at least one fracture risk factor to the referring doctor.

Participants: Patients aged 40-90 years; had BMD T-score recorded at the hip; and not taking osteoporotic preventing drugs for more than 1-year prior to baseline.

Main outcome measures: Incident diagnoses of osteoporotic fractures (hip, spine, forearm, humerus, or pelvis) were identified using the National Patient Registry of Denmark during 01/01/2012-01/01/2014. Cox regression was used to model predictors based on Fracture Risk Assessment Tool (FRAX®), with and without, binary and continuous BMD. Change in Harrell's C-Index and Reclassification tables were used to describe the added statistical value of BMD.

Results: Adjusting for predictors included in FRAX®, osteoporotic patients (T-score≤-2.5) had 75% higher hazard of a fracture compared to patients with higher BMD (HR:1.75 (95% CI:1.28 to 2.38)). Forty-percent lower hazard was found per unit increase in continuous BMD T-score (HR:0.60 (95% CI:0.52 to 0.69)).

Accuracy improved, and Harrell's C-Index increased by 1.2% when adding continuous BMD (0.76 to 0.77). Reclassification tables showed continuous BMD shifted 463 patients into different risk categories; 274 of these were reclassified correctly (59%; 95% CI:55% to 64%). Adding binary BMD however showed little impact: Harrell's C-Index decreased by 0.6% and correctly reclassified 71 out of 109 patients (65%; 95% CI:55% to 74%).

Conclusions: Bone mineral density improves fracture risk prediction and should be incorporated in routine fracture risk assessment. Performance measures showed that using BMD in a continuous format is better than using a binary format. The added value of BMD to fracture risk prediction should be confirmed using routinely collected primary care data in other countries.

For peer review only

For peer review only - http://bmjopen.bmj.com/site/about/guidelines.xhtml

Article Summary

Strengths and Limitations:

- Addresses a research question recommended by The National Institute for Health and Care Excellence to investigate the added value of bone mineral density to fracture risk prediction.
- Investigates bone mineral density in both the commonly used, binary, and continuous format.
- Uses robustly collected data from Northern Denmark, with 3.2% missing data.
- As data is from a North Danish population, with at least one fracture risk factor, this limits generalisability of the results.
- Explores replacing current fracture risk factors, as well as adding to them, with bone mineral density.

For peer review only

For peer review only - http://bmjopen.bmj.com/site/about/guidelines.xhtml

Introduction

Osteoporosis causes over 8.9 million fractures worldwide, of which over 4.5 million occur in the USA and Europe, and account for 2.8 million disability adjusted life years (1). Further, 1.2 million disability adjusted life years are accounted for by hip fractures, which are projected to increase to 6 million by 2050 (2).

Given this burden, and treatment options for osteoporosis, identifying patients at risk of an osteoporotic fracture is high priority amongst health policymakers to reduce the risk of future fracture (3). Risk prediction tools have been developed to aid in the identification of patients at risk. For example, the Fracture Risk Assessment Tool (FRAX®) and QFracture® are commonly used to assess fracture risk in patients based on pre-defined risk factors.

Bone mineral density (BMD), a measurement used to aid diagnosis of osteoporosis, has also been identified as a fracture risk factor (4-7) . Unlike some other fracture risk factors, treatment options (e.g. bisphosphonate medication) are available that reduces the fracture risk markedly when treatment is initiated based on low BMD.

English National guidelines (The National Institute for Health and Care Excellence (NICE)) for fracture risk assessment recommend treatment of osteoporosis to prevent fractures but have not included BMD as a mandatory risk factor for fracture risk prediction tools to incorporate (8). This is partly due to the lack of robust evidence and limited generalisability of current research, which has particularly focused on evaluating BMD in postmenopausal women evaluating the added value of BMD to existing fracture risk factors (5-7).

The National Institute of Clinical Health and Excellence also recognise this gap in the evidence and have recommended research to assess the added value of BMD as a risk factor in fracture risk assessment (9).

The aim of this study is to assess the value of BMD measurement in addition to the standard fracture risk factors used in the FRAX® risk model using a robustly collected prospective cohort.

For peer review only

Methods

This paper has been written in accordance to the TRIPOD checklist.

Patient Involvement

Patients were not involved in the development of this research question and were not involved in the design of this study.

Study Design and Data Source

A prospective cohort study was conducted using patients from the Aalborg University Hospital Record for Osteoporosis Risk Assessment (AURORA) dataset; patients were followed up using the National Patient Registry of Denmark.

The AURORA dataset consists of patients attending the Osteoporosis Clinic at Aalborg University Hospital after a referral from their primary care physician. A referral was offered to patients with at least one risk factor for osteoporosis (low BMI, previous fracture, parental hip fracture, smoking status, alcohol consumption, glucocorticoid use, rheumatoid arthritis, and secondary osteoporosis) or if they were aged 80 years and above. Further detail of the data collection has been described elsewhere (10). The Danish National Patient Registry which collects inpatient and outpatient data from all Danish hospitals, was linked to the AURORA dataset through unique patient identifiers

Ethics approval was given through the Region of North Jutland's from the Danish Data Protection Agency ("paraplyanmeldelse 2008-58-0028").

Cohort selection

Data collection for AURORA began 1st January 2010 and was collected for 3 years (up to 31st December 2012). Patients were included if they were aged 40-90 years; had a BMD T-score at the hip; and were not taking any osteoporotic preventing drugs or any bone sparing drugs for more than one year prior to baseline.

Primary Outcome

The primary outcome measure was an incident osteoporotic fracture during follow up (01/01/2012 to 01/01/2014); defined as a diagnosis of a fracture at the hip, spine, forearm, humerus, and pelvis. Fractures at these sites resulting from traffic, work, and sports related accidents were excluded from the study. Relevant fractures were identified in the Danish National Patient Registry, using the International Statistical Classification of Diseases, 10th Version codes (ICD-10 codes), which was developed using recognised database methodology for each fracture (11).

Fracture risk factors

Fracture risk factors, used in the FRAX® risk prediction model, were extracted at baseline. They were: age; gender; height (m); weight (kg); previous fracture; parental history of hip fracture, current smoking status; current alcohol consumption; glucocorticoid use (currently exposed for 3+ months); rheumatoid arthritis; and secondary osteoporosis (includes type I diabetes; osteogenesis imperfecta in adults; untreated, long standing hyperthyroidism;

For peer review only - http://bmjopen.bmj.com/site/about/guidelines.xhtml

hypogonadism; premature menopause (<45 years); chronic malnutrition; malabsorption; and chronic liver disease).

Bone Mineral Density

DXA scans were performed by trained technicians using Hologic Discovery A (Bedford, MA, USA). A daily QC programme was in place and in vivo CV using repositioning of patients was <1%. Total hip BMD was used as region of interest. Bone mineral density was added to the fracture risk prediction model twice, firstly, as a continuously measured T-score value, and secondly, as a binary risk factor, dichotomised at/above T-score threshold for osteoporosis and below threshold, -2.5 in T-score (manufacturers' normal range using normal material from T Kelly et al (12)) based on World Health Organisation (WHO) classifications (13).

Statistical Analysis

A complete case analysis was performed on the data, 3.2% of data was missing. The AURORA dataset was split into two using recognised methodology (14); where a randomly was assigned to patients and based on a cut off two-thirds was used to derive the risk models, and the remaining third was used to validate them.

Model derivation

Five Cox proportional hazards models were developed for the primary outcome, using a complete case analysis:

Model 1. Standard fracture risk factors only (without BMD)
Model 2. Standard fracture risk factors (with binary BMD)
Model 3. Standard fracture risk factors (with continuous BMD)
Model 4. Data driven standard fracture risk factors (with binary BMD)
Model 5. Data driven standard fracture risk factors (with continuous BMD)

Graphical methods were used (log-log plots) to assess the proportional hazards assumption, and risk factors violating this assumption were added to the model as a time varying covariate. Data driven models were developed by removing risk factors which were not statistically significant, based on p-value<0.05, when adding continuous or binary BMD measurement.

Recognised methodology used in research studies was used to build the 5 risk prediction models (15, 16); the Kaplan Meier method was used to obtain 4-year fracture risk estimates for patients. Further detail on the conversion of the Cox proportional hazards models to risk prediction models has been provided in Supplementary Table 1.

Validation of Models

Four-year fracture risk was calculated from each model and the predictive performance of each risk prediction model was assessed by measures describing calibration, discrimination, and reclassification.

Calibration measures how well the predicted risk agrees with observed risk in the data. It plots the mean predicted and observed risk of fracture for each decile of predicted risk. The

For peer review only - http://bmjopen.bmj.com/site/about/guidelines.xhtml

observed risk of fracture was derived from the 4 year Kaplan-Meier estimate. Good calibration indicates the predicted risk is close to the observed risk of the outcome.

Discrimination measures how well the risk prediction model differentiates between patients who have or have not observed the event in the study. This was quantified by the area under the receiver operating characteristic (ROC) curve (AUC), given by Harrell's C-Index with higher values indicating better discrimination.

Reclassification tables (17) measures movement between risk categories when adding a new risk factor. Threshold for treatment at 4 years was set at a fracture risk level of 8.5%; to be comparable to the treatment threshold of 20% at 10 years. This was presented by the total percent of patients reclassified (incorrectly and correctly), and also the Net Reclassification Index (NRI) (18, 19). The NRI gives the net calculation of the changes in the right direction and a higher NRI indicates a better reclassifying model.

All analyses were carried out using Stata (version 12) (20).

For peer review only

For peer review only - http://bmjopen.bmj.com/site/about/guidelines.xhtml

Results

Characteristics of the data

The AURORA collected data on 7,912 patients; 1,795 patients were excluded comprising, 440 not aged between 40-90 years at baseline; 156 not having a recorded T-score value for the total hip at baseline; and 1,199 patients were taking anti-osteoporotic drug therapy for more than one year prior to baseline.

The study sample consisted of 6,117 patients; predominantly female (79.6%), and patients with a mean age of 62.9 (SD: 10.9) years. Two-thirds of this sample (n=4,093) was used for the derivation dataset and one-third (n=2,094) was used for the validation dataset. Table 1 presents the baseline characteristics of the study by derivation and validation dataset, and shows little difference between the datasets.

Patients in the derivation dataset observed 318 (7.8%) osteoporotic fractures during follow up. Of these, 316 fractures were eligible for the analysis (2 patients had a fractures on or prior to baseline and were excluded). Patients contributed 9352.8 person years of observation, giving a total incidence rate of 337.87 per 10,000 person years (95% CI:302.60 to 377.25).

Fractures during follow up were predominantly found in the forearm (27.0%) and hip (17.9%). Higher fracture incidence rates were found in patients classed as osteoporotic, based on their T-score at both the femoral neck (809.73 per 10,000 person years (95% CI:641.68 to 1021.78)) and spine (L1-L4) (553.59 per 10,000 person years (95% CI:462.55 to 662.55)) (Supplementary Table 2).

For peer review only - http://bmjopen.bmj.com/site/about/guidelines.xhtml

Table 1. Baseline characteristics of the derivation and validation datasets, including missing data.

Characteristic			Derivation (n=4,093)		Validation (n=2,024)	
			No	%	No	%
Gender	Female		3,266	79.79	1,602	79.15
	Male		827	20.21	422	20.85
Osteoporotic (Hip DXA)	No		3,683	89.98	1,820	89.92
	Yes		410	10.02	204	10.08
Osteoporotic Status (based on UK guidelines)	Normal		1,886	46.08	927	45.8
	Osteopenic		1,797	43.90	893	44.12
	Osteoporotic		410	10.02	204	10.08
Previous Fracture	No		2,935	71.71	1,423	70.31
	Yes		1,158	28.29	601	29.69
No of Previous Fractures	None		2,935	71.71	1,423	70.31
	1 fracture		862	21.06	467	23.07
	2-4 fractures		270	6.60	122	6.03
	5+ fractures		26	0.64	12	0.59
Parental History of Hip Fracture	No		2,755	67.31	1,359	67.14
	Yes		1,338	32.69	665	32.86
Current Smoking Status	other (non/ex)		3,182	77.74	1,529	75.54
	smoker		911	22.26	495	24.46
Alcohol Consumption	<3		3,875	94.67	1,923	95.01
	>3 units		218	5.33	101	4.99
Glucocorticoid Use	No		3,577	87.39	1,741	86.02
	Yes		516	12.61	283	13.98
Rheumatoid Arthritis	No		3,686	90.06	1,801	88.98
	Yes		407	9.94	223	11.02
Other Bone Affecting Disease	No		2,382	58.20	1,139	56.27
	Yes		1,711	41.80	885	43.73
Secondary Osteoporosis	No		3,438	84.00	1,689	83.45
	Yes		655	16.00	335	16.55
	By disease					
	Type 1 diabetes	No	4,010	97.97	1,981	97.88
		Yes	83	2.03	43	2.12
	Osteogenesis	No	4,093	100	2,024	100
		Yes	0	0	0	0
	Hypothyroidism	No	4,089	99.9	2,023	99.95
		Yes	4	0.1	1	0.05
	Malnutrition	No	4,090	99.93	2,023	99.95
		Yes	3	0.07	1	0.05
	Chronic Liver Disease	No	4,006	97.87	1,979	97.78
		Yes	87	2.13	45	2.22
	Menopause (Females only)**	No	853	20.84	405	20.01
		Yes	2,413	58.95	1,197	59.14

For peer review only - http://bmjopen.bmj.com/site/about/guidelines.xhtml

	Premature Menopause (<45years)***	No	1,904	46.52	941	46.49
		Yes	509	12.44	256	12.65
			**Mean**	**SD**	**Mean**	**SD**
Age (years)			62.91	10.92	62.99	10.96
Weight (kg)			72.12	15.47	72.23	15.86
	Missing		47	1.15	12	0.59
Height (m)			1.65	0.08	1.65	0.08
	Missing		131	3.2	61	3.01
BMI			26.39	5.04	26.39	5.13
	Missing		135	3.3	63	3.11
Hip DXA T-score			-1.13	1.09	-1.16	1.08

*out of patients with a fracture

**proportion out of respective number of females

***proportion out of respective number of females with menopause

Model development

The unadjusted analysis showed statistically significant association between BMD (continuous and binary) and osteoporotic fracture (p<0.001). Significant associations with fracture were also found with age (p<0.001), previous fracture (p<0.001), BMI (p=0.03), and gender (p=0.05). Further, a time-varying effect was found in patients with a previous fracture; hazard of a subsequent fracture was highest in the first year during follow up and decreased per year of follow up (p<0.001).

The adjusted analysis is presented in Table 2. Model 1 showed that of the standard risk factors, age and previous fracture were significantly associated with fracture; hazard of fracture increased by 2% per year increase in age (HR=1.02; 95% CI: 1.01 to 1.04); and increased almost 5 fold in patients with a previous fracture (HR=4.88; 95% CI: 3.37 to 7.08).

Adding binary and continuous BMD to standard risk factors (Model 2) led to 75% increased hazard of fracture (HR=1.75; 95% CI: 1.28 to 2.38), whilst adding continuous BMD T-score (Model 3) led to a 40% lower hazard per SD improvement in BMD T-score (HR=0.60; 95% CI: 0.52 to 0.69).

Insignificant risk factors were also removed (Model 4 and 5). Removing secondary osteoporosis when adding binary BMD (Model 4), and removing secondary osteoporosis, current smoker, and BMI, when adding continuous BMD gave similar results but simplified the model.

For peer review only - http://bmjopen.bmj.com/site/about/guidelines.xhtml

Table 2. Multivariate analysis for osteoporotic fracture in the derivation cohort. Data are adjusted hazard ratios and 95% confidence intervals.

Risk Factor		Adjusted Hazard Ratio (95% CI)				
		Model 1: Standard Risk Factors only	Model 2: Standard Risk Factors + BMD (categorical)	Model 3: Standard Risk Factors + BMD (continuous)	Model 4: Data Driven Standard Risk Factors + BMD (categorical)	Model 5: Data Driven Standard Risk Factors + BMD (continuous)
Age (years)		1.02 (1.01 to 1.04)	1.02 (1.01 to 1.03)	1.01 (1.00 to 1.02)	1.02 (1.01 to 1.03)	1.01 (1.00 to 1.02)
Gender	Female	Ref	Ref	Ref	Ref	Ref
	Male	0.75 (0.54 to 1.04)	0.80 (0.57 to 1.10)	0.85 (0.61 to 1.18)	0.80 (0.58 to 1.11)	0.86 (0.62 to 1.20)
BMI		0.98 (0.95 to 1.00)	0.99 (0.96 to 1.01)	1.03 (1.00 to 1.05)	0.99 (0.96 to 1.01)	-
Previous Fracture	No	Ref	Ref	Ref	Ref	Ref
	Yes	4.88 (3.37 to 7.08)	4.67 (3.21 to 6.78)	4.02 (2.76 to 5.84)	4.66 (3.21 to 6.77)	4.09 (2.82 to 5.95)
Parental History of Hip Fracture	No	Ref	Ref	Ref	Ref	Ref
	Yes	1.08 (0.83 to 1.40)	1.10 (0.85 to 1.42)	1.11 (0.85 to 1.43)	1.10 (0.85 to 1.42)	1.10 (0.85 to 1.42)
Current Smoker	No	Ref	Ref	Ref	Ref	Ref
	Yes	1.12 (0.85 to 1.47)	1.08 (0.82 to 1.42)	1.02 (0.77 to 1.34)	1.07 (0.82 to 1.41)	-
Alcohol Consumption (>3 units/day)	No	Ref	Ref	Ref	Ref	Ref
	Yes	1.41 (0.90 to 2.21)	1.44 (0.92 to 2.25)	1.04 (0.72 to 1.49)	1.44 (0.92 to 2.25)	1.43 (0.92 to 2.22)
Glucocorticoid Use	No	Ref	Ref	Ref	Ref	Ref
	Yes	1.08 (0.75 to 1.55)	1.05 (0.73 to 1.51)	1.46 (0.93 to 2.28)	1.05 (0.73 to 1.51)	1.04 (0.73 to 1.49)
Rheumatoid Arthritis	No	Ref	Ref	Ref	Ref	Ref
	Yes	1.10 (0.73 to 1.65)	1.09 (0.72 to 1.64)	1.12 (0.74 to 1.68)	1.09 (0.73 to 1.64)	1.12 (0.75 to 1.69)
Secondary Osteoporosis	No	Ref	Ref	Ref	Ref	Ref
	Yes	0.99 (0.73 to 1.35)	0.97 (0.71 to 1.32)	0.91 (0.67 to 1.24)	-	-
Osteoporotic	No	Ref	Ref	Ref	Ref	Ref
	Yes	-	1.75 (1.28 to 2.38)	-	1.74 (1.28 to 2.38)	-
Hip DXA T-score (SD)		-	-	0.60 (0.52 to 0.69)	-	0.64 (0.57 to 0.72)
Previous Fracture (TVC*)		0.64 (0.49 to 0.83)	0.64 (0.49 to 0.83)	0.64 (0.50 to 0.84)	0.64 (0.49 to 0.83)	0.64 (0.50 to 0.84)

*TVC = time varying covariate

For peer review only - http://bmjopen.bmj.com/site/about/guidelines.xhtml

Model Validation

The 4-year predicted risk of fracture was calculated for all patients in the validation dataset; this was compared to the observed fracture outcome within the 4 year follow up.

Calibration and Discrimination

Calibration improved when adding BMD measurement; particularly when including continuous BMD T-score measurement (Model 3; Supplementary Figure 1).

The largest change in discrimination was found when adding continuous BMD measurement to standard risk factors; Harrell's C-Index increased by 1.15% (Table 3). However, binary BMD measurement, as a measure for osteoporotic patients, was found to reduce Harrell's C-Index by -0.62%.

Table 3. Harrell's C-Index for Model 1, 2, 3, 4, and 5.

Model	Harrell's C-Index	Change in Harrell's C-Index (% change)*
**Model 1**: Standard fracture risk factors only (without BMD)	0.7640 (0.7181 to 0.8099)	-
**Model 2**: Standard fracture risk factors only (with binary BMD)	0.7592 (0.7124 to 0.8061)	-0.0048 (-0.62%)
**Model 3**: Standard fracture risk factors only (with continuous BMD)	0.7728 (0.7317 to 0.8139)	0.0088 (1.15%)
**Model 4:** Data driven standard fracture risk factors (with binary BMD)	0.7587 (0.7118 to 0.8056)	-0.0053 (-0.69%)
**Model 5**:Data driven standard fracture risk factors (with continuous BMD)	0.7707 (0.7294 to 0.8120)	0.0067 (0.88%)

*All change is measures against Model 1.

Reclassification

Reclassification tables showed risk models with continuous BMD measurement improved classification of patients into their correct risk categories. This was not found when adding binary BMD. Table 4 presents the reclassification table for Model 1 (standard fracture risk factors only) and Model 3 (standard risk factors with continuous BMD), using the 8.5% pre-specified risk threshold.

For peer review only - http://bmjopen.bmj.com/site/about/guidelines.xhtml

Table 4. Risk Reclassification Table comparing Model 1 (standard fracture risk factors alone) to Model 3 (standard fracture risk factors with continuous BMD measurement), using a clinical 8.5% risk cut off.

			Model 3: SRF with continuous BMD			
			<8.5%	≥8.5%	Total	Total No.(%) Reclassified
Model 1: SRF without BMD	<8.5%	No	391	227	618	227 (36.73%)
		%	63.27%	36.73%		
		No. Events	5	9		
		No. Non events	386	218		
		Observed Event Rate	1.28%	3.96%		
	≥8.5%	No	302	1,040	1,342	302 (22.5%)
		%	22.50%	77.50%		
		No. Events	10	121		
		No. Non events	292	919		
		Observed Event Rate	3.31%	11.63%		
	Total		693	1,267	1,960	529 (26.99%)

Of the 1,960 patients in the validation dataset, 27% (n=529) were reclassified into a different risk category when including continuous BMD into fracture risk prediction. Two percent (9/529) were found to be reclassified correctly into a higher risk group and 55% (292/529) were reclassified correctly into a lower risk group; indicating 22% (292/1342) of patients at high risk in Model 1, not accounting for BMD measurement, were no longer at high risk. The net reclassification improvement when adding continuous BMD to standard risk factors, was 0.03, similar results were found when comparing Model 1 with the data driven models (Table 5).

For peer review only

Table 5. Summary of Net Reclassification Index (NRI) and Integrated Discrimination Index (IDI) for all comparisons between developed fracture risk prediction models.

Comparison	Event NRI	Non-Event NRI	Overall NRI
Model 1 vs. Model 2	-3.45%	2.09%	-0.01
Model 1 vs. Model 3	-0.69%	4.08%	0.03
Model 1 vs. Model 4	-4.14%	1.98%	-0.02
Model 1 vs. Model 5	-0.69%	4.74%	0.04

For peer review only - http://bmjopen.bmj.com/site/about/guidelines.xhtml

Discussion

Summary of Findings

Bone mineral density improved fracture risk prediction. This finding was consistent throughout the analysis; both the unadjusted and adjusted analyses. However, the format of BMD measurement in the fracture risk prediction model affected the results. Calibration, discrimination, and reclassification all improved when adding continuous BMD measurement to standard risk factors. This was not found when adding BMD in a binary format.

Adding BMD to fracture risk prediction model negated the effect of fracture with secondary osteoporosis, current smoking status, and BMI. Removing these risk factors had minimal impact on the model performance.

Strengths and Limitations

Answering Evidence gap

To our knowledge, this is the first study to investigate the added value of BMD in a binary and continuous format, to standard fracture risk factors. It directly informs the NICE research recommendation to assess the added value of BMD to routine fracture risk assessment in primary care (21). It further highlights that the more commonly used, binary format of BMD resulted in a loss of predictability in fracture risk prediction; based on comparable measures for discrimination and reclassification

Robustness of Data

The prospective cohort was well populated with key standard risk factors recorded: BMI, smoking status and alcohol consumption, and personal and parental fracture history. Other than 3.2% of missing data for BMI, in 6,117 patients, complete data was collected for all risk factors (including BMD T-score recorded at the total hip). Further, the cohort was linked to a national robust electronic health records. This Danish National Patient Registry allowed for outcome fracture to be identified and also provided data on the mechanism for the fracture; this helped more accurately phenotype osteoporotic fractures.

Generalisability

The generalisability is affected in two ways. Firstly, the findings are based on a Danish cohort. Secondly, AURORA data was collected from patients who presented to their doctor with at least one fracture risk factor and were referred to the osteoporosis clinic; this led to a biased study sample with a higher risk of a fracture and increased age. This could overestimate fracture risk amongst patients in a primary care setting.

Methodology

As well as assessing the added value of BMD to standard, we have also explored the option to replace existing fracture risk factors with the BMD measurement; this has rarely been explored in the literature but should be considered in future analyses. (22, 23).

Due to the increased age of the sample, death becomes a competing risk. However, information on death was not collected and could not be retrieved. This limited the analysis of the data as competing risks could not be accounted for which may again lead to an

For peer review only - http://bmjopen.bmj.com/site/about/guidelines.xhtml

overestimation of fracture risk (24). However, as an independent study primarily assessing the added value of BMD through deriving and validating the fracture risk prediction models, this bias would be present in both analyses to compare derived risk models with and without BMD measurement.

Internal validation was performed to validate the derived risk prediction models. This may lead to over optimistic results of the performance of the risk models (14). To account for this limitation, a commonly practised method which randomly assigns patients to the derivation and validation datasets was used; further, a similar 1:3 ratio was also used to split the data (25-27).

The study had a 4 year follow up which is shorter than other recognised risk models. To account for this, we adapted the 20% clinical risk threshold for 10 year fracture estimates to 8.5% for 4 year fracture estimates (28, 29).

Traditional methodology assessing the added value to risk factors to existing risk prediction models are criticised to be insensitive to change, to lack interpretability (30-33); and do not account for cost implications. Reclassification analysis was used to provide more clinically interpretable results.

Clinical implications

The most notable clinical implication is the more routine use of BMD measurement for fracture risk assessment. Further, evidence suggests continuous BMD adds better predictability compared to the binary format.

Future Research

Further research is recommended to evaluate the added value of BMD to fracture risk prediction; in particular using primary care routinely collected data. However, a brief interrogation into the Clinical Practice Research Datalink, a routinely collected UK primary care database, showed poor availability of BMD measurement in patient records, and thus, strong limitations to potential analyses. Less than 1% of patients had BMD recorded from a sample of 60,658 patients aged 40-90; not on any osteoporotic treatment; and with complete data for age, gender, BMI, smoking status, and alcohol consumption. Thus, prior to UK analysis, BMD recording in primary care databases needs to improve.

In addition, further research is recommended to develop current methodology used to assess the added value of BMD to provide more clinically relevant results, such as cost implications; and to allow for better comparability between new risk factors with respect to their added value, thus improving decision making.

Conclusion

BMD improves fracture risk assessment, and may replace some standard fracture risk factors. However, improved performance of the fracture risk assessment was only demonstrated when using continuous BMD measurement for osteoporosis. Research is recommended to explore replacing existing risk factors as well as adding new risk factors to established models; develop more clinically relevant methodology to assess the added value of a new risk factor.

For peer review only - http://bmjopen.bmj.com/site/about/guidelines.xhtml

References

1. Kanis JA on behalf of the World Health Organization Scientific Group (2007). Assessment of osteoporosis at the primary health-care level. Technical Report. World Health Organization Collaborating Centre for Metabolic Bone Diseases, University of Sheffield, UK; 2007.
2. WHO scientific group. Prevention and management of osteoporosis: report of a WHO scientific group. WHO Technical Report Series. 2003.
3. Barry P, Aspray T, Briers K, Collins GS, Compston J, Dockery F, et al. Osteoporosis: assessing the risk of fragility fracture. 2012. Contract No.: CG146.
4. Kanis JA, Borgstrom F, Laet CD, Johansson H, Johnell O, Jonsson B, et al. Assessment of fracture risk. Osteoporosis International. 2005;16(6):581-9.
5. Report of a WHO Study Group. Assessment of fracture risk and its application to screening for postmenopausal osteoporosis. 1994.
6. McCloskey E, Johansson H, Oden A, Kanis JA. Fracture risk assessment. Clinical biochemistry. 2012;45(12):887-93.
7. Briot K, Paternotte S, Kolta S, Eastell R, Felsenberg D, Reid DM, et al. FRAX: Prediction of Major Osteoporotic Fractures in Women from the General Population: The OPUS Study. PLoS ONE. 2013;8(12):e83436.
8. National Institute for Health and Care Excellence. Osteoporosis: assessing the risk of fragility fracture. Short clinical guideline - evidence and recommedation. London: National Clinical Guidance Centre. 2012.
9. National Institute for Health and Care Excellence. Osteoporosis: assessing the risk of fragility fracture. NICE short clinical guideline CG146.; 2012.
10. Andersen S, Laurberg P. Age discrimination in osteoporosis screening-data from the Aalborg University Hospital Record for Osteoporosis Risk Assessment (AURORA). Maturitas. 2014;77(4):330-5.
11. Dave S, Petersen I. Creating medical and drug code lists to identify cases in primary care databases. Pharmacoepidemiol Drug Saf. 2009;18(8):704-7.
12. Kelly TL. Bone mineral density reference databases for American men and women. Journal of Bone and Mineral Research. 1990;5(Suppl 2).
13. Kanis JA, Melton LJr, Christianson C, Johnston CC, Khaltaev N. The diagnosis of osteoporosis. Journal of Bone and Mineral Research. 1994;9(8):1137-41.
14. Moons KGM, Kengne AP, Woodward M, Royston P, Vergouwe Y, Altman DG, et al. Risk prediction models: I. Development, internal validation, and assessing the incremental value of a new (bio)marker. Heart. 2012.
15. Hippisley-Cox J, Coupland C. Derivation and validation of updated QFracture algorithm to predict risk of osteoporotic fracture in primary care in the United Kingdom: prospective open cohort study. Bmj. 2012;344:e3427.
16. Hippisley-Cox J, Coupland C, Vinogradova Y, Robson J, Minhas R, Sheikh A, et al. Predicting cardiovascular risk in England and Wales: prospective derivation and validation of QRISK2. British Medical Journal. 2008;336:1475-82.
17. Cook NR. Use and misuse of the receiver operating characteristic curve in risk prediction. Circulation. 2007;115(7):928-35.
18. Cook NR. Comments on 'Evaluating the added predictive ability of a new marker: From area under the ROC curve to reclassification and beyond' by M. J. Pencina et al., Statistics in Medicine. Statistics in Medicine. 2008;27(2):191-5.
19. Pencina MJ, D'Agostino RB, Sr., Steyerberg EW. Extensions of net reclassification improvement calculations to measure usefulness of new biomarkers. Statistics in Medicine. 2011;30(1):11-21.

For peer review only - http://bmjopen.bmj.com/site/about/guidelines.xhtml

20. StataCorp. Stata Statistical Software: Release 12. College Station, TX: StataCorp LP; 2011.
21. Barry P, Aspray T, Briers K, Collins GS, Compston J, Dockery F, et al. Osteoporosis: assessing the risk of fragility fracture (NICE Clinical Guidance 146). National Institute for Health and Care Excellence; 2012. Contract No.: CG146.
22. Ridker PM, Paynter NP, Rifai N, Gaziano JM, Cook NR. C-Reactive Protein and Parental History Improve Global Cardiovascular Risk Prediction The Reynolds Risk Score for Men. Circulation. 2008;118(22):2243-4.
23. Woodward M, Brindle P, Tunstall-Pedoe H, Estimation SGR. Adding social deprivation and family history to cardiovascular risk assessment: the ASSIGN score from the Scottish Heart Health Extended Cohort (SHHEC). Heart. 2007;93(2):172-6.
24. Satagopan JM, Ben-Porat L, Berwick M, Robson M, Kutler D, Auerbach AD. A note on competing risks in survival data analysis. Br J Cancer. 2004;91(7):1229-35.
25. Hippisley-Cox J, Coupland C, Vinogradova Y, Robson J, May M, Brindle P. Derivation and validation of QRISK, a new cardiovascular disease risk score for the United Kingdom: prospective open cohort study. British Medical Journal. 2007;335(7611):136-41.
26. Ridker PM, Buring JE, Rifai N, Cook NR. Development and validation of improved algorithms for the assessment of global cardiovascular risk in women: the Reynolds Risk Score.[Erratum appears in JAMA. 2007 Apr 4;297(13):1433]. Jama. 2007;297(6):611-9.
27. Hippisley-Cox J, Coupland C, Robson J, Brindle P. Derivation, validation, and evaluation of a new QRISK model to estimate lifetime risk of cardiovascular disease: cohort study using QResearch database. British Medical Journal. 2010;341.
28. National Osteoporosis Foundation. Clinician's Guide to Prevention and Treatment of Osteoporosis. Washington, DC; 2013.
29. Thomsen K. Odense Fallers and Osteoporosis Study: Assessment of osteoporosis among older women presenting with falls. Denmark: Odense University Hospital; 2014.
30. McGeechan K, Macaskill P, Irwig L, Liew G, Wong TY. Assessing New Biomarkers and Predictive Models for Use in Clinical Practice A Clinician's Guide. Archives of Internal Medicine. 2008;168(21):2304-10.
31. Steyerberg EW, Pencina MJ, Lingsma HF, Kattan MW, Vickers AJ, Van Calster B. Assessing the incremental value of diagnostic and prognostic markers: a review and illustration. European Journal of Clinical Investigation. 2012;42(2):216-28.
32. Janes H, Pepe MS, Gu W. Assessing the Value of Risk Predictions by Using Risk Stratification Tables. Annals of Internal Medicine. 2008;149(10):751-W162.
33. Cook NR. Use and misuse of the receiver operating characteristic curve in risk prediction - Response. Circulation. 2007;116(6):E134-E.

For peer review only

For peer review only - http://bmjopen.bmj.com/site/about/guidelines.xhtml

Supplementary Information

Beta coefficients from each Cox regression model were used to create each fracture risk prediction model.

Once all 5 models were finalised, their beta coefficients were used to create 5 risk prediction models and calculate risk of fracture for each patient, using the following general equation:

$$\widehat{risk} = 1 - S_0(t)^{exp(\sum_{i=1}^{p}\beta_i X_i - \sum_{i=1}^{p}\beta_i \bar{X}_i)}$$

Where $\mathbf{S_0(t)}$ is the baseline survival rate at follow up time, $\mathbf{t}$(for this example, a follow up time of 10 years will be used); beta ($\boldsymbol{\beta_i}$) are the regression coefficients for each included risk factor in the model (**i**); $\mathbf{X_i}$ is the observed data value for each risk factor; $\mathbf{\bar{X}_i}$ is the corresponding mean for each risk factor; and **p** is the total number of risk factors included in the model. Table A1 shows the formula for each risk prediction model explicitly.

For peer review only

Supplementary Table 1. Risk equations to calculate 4 year risk based on patient characteristics for each developed risk model.

General Risk Equation to calculate 4 year risk	$1 - 0.89^{exp(\sum_{i=1}^{p} \beta_i X_i - \sum_{i=1}^{p} \beta_i \bar{X}_i)}$
**Risk Model**	**Equation**
Model 1	where $\sum_{i=1}^{p} \beta_i X_i =$<br>0.0237745*age+-0.2826461*gender+-0.0225011*BMI+1.585278*previous fracture+<br>0.0762559*parental hip fracture+0.1138883*smoking status+0.0773898*glucocorticoid use+<br>0.3465287*alcohol consumption+0.0936966*rheumatoid arthritis+<br>-0.0069432*secondary osteoporosis+-0.4535108*(previous fracture*time)<br>where $\sum_{i=1}^{p} \beta_i \bar{X}_i =$<br>0.0237745*mean age+-0.2826461*mean gender+-0.0225011*mean BMI+<br>1.585278*mean previous fracture+0.0762559*mean parental hip fracture+<br>0.1138883*mean smoking status+0.0773898*mean glucocorticoid use+<br>0.3465287*mean alcohol consumption+0.0936966*mean rheumatoid arthritis+<br>-0.0069432*mean secondary osteoporosis+-0.4535108*mean (previous fracture*time)
Model 2	where $\sum_{i=1}^{p} \beta_i X_i =$<br>0.0186827*age+-0.228784*gender+-0.0113651*BMI+1.540559*previous fracture+<br>0.092011*parental hip fracture+0.0732564*smoking status+0.0508706*glucocorticoid use+<br>0.3649544*alcohol consumption+0.0854353*rheumatoid arthritis+ -0.0346885*secondary<br>osteoporosis+0.5568944*osteoporosis+-0.4481145*(previous fracture*time)<br>where $\sum_{i=1}^{p} \beta_i \bar{X}_i =$<br>0.0186827*mean age+-0.228784*mean gender+-0.0113651*mean BMI+<br>1.540559*mean previous fracture+0.092011*mean parental hip fracture+<br>0.0732564*mean smoking status+0.0508706*mean glucocorticoid use+<br>0.3649544*mean alcohol consumption+0.0854353*mean rheumatoid arthritis+<br>-0.0346885*mean secondary osteoporosis+0.5568944*mean osteoporosis+<br>-0.4481145*mean (previous fracture*time)

[Table continues on the next page]

For peer review only

Risk Model cont.	Equation
Model 3	where $\sum_{i=1}^{p} \boldsymbol{\beta}_i \boldsymbol{X}_i =$<br>0.0071931*age+-0.1615582*gender+0.0268478*BMI+1.39069*previous fracture+<br>0.1000272*parental hip fracture+0.0192416*smoking status+0.0374944*glucocorticoid use+<br>0.3774416*alcohol consumption+0.1097646*rheumatoid arthritis+ -0.0932063*secondary<br>osteoporosis+-0.5110986*t-score+-0.4404955*(previous fracture*time)<br>where $\sum_{i=1}^{p} \boldsymbol{\beta}_i \bar{\boldsymbol{X}}_i =$<br>0.0071931*mean age+-0.1615582*mean gender+0.0268478*mean BMI+<br>1.39069*mean previous fracture+0.1000272*mean parental hip fracture+<br>0.0192416*mean smoking status+0.0374944*mean glucocorticoid use+<br>0.3774416*mean alcohol consumption+0.1097646*mean rheumatoid arthritis+<br>-0.0932063*mean secondary osteoporosis+-0.5110986*mean t-score+<br>-0.4404955*mean (previous fracture*time)
Model 4	where $\sum_{i=1}^{p} \boldsymbol{\beta}_i \boldsymbol{X}_i =$<br>0.0187859*age+-0.2242276*gender+-0.0114226*BMI+1.539469*previous fracture+<br>0.0930207*parental hip fracture+0.0697597*smoking status+0.0509313*glucocorticoid use+<br>0.3635054*alcohol consumption+0.0859078*rheumatoid arthritis+0.5549807*osteoporosis+<br>-0.4478827*(previous fracture*time)<br>where $\sum_{i=1}^{p} \boldsymbol{\beta}_i \bar{\boldsymbol{X}}_i =$<br>0.0187859*mean age+-0.2242276*mean gender+-0.0114226*mean BMI+<br>1.539469*mean previous fracture+0.0930207*mean parental hip fracture+<br>0.0697597*mean smoking status+0.0509313*mean glucocorticoid use+<br>0.3635054*mean alcohol consumption+0.0859078*mean rheumatoid arthritis+<br>0.5549807*mean osteoporosis+-0.4478827*mean (previous fracture*time)

[Table continues on the next page]

Risk Model	Equation

For peer review only

**cont.**	
Model 5	where $\sum_{i=1}^{p} \boldsymbol{\beta}_i \boldsymbol{X}_i =$<br>0.0095458*age+-0.1452714*gender+1.409116*previous fracture+0.0923278*parental hip fracture+ 0.0412411*glucocorticoid use+0.3579932*alcohol consumption+<br>0.1144666*rheumatoid arthritis+ -0.4434596*t-score+-0.4403983*(previous fracture*time)<br>where $\sum_{i=1}^{p} \boldsymbol{\beta}_i \overline{\boldsymbol{X}}_i =$<br>0.0095458*mean age+-0.1452714*mean gender+1.409116*mean previous fracture+<br>0.0923278*mean parental hip fracture+0.0412411*mean glucocorticoid use+<br>0.3579932*mean alcohol consumption+0.1144666*mean rheumatoid arthritis+<br>-0.4434596*mean t-score+-0.4403983*mean (previous fracture*time)

For peer review only

Supplementary Table 2. Crude fracture incidence rates for the derivation and validation datasets.

Risk Factor		Derivation			Validation		
		No of incident cases	Total Person years	Crude Incidence Rate per 10000 person years (95% CI)	No of incident cases	Total Person years	Crude Incidence Rate per 10000 person years (95% CI)
Age Category	40-49	17	1169.9	145.31 (90.33 to 233.74)	12	557.8	215.15 (122.19 to 378.84)
	50-59	70	2534.7	276.17 (218.49 to 349.07)	33	1311.0	251.71 (178.95 to 354.06)
	60-69	93	3062.9	303.63 (247.79 to 372.06)	42	1453.9	288.87 (213.48 to 390.88)
	70-79	83	1906.0	435.46 (351.17 to 539.98)	49	958.7	511.11 (386.29 to 676.26)
	80-89	52	652.7	796.75 (607.13 to 1045.59)	16	347.4	460.56 (282.15 to 751.77)
	90-99	1	26.6	376.51 (53.04 to 2672.85)	-	-	-
Osteoporotic - Hip	No	245	8475.9	289.05 (255.03 to 327.61)	123	4165.1	295.31 (247.48 to 352.40)
	Yes	71	876.8	809.73 (641.68 to 1021.78)	29	463.8	625.28 (434.52 to 899.78)
Osteoporotic - Spine	No	191	7025.8	271.86 (235.91 to 313.28)	111	3475.6	319.37 (265.16 to 384.67)
	Yes	119	2149.6	553.59 (462.55 to 662.55)	39	1089.1	358.08 (261.63 to 490.10)
Gender	Female	266	7417.5	358.61 (318.01 to 404.40)	129	3679.8	350.56 (295.00 to 416.59)
	Male	50	1935.3	258.36 (195.82 to 340.88)	23	949.0	242.36 (161.05 to 364.71)
Parental History Hip Fracture	No	220	6281.5	350.24 (306.88 to 399.71)	118	3108.7	379.58 (316.92 to 454.64)
	Yes	96	3071.3	312.57 (255.90 to 381.79)	34	1520.2	223.66 (159.81 to 313.02)

[Table continues on the next page]

For peer review only

Risk Factor cont.		Derivation No of incident cases	Derivation Total Person years	Derivation Crude Incidence Rate per 10000 person years (95% CI)	Validation No of incident cases	Validation Total Person years	Validation Crude Incidence Rate per 10000 person years (95% CI)
Current Smoker	No	240	7279.1	329.71 (290.53 to 374.18)	103	3513.1	293.19 (241.70 to 355.65)
	Yes	76	2073.7	366.5 (292.71 to 458.90)	49	1115.8	439.16 (331.91 to 581.07)
Alcohol Consumption more than 3 units per day	No	293	8875.9	330.11 (294.39 to 370.15)	140	4399.7	318.2 (269.63 to 375.53)
	Yes	23	476.9	482.33 (320.52 to 725.83)	12	229.2	523.66 (297.39 to 922.09)
Glucocorticoid Use (3 months)	No	279	8184.5	340.89 (303.15 to 383.33)	132	3993.1	330.57 (278.73 to 392.06)
	Yes	37	1168.3	316.7 (229.47 to 437.11)	20	635.8	314.57 (202.95 to 487.59)
Menopause	No	68	1962.8	346.44 (273.15 to 439.39)	29	928.9	312.19 (216.94 to 449.24)
	Yes	198	5454.7	362.99 (315.79 to 417.24)	100	2750.9	363.52 (298.82 to 442.23)
Premature Menopause (<45 years)	No	280	8175.4	342.49 (304.64 to 385.05)	127	4032.1	314.97 (264.69 to 374.81)
	Yes	36	1177.4	305.76 (220.56 to 423.89)	25	596.8	418.92 (283.07 to 619.97)
BMI - low (<18.5)	No	289	8876.1	325.59 (290.14 to 365.38)	141	4367.0	322.87 (273.75 to 380.82)
	Yes	11	187.0	588.16 (325.72 to 1062.04)	4	124.0	322.63 (121.09 to 859.62)
Rheumatoid Arthritis	No	289	8457.8	341.70 (304.49 to 383.45)	139	4135.1	336.15 (284.66 to 396.94)
	Yes	27	895.0	301.69 (206.90 to 439.93)	13	493.8	263.29 (152.88 to 453.43)

[Table continues on the next page]

For peer review only

Risk Factor cont.		Derivation No of incident cases	Derivation Total Person years	Derivation Crude Incidence Rate per 10000 person years (95% CI)	Validation No of incident cases	Validation Total Person years	Validation Crude Incidence Rate per 10000 person years (95% CI)
Secondary Osteoporosis	No	262	7846.5	333.9 (295.83 to 376.89)	122	3853.4	316.60 (265.12 to 378.07)
	Yes	54	1506.2	358.51 (274.58 to 468.10)	30	775.4	386.89 (270.51 to 553.34)
Previous Fracture	No	144	6832.0	210.77 (179.01 to 248.17)	63	3319.4	189.79 (148.26 to 242.95)
	Yes	172	2520.8	682.32 (587.61 to 792.31)	89	1309.4	679.69 (552.18 to 836.64)
Previous Fracture, detail	None	144	6832.0	210.77 (179.01 to 248.17)	63	3319.4	189.79 (148.26 to 242.95)
	1 fracture	105	1919.6	546.99 (451.76 to 662.29)	52	1049.8	495.36 (377.47 to 650.07)
	2-4 fractures	57	557.9	1021.62 (788.04 to 1324.45)	33	236.2	1397.28 (993.37 to 1965.44)
	5+ fractures	10	43.2	2311.27 (1243.59 to 4295.60)	4	23.5	1701.81 (638.72 to 4534.31)
**Total**		**316**	**9352.8**	**337.87 (302.60 to 377.25)**	**152**	**4628.8**	**328.38 (280.11 to 384.96)**

For peer review only

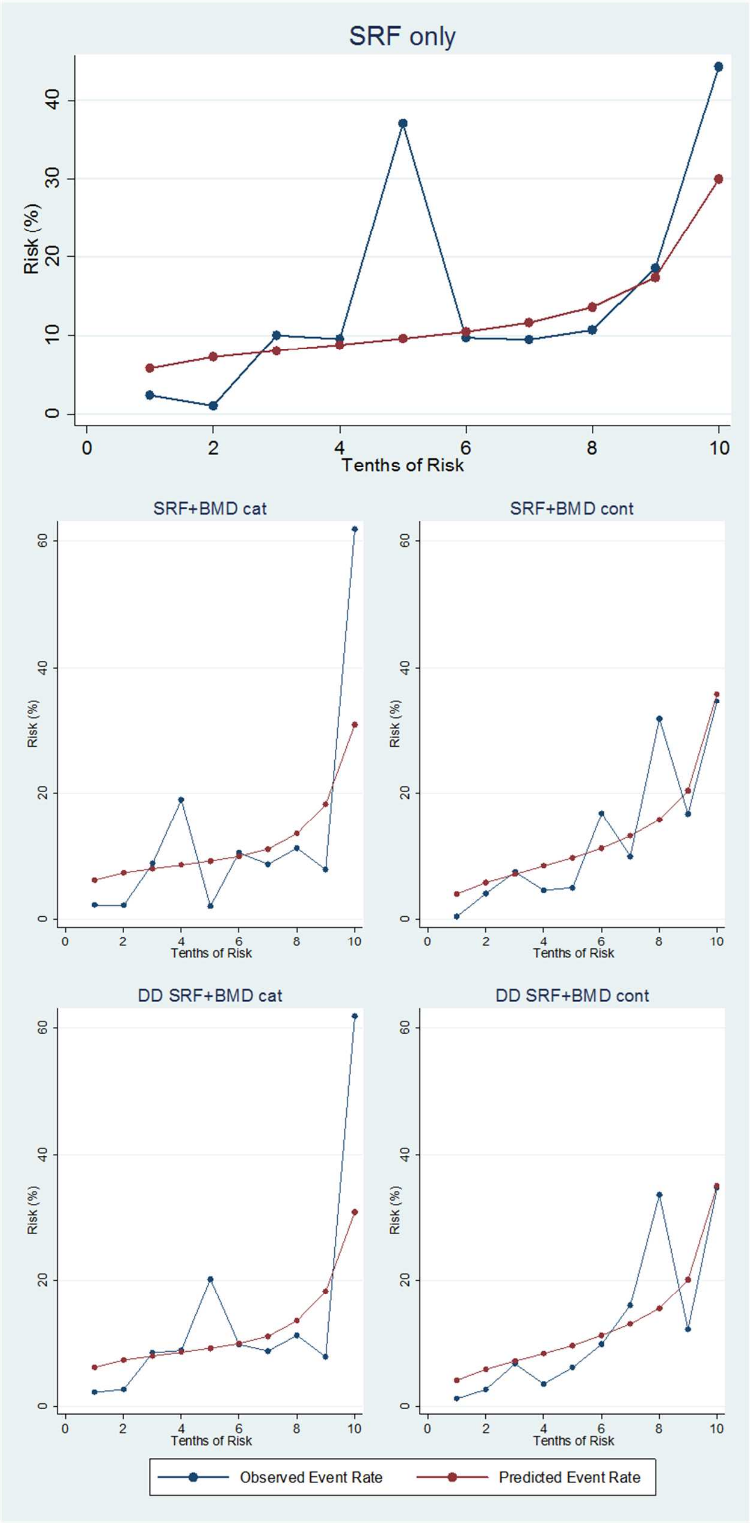

Supplementary Figure 1 Predicted and Observed risk by 10th of predicted risk for each risk prediction model in the derivation dataset.

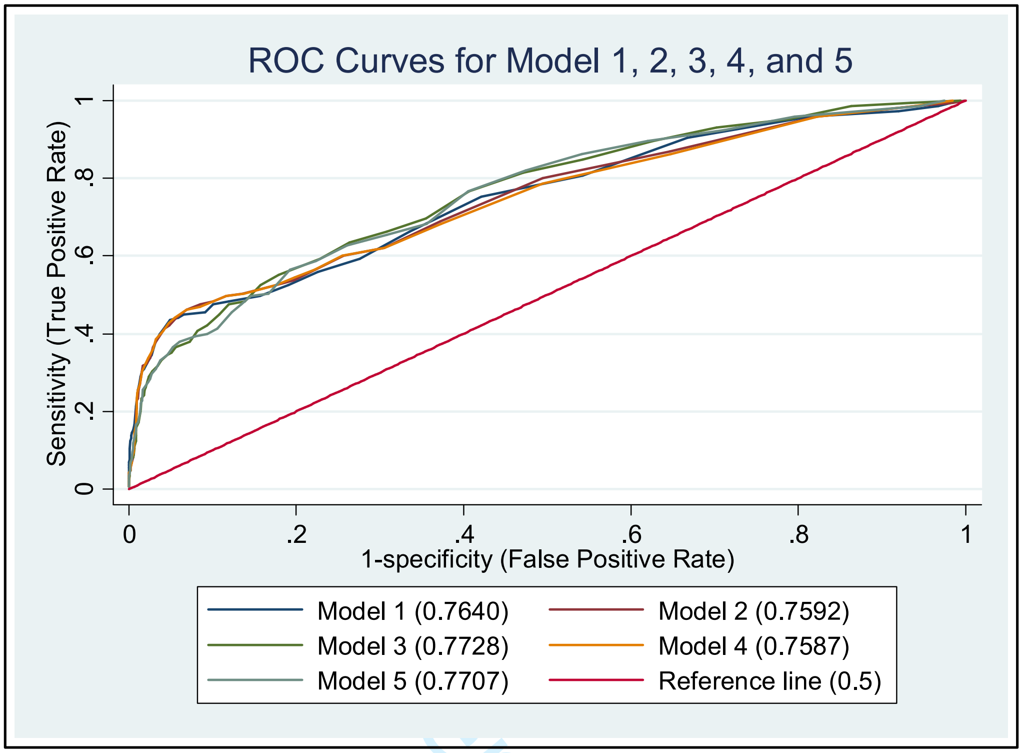

Supplementary Figure2. Receiver Operating Characteristic curve for each model using the derivation dataset, with related Harrell's C-Index (analogous to the AUC).

For peer review only

TRIPOD Checklist: Prediction Model Development and Validation

Section/Topic	Item		Checklist Item	Page
**Title and abstract**				
Title	1	D;V	Identify the study as developing and/or validating a multivariable prediction model, the target population, and the outcome to be predicted.	1
Abstract	2	D;V	Provide a summary of objectives, study design, setting, participants, sample size, predictors, outcome, statistical analysis, results, and conclusions.	3
**Introduction**				
Background and objectives	3a	D;V	Explain the medical context (including whether diagnostic or prognostic) and rationale for developing or validating the multivariable prediction model, including references to existing models.	4
	3b	D;V	Specify the objectives, including whether the study describes the development or validation of the model or both.	4
**Methods**				
Source of data	4a	D;V	Describe the study design or source of data (e.g., randomized trial, cohort, or registry data), separately for the development and validation data sets, if applicable.	5
	4b	D;V	Specify the key study dates, including start of accrual; end of accrual; and, if applicable, end of follow-up.	5
Participants	5a	D;V	Specify key elements of the study setting (e.g., primary care, secondary care, general population) including number and location of centres.	5
	5b	D;V	Describe eligibility criteria for participants.	5
	5c	D;V	Give details of treatments received, if relevant.	-
Outcome	6a	D;V	Clearly define the outcome that is predicted by the prediction model, including how and when assessed.	5
	6b	D;V	Report any actions to blind assessment of the outcome to be predicted.	-
Predictors	7a	D;V	Clearly define all predictors used in developing or validating the multivariable prediction model, including how and when they were measured.	5
	7b	D;V	Report any actions to blind assessment of predictors for the outcome and other predictors.	-
Sample size	8	D;V	Explain how the study size was arrived at.	-
Missing data	9	D;V	Describe how missing data were handled (e.g., complete-case analysis, single imputation, multiple imputation) with details of any imputation method.	6
Statistical analysis methods	10a	D	Describe how predictors were handled in the analyses.	5
	10b	D	Specify type of model, all model-building procedures (including any predictor selection), and method for internal validation.	6
	10c	V	For validation, describe how the predictions were calculated.	6/7
	10d	D;V	Specify all measures used to assess model performance and, if relevant, to compare multiple models.	6/7
	10e	V	Describe any model updating (e.g., recalibration) arising from the validation, if done.	6/7
Risk groups	11	D;V	Provide details on how risk groups were created, if done.	7
Development vs. validation	12	V	For validation, identify any differences from the development data in setting, eligibility criteria, outcome, and predictors.	6
**Results**				
Participants	13a	D;V	Describe the flow of participants through the study, including the number of participants with and without the outcome and, if applicable, a summary of the follow-up time. A diagram may be helpful.	8
	13b	D;V	Describe the characteristics of the participants (basic demographics, clinical features, available predictors), including the number of participants with missing data for predictors and outcome.	8
	13c	V	For validation, show a comparison with the development data of the distribution of important variables (demographics, predictors and outcome).	9/10
Model development	14a	D	Specify the number of participants and outcome events in each analysis.	8
	14b	D	If done, report the unadjusted association between each candidate predictor and outcome.	10
Model specification	15a	D	Present the full prediction model to allow predictions for individuals (i.e., all regression coefficients, and model intercept or baseline survival at a given time point).	18-21
	15b	D	Explain how to the use the prediction model.	18
Model performance	16	D;V	Report performance measures (with CIs) for the prediction model.	12/13
Model-updating	17	V	If done, report the results from any model updating (i.e., model specification, model performance).	12/13
**Discussion**				
Limitations	18	D;V	Discuss any limitations of the study (such as nonrepresentative sample, few events per predictor, missing data).	14/15
Interpretation	19a	V	For validation, discuss the results with reference to performance in the development data, and any other validation data.	13
	19b	D;V	Give an overall interpretation of the results, considering objectives, limitations, results from similar studies, and other relevant evidence.	14/15
Implications	20	D;V	Discuss the potential clinical use of the model and implications for future research.	15
**Other information**				
Supplementary information	21	D;V	Provide information about the availability of supplementary resources, such as study protocol, Web calculator, and data sets.	18-26
Funding	22	D;V	Give the source of funding and the role of the funders for the present study.	2

*Items relevant only to the development of a prediction model are denoted by D, items relating solely to a validation of a prediction model are denoted by V, and items relating to both are denoted D;V. We recommend using the TRIPOD Checklist in conjunction with the TRIPOD Explanation and Elaboration document.

For peer review only - http://bmjopen.bmj.com/site/about/guidelines.xhtml

BMJ Open

Does Bone Mineral Density Improve the Predictive Accuracy of Fracture Risk Assessment? A Prospective Cohort Study in Northern Denmark.

Journal:	*BMJ Open*
Manuscript ID	bmjopen-2017-018898.R1
Article Type:	Research
Date Submitted by the Author:	22-Nov-2017
Complete List of Authors:	Dhiman, Paula; University of Nottingham, School of Medicine<br>Andersen, Stig; Aalborg Universitetshospital, Department of Clinical Medicine<br>Vestergaard, Peter; Aalborg Universitetshospital, Department of Clinical Medicine and Endocrinology<br>Masud, Tahir ; University of Nottingham School of Medicine<br>Qureshi, Nadeem; University of Nottingham, Division of Primary Care
**Primary Subject Heading**:	General practice / Family practice
Secondary Subject Heading:	Research methods, Geriatric medicine, Public health
Keywords:	risk prediction, osteoporosis, fracture, predictive accuracy

SCHOLARONE™
Manuscripts

For peer review only

Title

Does Bone Mineral Density Improve the Predictive Accuracy of Fracture Risk Assessment? A Prospective Cohort Study in Northern Denmark.

Authors list (names, affiliations, and position)

Paula Dhiman, Stig Andersen, Peter Vestergaard, Tahir Masud, Nadeem Qureshi

Division of Primary Care, University of Nottingham, Nottingham, NG7 2RD, UK

Paula Dhiman (Research Fellow)

Geriatric Medicine, Department of Clinical Medicine, Aalborg University Hospital, Hobrovej 18-22 Aalborg University Hospital, 9000 Aalborg, Denmark

Stig Andersen (Clinical Professor)

Department of Clinical Medicine and Endocrinology, Aalborg University Hospital Mølleparkvej 4, DK-9000 Aalborg, Denmark

Peter Vestergaard (Clinical Professor)

Geriatric Medicine, South Block, B Floor, Queens Medical Centre Campus, University of Nottingham, Nottingham NG7 2UH, UK

Tahir Masud (Honorary Professor)

Division of Primary Care, University of Nottingham, Nottingham, NG7 2RD, UK

Nadeem Qureshi (Clinical Professor)

Correspondence to: P Dhiman paula.dhiman@nottingham.ac.uk

Copyright

The Corresponding Author has the right to grant on behalf of all authors and does grant on behalf of all authors, a worldwide license to the Publishers and its licensees in perpetuity, in all forms, formats and media (whether known now or created in the future), to i) publish, reproduce, distribute, display and store the Contribution, ii) translate the Contribution into other languages, create adaptations, reprints, include within collections and create summaries, extracts and/or, abstracts of the Contribution, iii) create any other derivative work(s) based on the Contribution, iv) to exploit all subsidiary rights in the Contribution, v) the inclusion of electronic links from the Contribution to third party material where-ever it may be located; and, vi) license any third party to do any or all of the above.

Exclusive License Statement

The Corresponding Author has the right to grant on behalf of all authors and does grant on behalf of all authors, an exclusive licence (or non exclusive for government employees) on a worldwide basis to the BMJ Publishing Group Ltd to permit this article (if accepted) to be published in BMJ editions and any other BMJPGL products and sublicenses such use and exploit all subsidiary rights, as set out in our licence.

For peer review only - http://bmjopen.bmj.com/site/about/guidelines.xhtml

Competing Interests

We have read and understood BMJ policy on declaration of interests and declare that we have no competing interests.

All authors have completed the ICMJE uniform disclosure form at www.icmje.org/coi_disclosure.pdf and declare: no support from any organisation for the submitted work; no financial relationships with any organisations that might have an interest in the submitted work in the previous three years; no other relationships or activities that could appear to have influenced the submitted work.

Transparency declaration

The lead author (Dr Dhiman) affirms that this manuscript is an honest, accurate, and transparent account of the study being reported; that no important aspects of the study have been omitted; and that any discrepancies from the study as planned (and, if relevant, registered) have been explained.

Ethical Approval

Ethics approval was given through the Region of North Jutland's from the Danish Data Protection Agency ("paraplyanmeldelse 2008-58-0028").

Funding

This study was funded through the National Institute for Health Research (NIHR), School for Primary Care Research (SPCR).

NIHR acknowledgement

This paper presents independent research funded by the National Institute for Health Research School for Primary Care Research (NIHR SPCR).

Disclaimer

The views expressed are those of the author(s) and not necessarily those of the NIHR, the NHS or the Department of Health.

Patient Involvement

Patients were not involved in the development of this research question.

Data Sharing Statement

Data sharing: Technical appendix and statistical code is available from the corresponding author at paula.dhiman@nottingham.ac.uk. Due to restrictions by the Danish Data Protection Agency, data can only be shared on an aggregated level and by special permission.

Contributorship

Contributors: PD wrote the statistical analysis plan, cleaned and analysed the data, and drafted and revised the paper. PV and SA provided the AURORA dataset for analysis and linked patients to the National Patient Registry of Denmark, they also reviewed and revised the draft paper. NQ and TM provided clinical expertise, and reviewed and revised the draft paper.

For peer review only - http://bmjopen.bmj.com/site/about/guidelines.xhtml

Abstract:

Objective: To evaluate the added predictive accuracy of bone mineral density (BMD) to fracture risk assessment.

Design: Prospective cohort study using data between 01/01/2010 and 31/12/2012.

Setting: North Denmark Osteoporosis Clinic of referred patients presenting with at least one fracture risk factor to the referring doctor.

Participants: Patients aged 40-90 years; had BMD T-score recorded at the hip; and not taking osteoporotic preventing drugs for more than 1-year prior to baseline.

Main outcome measures: Incident diagnoses of osteoporotic fractures (hip, spine, forearm, humerus, and pelvis) were identified using the National Patient Registry of Denmark during 01/01/2012-01/01/2014. Cox regression was used to develop a fracture model based on predictors in the Fracture Risk Assessment Tool (FRAX®), with and without, binary and continuous BMD. Change in Harrell's C-Index and Reclassification tables were used to describe the added statistical value of BMD.

Results: Adjusting for predictors included in FRAX®, osteoporotic patients (T-score≤-2.5) had 75% higher hazard of a fracture compared to patients with higher BMD (HR:1.75 (95% CI:1.28 to 2.38)). Forty-percent lower hazard was found per unit increase in continuous BMD T-score (HR:0.60 (95% CI:0.52 to 0.69)).

Accuracy improved marginally, and Harrell's C-Index increased by 1.2% when adding continuous BMD (0.76 to 0.77). Reclassification tables showed continuous BMD shifted 529 patients into different risk categories; 292 of these were reclassified correctly (57%; 95% CI:55% to 64%). Adding binary BMD however no improvement: Harrell's C-Index decreased by 0.6%.

Conclusions: Continuous bone mineral density marginally improves fracture risk assessment. Importantly, this was only found when using continuous BMD measurement for osteoporosis. It is suggested that future focus should be on evaluation of this risk factor using routinely collected data, and on the development of more clinically relevant methodology to assess the added value of a new risk factor

For peer review only - http://bmjopen.bmj.com/site/about/guidelines.xhtml

Article Summary

Strengths and Limitations:

- Addresses a research question recommended by The National Institute for Health and Care Excellence to investigate the added value of bone mineral density to fracture risk prediction.
- Investigates bone mineral density in both the commonly used, binary, and continuous format.
- Presents changes in calibration, discrimination, and reclassification to describe the added value of bone mineral density.
- Uses robustly collected data from Northern Denmark, with 3.2% missing data.
- As data is from a North Danish population, with at least one fracture risk factor, this limits generalisability of the results.

For peer review only

For peer review only - http://bmjopen.bmj.com/site/about/guidelines.xhtml

Introduction

Osteoporosis causes over 8.9 million fractures worldwide, of which over 4.5 million occur in the USA and Europe, and account for 2.8 million disability adjusted life years (1). Further, 1.2 million disability adjusted life years are accounted for by hip fractures, which are projected to increase to 6 million by 2050 (2).

Given this burden, and treatment options for osteoporosis, identifying patients at risk of an osteoporotic fracture is high priority amongst health policymakers to reduce the risk of future fracture (3). Risk prediction tools have been developed to aid in the identification of patients at risk. For example, the Fracture Risk Assessment Tool (FRAX®) and QFracture® are commonly used to assess fracture risk in patients based on pre-defined risk factors.

Bone mineral density (BMD), a measurement used to aid diagnosis of osteoporosis, has also been identified as a fracture risk factor (4-7) . Unlike some other fracture risk factors, treatment options (e.g. bisphosphonate medication) are available that reduces the fracture risk markedly when treatment is initiated based on low BMD.

English National guidelines (The National Institute for Health and Care Excellence (NICE)) for fracture risk assessment recommend treatment of osteoporosis to prevent fractures but have not included BMD as a mandatory risk factor for fracture risk prediction tools to incorporate (8). This is partly due to the lack of robust evidence and limited generalisability of current research, which has particularly focused on evaluating BMD in postmenopausal women evaluating the added value of BMD to existing fracture risk factors (5-7).

The National Institute of Clinical Health and Excellence also recognise this gap in the evidence and have recommended research to assess the added value of BMD as a risk factor in fracture risk assessment (9).

The aim of this study is to assess the value of BMD measurement in addition to the standard fracture risk factors used in the FRAX® risk model using a robustly collected prospective cohort.

For peer review only

For peer review only - http://bmjopen.bmj.com/site/about/guidelines.xhtml

Methods

This paper has been written in accordance to the TRIPOD checklist.

Patient Involvement

Patients were not involved in the development of this research question and were not involved in the design of this study.

Study Design and Data Source

A prospective cohort study was conducted using patients from the Aalborg University Hospital Record for Osteoporosis Risk Assessment (AURORA) dataset; patients were followed up using the National Patient Registry of Denmark.

The AURORA dataset consists of patients attending the Osteoporosis Clinic at Aalborg University Hospital after a referral from their primary care physician. A referral was offered to patients with at least one risk factor for osteoporosis (low BMI, previous fracture, parental hip fracture, smoking status, alcohol consumption, glucocorticoid use, rheumatoid arthritis, and secondary osteoporosis) or if they were aged 80 years and above. Further detail of the data collection has been described elsewhere (10). The Danish National Patient Registry which collects inpatient and outpatient data from all Danish hospitals, was linked to the AURORA dataset through unique patient identifiers

Ethics approval was given through the Region of North Jutland's from the Danish Data Protection Agency ("paraplyanmeldelse 2008-58-0028").

Cohort selection

Data collection for AURORA began 1st January 2010 and was collected for 3 years (up to 31st December 2012). Patients were included if they were aged 40-90 years; had a BMD T-score at the hip; and were not taking any osteoporotic preventing drugs or any bone sparing drugs for more than one year prior to baseline.

Primary Outcome

The primary outcome measure was an incident osteoporotic fracture during follow up (01/01/2012 to 01/01/2014); defined as a diagnosis of a fracture at the hip, spine, forearm, humerus, and pelvis. Fractures at these sites resulting from traffic, work, and sports related accidents were excluded from the study. Relevant fractures were identified in the Danish National Patient Registry, using the International Statistical Classification of Diseases, 10th Version codes (ICD-10 codes), which was developed using recognised database methodology for each fracture (11).

Fracture risk factors

Fracture risk factors, used in the FRAX® risk prediction model, were extracted at baseline. They were: age; gender; height (m); weight (kg); previous fracture; parental history of hip fracture, current smoking status; current alcohol consumption; glucocorticoid use (currently exposed for 3+ months); rheumatoid arthritis; and secondary osteoporosis (includes type I diabetes; osteogenesis imperfecta in adults; untreated, long standing hyperthyroidism;

For peer review only - http://bmjopen.bmj.com/site/about/guidelines.xhtml

hypogonadism; premature menopause (<45 years); chronic malnutrition; malabsorption; and chronic liver disease).

Bone Mineral Density

DXA scans were performed by trained technicians using Hologic Discovery A (Bedford, MA, USA). A daily QC programme was in place and in vivo CV using repositioning of patients was <1%. Total hip BMD was used as region of interest. Bone mineral density was added to the fracture risk prediction model twice, firstly, as a continuously measured T-score value, and secondly, as a binary risk factor, dichotomised at/above T-score threshold for osteoporosis and below threshold, -2.5 in T-score (manufacturers' normal range using normal material from T Kelly et al (12)) based on World Health Organisation (WHO) classifications (13). Calculated T-scores were gender specific.

Statistical Analysis

A complete case analysis was performed on the data; 3.2% of data was missing. The AURORA dataset was split into two using recognised methodology (14); where a random number was assigned to patients and based on a cut off, two-thirds was used to derive the risk models, and the remaining third was used to validate them.

Model derivation

Three Cox proportional hazards models were developed for the primary outcome, using a complete case analysis on the derivation dataset:

Model 1. Standard fracture risk factors only (without BMD)
Model 2. Standard fracture risk factors (with binary BMD)
Model 3. Standard fracture risk factors (with continuous BMD)

Graphical methods were used (log-log plots) to assess the proportional hazards assumption, and risk factors violating this assumption were added to the model as a time varying covariate.

Recognised methodology used in research studies was used to build the 3 risk prediction models (15, 16); the Kaplan Meier method was used to obtain 4-year fracture risk estimates for patients. Further detail on the conversion of the Cox proportional hazards models to risk prediction models has been provided in Supplementary Table 1.

Validation of Models

Four-year fracture risk was calculated from each model and the predictive performance of each risk prediction model was assessed by measures describing calibration, discrimination, and reclassification. These metrics were assessed using the validation cohort.

Calibration measures how well the predicted risk agrees with observed risk in the data. It plots the mean predicted and observed risk of fracture for each decile of predicted risk. The observed risk of fracture was derived from the 4 year Kaplan-Meier estimate. Good calibration indicates the predicted risk is close to the observed risk of the outcome.

Discrimination measures how well the risk prediction model differentiates between patients who have or have not observed the event in the study. This was quantified by the area under

For peer review only - http://bmjopen.bmj.com/site/about/guidelines.xhtml

the receiver operating characteristic (ROC) curve (AUC), given by Harrell's C-Index with higher values indicating better discrimination.

Reclassification tables (17) measures movement between risk categories when adding a new risk factor. Threshold for treatment at 4 years was set at a fracture risk level of 8.5%; to be comparable to the treatment threshold of 20% at 10 years. This was presented by the total percent of patients reclassified (incorrectly and correctly), and also the Net Reclassification Index (NRI) (18, 19). The NRI gives the net calculation of the changes in the right direction and a higher NRI indicates a better reclassifying model.

All analyses were carried out using Stata (version 12) (20).

For peer review only

For peer review only - http://bmjopen.bmj.com/site/about/guidelines.xhtml

Results

Characteristics of the data

The AURORA collected data on 7,912 patients; 1,795 patients were excluded comprising, 440 not aged between 40-90 years at baseline; 156 not having a recorded T-score value for the total hip at baseline; and 1,199 patients were taking anti-osteoporotic drug therapy for more than one year prior to baseline.

The study sample consisted of 6,117 patients; predominantly female (79.6%), and patients with a mean age of 62.9 (SD: 10.9) years. Two-thirds of this sample (n=4,093) was used for the derivation dataset and one-third (n=2,094) was used for the validation dataset. Table 1 presents the baseline characteristics of the study by derivation and validation dataset, and shows little difference between the datasets.

Patients in the derivation dataset had a median follow up time of 2.30 years [1.57, 2.99], and observed 318 (7.8%) osteoporotic fractures during follow up. Of these, 316 fractures were eligible for the analysis (2 patients had a fractures on or prior to baseline and were excluded). Patients contributed 9352.8 person years of observation, giving a total incidence rate of 337.87 per 10,000 person years (95% CI:302.60 to 377.25).

Fractures during follow up were predominantly found in the forearm (27.0%) and hip (17.9%). Higher fracture incidence rates were found in patients classed as osteoporotic, based on their T-score at both the total hip (809.73 per 10,000 person years (95% CI:641.68 to 1021.78)) and spine (L1-L4) (553.59 per 10,000 person years (95% CI:462.55 to 662.55)) (Supplementary Table 2).

For peer review only - http://bmjopen.bmj.com/site/about/guidelines.xhtml

Table 1. Baseline characteristics of the derivation and validation datasets, including missing data.

Characteristic			Derivation (n=4,093)		Validation (n=2,024)	
			No	%	No	%
Gender	Female		3,266	79.8	1,602	79.2
	Male		827	20.2	422	20.8
Osteoporotic (Hip DXA)	No		3,683	90.0	1,820	89.9
	Yes		410	10.0	204	10.1
Osteoporotic Status (based on UK guidelines)	Normal		1,886	46.1	927	45.8
	Osteopenic		1,797	43.9	893	44.1
	Osteoporotic		410	10.0	204	10.1
Previous Fracture	No		2,935	71.7	1,423	70.3
	Yes		1,158	28.3	601	29.7
No of Previous Fractures	None		2,935	71.7	1,423	70.3
	1 fracture		862	21.1	467	23.1
	2-4 fractures		270	6.6	122	6.0
	5+ fractures		26	0.6	12	0.6
Parental History of Hip Fracture	No		2,755	67.3	1,359	67.1
	Yes		1,338	32.7	665	32.9
Current Smoking Status	other (non/ex)		3,182	77.7	1,529	75.5
	smoker		911	22.3	495	24.5
Alcohol Consumption	≤3 units per day		3,875	94.7	1,923	95.0
	>3 units per day		218	5.3	101	5.0
Glucocorticoid Use	No		3,577	87.4	1,741	86.0
	Yes		516	12.6	283	14.0
Rheumatoid Arthritis	No		3,686	90.1	1,801	88.0
	Yes		407	9.9	223	11.0
Other Bone Affecting Disease	No		2,382	58.2	1,139	56.3
	Yes		1,711	41.8	885	43.7
Secondary Osteoporosis	No		3,438	84.0	1,689	83.5
	Yes		655	16.0	335	16.6
	By disease					
	Type 1 diabetes	No	4,010	98.0	1,981	97.9
		Yes	83	2.0	43	2.1
	Osteogenesis	No	4,093	100	2,024	100
		Yes	0	0	0	0
	Hyperthyroidism	No	4,089	99.9	2,023	99.9
		Yes	4	0.1	1	0.1
	Malnutrition	No	4,090	99.9	2,023	99.9
		Yes	3	0.1	1	0.1
	Chronic Liver Disease	No	4,006	97.9	1,979	97.8
		Yes	87	2.1	45	2.2
	Menopause (Females only)**	No	853	26.1	405	25.3
		Yes	2,413	73.9	1,197	74.7

For peer review only - http://bmjopen.bmj.com/site/about/guidelines.xhtml

	Premature Menopause (<45years)***	No	1,904	78.9	941	78.6
		Yes	509	21.1	256	21.4
			**Mean**	**SD**	**Mean**	**SD**
Age (years)			62.9	10.9	63.0	11.0
Weight (kg)			72.1	15.5	72.2	15.9
	Missing		47	1.2	12	0.6
Height (m)			1.7	0.1	1.7	0.1
	Missing		131	3.2	61	3.0
BMI			26.4	5.0	26.4	5.1
	Missing		135	3.3	63	3.1
Hip DXA T-score			-1.1	1.1	-1.2	1.1

*out of patients with a fracture
**proportion out of respective number of females
***proportion out of respective number of females with menopause

Model development

The unadjusted analysis showed statistically significant association between BMD (continuous and binary) and osteoporotic fracture (p<0.001). Significant associations with fracture were also found with age (p<0.001), previous fracture (p<0.001), BMI (p=0.03), and gender (p=0.05). Further, a time-varying effect was found in patients with a previous fracture; hazard of a subsequent fracture was highest in the first year during follow up and decreased per year of follow up (p<0.001).

The adjusted analysis is presented in Table 2. Model 1 showed that of the standard risk factors, age and previous fracture were significantly associated with fracture; hazard of fracture increased by 2% per year increase in age (HR=1.02; 95% CI: 1.01 to 1.04); and increased almost 5 fold in patients with a previous fracture at time 0 years (HR=4.88; 95% CI: 3.37 to 7.08).

Hazard of fracture increased by 75% for patients classed as osteoporotic by their BMD score (Model 2, HR=1.75; 95% CI: 1.28 to 2.38). Hazard of fracture also decreased by 40% per SD improvement in BMD T-score (Model 3, HR=0.60; 95% CI: 0.52 to 0.69).

For peer review only - http://bmjopen.bmj.com/site/about/guidelines.xhtml

Table 2. Multivariate analysis for osteoporotic fracture in the derivation cohort. Data are adjusted hazard ratios and 95% confidence intervals.

**Risk Factor**		**Adjusted Hazard Ratio (95% CI)**		
		**Model 1: Standard Risk Factors only**	**Model 2: Standard Risk Factors + BMD (categorical)**	**Model 3: Standard Risk Factors + BMD (continuous)**
Age (years)		1.02 (1.01 to 1.04)	1.02 (1.01 to 1.03)	1.01 (1.00 to 1.02)
Gender	Female	Ref	Ref	Ref
	Male	0.75 (0.54 to 1.04)	0.80 (0.57 to 1.10)	0.85 (0.61 to 1.18)
BMI		0.98 (0.95 to 1.00)	0.99 (0.96 to 1.01)	1.03 (1.00 to 1.05)
Previous Fracture	No	Ref	Ref	Ref
	Yes (time = 0 years)	4.88 (3.37 to 7.08)	4.67 (3.21 to 6.78)	4.02 (2.76 to 5.84)
Parental History of Hip Fracture	No	Ref	Ref	Ref
	Yes	1.08 (0.83 to 1.40)	1.10 (0.85 to 1.42)	1.11 (0.85 to 1.43)
Current Smoker	No	Ref	Ref	Ref
	Yes	1.12 (0.85 to 1.47)	1.08 (0.82 to 1.42)	1.02 (0.77 to 1.34)
Alcohol Consumption (>3 units/day)	No	Ref	Ref	Ref
	Yes	1.41 (0.90 to 2.21)	1.44 (0.92 to 2.25)	1.04 (0.72 to 1.49)
Glucocorticoid Use	No	Ref	Ref	Ref
	Yes	1.08 (0.75 to 1.55)	1.05 (0.73 to 1.51)	1.46 (0.93 to 2.28)
Rheumatoid Arthritis	No	Ref	Ref	Ref
	Yes	1.10 (0.73 to 1.65)	1.09 (0.72 to 1.64)	1.12 (0.74 to 1.68)
Secondary Osteoporosis	No	Ref	Ref	Ref
	Yes	0.99 (0.73 to 1.35)	0.97 (0.71 to 1.32)	0.91 (0.67 to 1.24)
Osteoporotic	No	Ref	Ref	Ref
	Yes	-	1.75 (1.28 to 2.38)	-
Hip DXA T-score (SD)		-	-	0.60 (0.52 to 0.69)
Previous Fracture (TVC*)		0.64 (0.49 to 0.83)	0.64 (0.49 to 0.83)	0.64 (0.50 to 0.84)

*TVC = time varying covariate, value is interaction effect and 95% CI.

For peer review only - http://bmjopen.bmj.com/site/about/guidelines.xhtml

Model Validation

The 4-year predicted risk of fracture was calculated for all patients in the validation dataset; this was compared to the observed fracture outcome within the 4 year follow up.

Calibration and Discrimination

Calibration plots suggested some improvement when adding BMD measurement; particularly when including continuous BMD T-score measurement (Model 3; Supplementary Figure 1).

The largest change in discrimination was found when adding continuous BMD measurement to standard risk factors; Harrell's C-Index increased by 1.17% (Table 3). However, binary BMD measurement, as a measure for osteoporotic patients, was found to reduce Harrell's C-Index by -0.65%.

Table 3. Harrell's C-Index for Model 1, 2, 3, 4, and 5.

Model	Harrell's C-Index	Change in Harrell's C-Index (% change)*
**Model 1**: Standard fracture risk factors only (without BMD)	0.764 (0.718 to 0.810)	-
**Model 2**: Standard fracture risk factors only (with binary BMD)	0.759 (0.712 to 0.806)	-0.005 (-0.65%)
**Model 3**: Standard fracture risk factors only (with continuous BMD)	0.773 (0.732 to 0.814)	0.009 (1.17%)

*All change is measures against Model 1.

Reclassification

Reclassification tables suggested that adding continuous BMD measurement improved classification of patients into their correct risk categories. This was not found when adding binary BMD. Table 4 presents the reclassification table for Model 1 (standard fracture risk factors only) and Model 3 (standard risk factors with continuous BMD), using the 8.5% pre-specified risk threshold.

For peer review only - http://bmjopen.bmj.com/site/about/guidelines.xhtml

Table 4. Risk Reclassification Table comparing Model 1 (standard fracture risk factors alone) to Model 3 (standard fracture risk factors with continuous BMD measurement), using a clinical 8.5% risk cut off.

			Model 3: SRF with continuous BMD			
Model 1: SRF without BMD			<8.5%	≥8.5%	Total	Total No.(%) Reclassified
	<8.5%	No	391	227	618	227 (36.7%)
		%	63.3%	36.7%		
		No. Events	5	9		
		No. Non events	386	218		
		Observed Event Rate	1.3%	4.0%		
	≥8.5%	No	302	1,040	1,342	302 (22.5%)
		%	22.5%	77.5%		
		No. Events	10	121		
		No. Non events	292	919		
		Observed Event Rate	3.3%	11.6%		
	Total		693	1,267	1,960	529 (27.0%)

Of the 1,960 patients in the validation dataset, 27% (n=529) were reclassified into a different risk category when including continuous BMD into fracture risk prediction. Two percent (9/529) were found to be reclassified correctly into a higher risk group and 55% (292/529) were reclassified correctly into a lower risk group; indicating 22% (292/1342) of patients at high risk in Model 1, not accounting for BMD measurement, were no longer at high risk. The net reclassification improvement when adding continuous BMD to standard risk factors, was 0.03, which resulted from increased specificity (non-event NRI = 4%) and decreased sensitivity (event NRI: -1%) from Model 1 (Table 5).

Table 5. Summary of Net Reclassification Index (NRI) and Integrated Discrimination Index (IDI) for all comparisons between developed fracture risk prediction models.

Comparison	Event NRI	Non-Event NRI	Overall NRI
Model 1 vs. Model 2	-3.45%	2.09%	-0.01
Model 1 vs. Model 3	-0.69%	4.08%	0.03

For peer review only - http://bmjopen.bmj.com/site/about/guidelines.xhtml

Discussion

Summary of Findings

Bone mineral density showed significant association with fracture risk with a 40% decrease for each SD rise in BMD. However, this resulted in small improvements in calibration, discrimination, and reclassification. Despite the limited improvement was found of 1% in discrimination when adding continuous BMD, reclassification tables showed 57% of reclassified patients moving into their correct risk group through improved specificity.

Importantly no improvement was found when adding BMD in a binary format. Our findings are consistent with and corroborate with current literature (7, 21). Specifically, a study conducted in the Netherlands with 4 year follow up, investigating the added value of BMD for hip fractures risk found modest improvement in predictability (21). Further, a more recent study also indicated limited added value of BMD to fracture risk prediction (7).

Strengths and Limitations

Answering Evidence gap

To our knowledge, this is the first study to investigate the added value of BMD in a binary and continuous format, to standard fracture risk factors. It helps inform the NICE research recommendation to assess the added value of BMD to routine fracture risk assessment in primary care (22). It further highlights that the more commonly used for treatment decision making, binary format of BMD resulted in a loss of predictability in fracture risk prediction; based on comparable measures for discrimination and reclassification

Robustness of Data

The prospective cohort was well populated with key standard risk factors recorded: BMI, smoking status and alcohol consumption, and personal and parental fracture history. Other than 3.2% of missing data for BMI, in 6,117 patients, complete data was collected for all risk factors (including BMD T-score recorded at the total hip). Further, the cohort was linked to a national robust electronic health records. This Danish National Patient Registry allowed for outcome fracture to be identified and also provided data on the mechanism for the fracture; this helped more accurately phenotype osteoporotic fractures.

Generalisability

The generalisability is affected in a few ways. Firstly, the findings are based on a Danish cohort. Secondly, AURORA data was collected from patients who presented to their doctor with at least one fracture risk factor and were referred to the osteoporosis clinic; this led to a biased study sample with a higher risk of a fracture and increased age. This could overestimate fracture risk amongst patients in a primary care setting.

Methodology

Due to the increased age of the sample, death becomes a competing risk. However, information on death was not collected and could not be retrieved. This limited the analysis of the data as competing risks could not be accounted for which may again lead to an overestimation of fracture risk (23). However, as an independent study primarily assessing the added value of BMD through deriving and validating the fracture risk prediction models,

For peer review only - http://bmjopen.bmj.com/site/about/guidelines.xhtml

this bias would be present in both analyses to compare derived risk models with and without BMD measurement.

The FRAX risk algorithm has not yet been published, therefore FRAX estimates could not be directly calculated for the cohort. Instead, the FRAX risk model was recalibrated on the dataset with and without BMD added. Further, fracture outcomes in this study included pelvic fractures which are increasingly recognised as low trauma fragility fractures [(24)], and used BMD taken at the total hip instead of at the femur neck as it is the gold standard in Denmark (25).

Internal validation was performed to validate the derived risk prediction models. This may lead to over optimistic results of the performance of the risk models (14). To account for this limitation, a commonly practised method which randomly assigns patients to the derivation and validation datasets was used; further, a similar 1:2 ratio was also used to split the data (26-28).

The study had a 4 year follow up which is shorter than other recognised risk models. To account for this, we adapted the 20% clinical risk threshold for 10 year fracture estimates to 8.5% for 4 year fracture estimates, assuming that risk is constant over time (29, 30).

Traditional methodology assessing the added value to risk factors to existing risk prediction models are criticised to be insensitive to change, to lack interpretability (31-34). This was shown when finding a 1% change in Harrell's C-Index and overlapping confidence intervals between models, limiting the interpretability of results. Reclassification analysis was thus also used to provide more clinically interpretable results.

Clinical implications

The most notable clinical implication is the more routine use of BMD measurement for fracture risk assessment. Further, evidence suggests continuous BMD adds better predictability compared to the binary format.

Future Research

Further research is recommended to evaluate the added value of BMD to fracture risk prediction; in particular in addition to QFracture risk factors and using primary care routinely collected data. However, a brief interrogation into the Clinical Practice Research Datalink, a routinely collected UK primary care database, showed poor availability of BMD measurement in patient records, and thus, strong limitations to potential analyses. Less than 1% of patients had BMD recorded from a sample of 60,658 patients aged 40-90; not on any osteoporotic treatment; and with complete data for age, gender, BMI, smoking status, and alcohol consumption. Thus, prior to UK analysis, BMD recording in primary care databases needs to improve.

Methodologically, as well as assessing the added value of BMD to standard risk factors, we should also explore the option to replace existing fracture risk factors with the BMD measurement; this has rarely been explored in the literature but should be considered in future analyses. We also recommend research to investigate the added value of BMD in a potentially more natural, 3 group format of BMD (osteopenic, normal, osteoporotic).

For peer review only - http://bmjopen.bmj.com/site/about/guidelines.xhtml

In addition, further research is recommended to develop current methodology used to assess the added value of BMD to provide more clinically relevant results, such as cost implications; and to allow for better comparability between new risk factors with respect to their added value, thus improving decision making.

Conclusion

Continuous BMD marginally improves fracture risk assessment. Importantly, this was only found when using continuous BMD measurement for osteoporosis. It seems that prediction models for fragility fracture risk may be improved only marginally, using present risk factor assessment and evaluations. It is suggested that future focus should be on additional risk factors and on the development of more clinically relevant methodology to assess the added value of a new risk factor.

For peer review only

For peer review only - http://bmjopen.bmj.com/site/about/guidelines.xhtml

References

1. Kanis JA on behalf of the World Health Organization Scientific Group (2007). Assessment of osteoporosis at the primary health-care level. Technical Report. World Health Organization Collaborating Centre for Metabolic Bone Diseases, University of Sheffield, UK; 2007.
2. WHO scientific group. Prevention and management of osteoporosis: report of a WHO scientific group. WHO Technical Report Series. 2003.
3. Barry P, Aspray T, Briers K, Collins GS, Compston J, Dockery F, et al. Osteoporosis: assessing the risk of fragility fracture. 2012. Contract No.: CG146.
4. Kanis JA, Borgstrom F, Laet CD, Johansson H, Johnell O, Jonsson B, et al. Assessment of fracture risk. Osteoporosis International. 2005;16(6):581-9.
5. Report of a WHO Study Group. Assessment of fracture risk and its application to screening for postmenopausal osteoporosis. 1994.
6. McCloskey E, Johansson H, Oden A, Kanis JA. Fracture risk assessment. Clinical biochemistry. 2012;45(12):887-93.
7. Briot K, Paternotte S, Kolta S, Eastell R, Felsenberg D, Reid DM, et al. FRAX: Prediction of Major Osteoporotic Fractures in Women from the General Population: The OPUS Study. PLoS ONE. 2013;8(12):e83436.
8. National Institute for Health and Care Excellence. Osteoporosis: assessing the risk of fragility fracture. Short clinical guideline - evidence and recommedation. London: National Clinical Guidance Centre. 2012.
9. National Institute for Health and Care Excellence. Osteoporosis: assessing the risk of fragility fracture. NICE short clinical guideline CG146.; 2012.
10. Andersen S, Laurberg P. Age discrimination in osteoporosis screening-data from the Aalborg University Hospital Record for Osteoporosis Risk Assessment (AURORA). Maturitas. 2014;77(4):330-5.
11. Dave S, Petersen I. Creating medical and drug code lists to identify cases in primary care databases. Pharmacoepidemiol Drug Saf. 2009;18(8):704-7.
12. Kelly TL. Bone mineral density reference databases for American men and women. Journal of Bone and Mineral Research. 1990;5(Suppl 2).
13. Kanis JA, Melton LJr, Christianson C, Johnston CC, Khaltaev N. The diagnosis of osteoporosis. Journal of Bone and Mineral Research. 1994;9(8):1137-41.
14. Moons KGM, Kengne AP, Woodward M, Royston P, Vergouwe Y, Altman DG, et al. Risk prediction models: I. Development, internal validation, and assessing the incremental value of a new (bio)marker. Heart. 2012.
15. Hippisley-Cox J, Coupland C. Derivation and validation of updated QFracture algorithm to predict risk of osteoporotic fracture in primary care in the United Kingdom: prospective open cohort study. Bmj. 2012;344:e3427.
16. Hippisley-Cox J, Coupland C, Vinogradova Y, Robson J, Minhas R, Sheikh A, et al. Predicting cardiovascular risk in England and Wales: prospective derivation and validation of QRISK2. British Medical Journal. 2008;336:1475-82.
17. Cook NR. Use and misuse of the receiver operating characteristic curve in risk prediction. Circulation. 2007;115(7):928-35.
18. Cook NR. Comments on 'Evaluating the added predictive ability of a new marker: From area under the ROC curve to reclassification and beyond' by M. J. Pencina et al., Statistics in Medicine. Statistics in Medicine. 2008;27(2):191-5.
19. Pencina MJ, D'Agostino RB, Sr., Steyerberg EW. Extensions of net reclassification improvement calculations to measure usefulness of new biomarkers. Statistics in Medicine. 2011;30(1):11-21.
20. StataCorp. Stata Statistical Software: Release 12. College Station, TX: StataCorp LP; 2011.
21. Burger H, de Laet CE, Weel AE, Hofman A, Pols HA. Added value of bone mineral density in hip fracture risk scores. Bone. 1999;25(3):369-74.

For peer review only - http://bmjopen.bmj.com/site/about/guidelines.xhtml

22. Barry P, Aspray T, Briers K, Collins GS, Compston J, Dockery F, et al. Osteoporosis: assessing the risk of fragility fracture (NICE Clinical Guidance 146). National Institute for Health and Care Excellence; 2012. Contract No.: CG146.
23. Satagopan JM, Ben-Porat L, Berwick M, Robson M, Kutler D, Auerbach AD. A note on competing risks in survival data analysis. Br J Cancer. 2004;91(7):1229-35.
24. Soles G, Ferguson T. Fragility fractures of the pelvis. Curr Rev Musculoskelet Med. 2012;5:222-8.
25. Abrahamsen B, Stilgren LS, Hermann AP, Tofteng CL, Barenholdt O, Vestergaard P, et al. Discordance between changes in bone mineral density measured at different skeletal sites in perimenopausal women--implications for assessment of bone loss and response to therapy: The Danish Osteoporosis Prevention Study. J Bone Miner Res. 2001;16(7):1212-9.
26. Hippisley-Cox J, Coupland C, Vinogradova Y, Robson J, May M, Brindle P. Derivation and validation of QRISK, a new cardiovascular disease risk score for the United Kingdom: prospective open cohort study. British Medical Journal. 2007;335(7611):136-41.
27. Ridker PM, Buring JE, Rifai N, Cook NR. Development and validation of improved algorithms for the assessment of global cardiovascular risk in women: the Reynolds Risk Score.[Erratum appears in JAMA. 2007 Apr 4;297(13):1433]. Jama. 2007;297(6):611-9.
28. Hippisley-Cox J, Coupland C, Robson J, Brindle P. Derivation, validation, and evaluation of a new QRISK model to estimate lifetime risk of cardiovascular disease: cohort study using QResearch database. British Medical Journal. 2010;341.
29. National Osteoporosis Foundation. Clinician's Guide to Prevention and Treatment of Osteoporosis. Washington, DC; 2013.
30. Thomsen K. Odense Fallers and Osteoporosis Study: Assessment of osteoporosis among older women presenting with falls. Denmark: Odense University Hospital; 2014.
31. McGeechan K, Macaskill P, Irwig L, Liew G, Wong TY. Assessing New Biomarkers and Predictive Models for Use in Clinical Practice A Clinician's Guide. Archives of Internal Medicine. 2008;168(21):2304-10.
32. Steyerberg EW, Pencina MJ, Lingsma HF, Kattan MW, Vickers AJ, Van Calster B. Assessing the incremental value of diagnostic and prognostic markers: a review and illustration. European Journal of Clinical Investigation. 2012;42(2):216-28.
33. Janes H, Pepe MS, Gu W. Assessing the Value of Risk Predictions by Using Risk Stratification Tables. Annals of Internal Medicine. 2008;149(10):751-W162.
34. Cook NR. Use and misuse of the receiver operating characteristic curve in risk prediction - Response. Circulation. 2007;116(6):E134-E.

For peer review only

For peer review only - http://bmjopen.bmj.com/site/about/guidelines.xhtml

Supplementary Information

Beta coefficients from each Cox regression model were used to create each fracture risk prediction model.

Once all 5 models were finalised, their beta coefficients were used to create 5 risk prediction models and calculate risk of fracture for each patient, using the following general equation:

$$\widehat{risk} = 1 - S_0(t)^{exp(\sum_{i=1}^{p} \beta_i X_i - \sum_{i=1}^{p} \beta_i \bar{X}_i)}$$

Where $\mathbf{S_0(t)}$ is the baseline survival rate at follow up time, **t**(for this example, a follow up time of 10 years will be used); beta ($\boldsymbol{\beta_i}$) are the regression coefficients for each included risk factor in the model (**i**); $\mathbf{X_i}$ is the observed data value for each risk factor; $\mathbf{\bar{X}_i}$ is the corresponding mean for each risk factor; and **p** is the total number of risk factors included in the model. Table A1 shows the formula for each risk prediction model explicitly.

For peer review only

Supplementary Table 1. Risk equations to calculate 4 year risk based on patient characteristics for each developed risk model.

General Risk Equation to calculate 4 year risk	$1 - 0.89^{exp(\sum_{i=1}^{p} \beta_i X_i - \sum_{i=1}^{p} \beta_i \bar{X}_i)}$
**Risk Model**	**Equation**
Model 1	where $\sum_{i=1}^{p} \beta_i X_i =$<br>0.0237745*age+-0.2826461*gender+-0.0225011*BMI+1.585278*previous fracture+<br>0.0762559*parental hip fracture+0.1138883*smoking status+0.0773898*glucocorticoid use+<br>0.3465287*alcohol consumption+0.0936966*rheumatoid arthritis+<br>-0.0069432*secondary osteoporosis+-0.4535108*(previous fracture*time)<br>where $\sum_{i=1}^{p} \beta_i \bar{X}_i =$<br>0.0237745*mean age+-0.2826461*mean gender+-0.0225011*mean BMI+<br>1.585278*mean previous fracture+0.0762559*mean parental hip fracture+<br>0.1138883*mean smoking status+0.0773898*mean glucocorticoid use+<br>0.3465287*mean alcohol consumption+0.0936966*mean rheumatoid arthritis+<br>-0.0069432*mean secondary osteoporosis+-0.4535108*mean (previous fracture*time)
Model 2	where $\sum_{i=1}^{p} \beta_i X_i =$<br>0.0186827*age+-0.228784*gender+-0.0113651*BMI+1.540559*previous fracture+<br>0.092011*parental hip fracture+0.0732564*smoking status+0.0508706*glucocorticoid use+<br>0.3649544*alcohol consumption+0.0854353*rheumatoid arthritis+ -0.0346885*secondary<br>osteoporosis+0.5568944*osteoporosis+-0.4481145*(previous fracture*time)<br>where $\sum_{i=1}^{p} \beta_i \bar{X}_i =$<br>0.0186827*mean age+-0.228784*mean gender+-0.0113651*mean BMI+<br>1.540559*mean previous fracture+0.092011*mean parental hip fracture+<br>0.0732564*mean smoking status+0.0508706*mean glucocorticoid use+<br>0.3649544*mean alcohol consumption+0.0854353*mean rheumatoid arthritis+<br>-0.0346885*mean secondary osteoporosis+0.5568944*mean osteoporosis+<br>-0.4481145*mean (previous fracture*time)

[Table continues on the next page]

For peer review only

**Risk Model cont.**	**Equation**
Model 3	where $\sum_{i=1}^{p} \boldsymbol{\beta}_i \boldsymbol{X}_i =$<br>0.0071931*age+-0.1615582*gender+0.0268478*BMI+1.39069*previous fracture+ 0.1000272*parental hip fracture+0.0192416*smoking status+0.0374944*glucocorticoid use+ 0.3774416*alcohol consumption+0.1097646*rheumatoid arthritis+ -0.0932063*secondary osteoporosis+-0.5110986*t-score+-0.4404955*(previous fracture*time)<br>where $\sum_{i=1}^{p} \boldsymbol{\beta}_i \overline{\boldsymbol{X}}_i =$<br>0.0071931*mean age+-0.1615582*mean gender+0.0268478*mean BMI+ 1.39069*mean previous fracture+0.1000272*mean parental hip fracture+ 0.0192416*mean smoking status+0.0374944*mean glucocorticoid use+ 0.3774416*mean alcohol consumption+0.1097646*mean rheumatoid arthritis+ -0.0932063*mean secondary osteoporosis+-0.5110986*mean t-score+ -0.4404955*mean (previous fracture*time)

For peer review only

Supplementary Table 2. Crude fracture incidence rates for the derivation and validation datasets.

Risk Factor		Derivation			Validation		
		No of incident cases	Total Person years	Crude Incidence Rate per 10000 person years (95% CI)	No of incident cases	Total Person years	Crude Incidence Rate per 10000 person years (95% CI)
Age Category	40-49	17	1169.9	145.31 (90.33 to 233.74)	12	557.8	215.15 (122.19 to 378.84)
	50-59	70	2534.7	276.17 (218.49 to 349.07)	33	1311.0	251.71 (178.95 to 354.06)
	60-69	93	3062.9	303.63 (247.79 to 372.06)	42	1453.9	288.87 (213.48 to 390.88)
	70-79	83	1906.0	435.46 (351.17 to 539.98)	49	958.7	511.11 (386.29 to 676.26)
	80-89	52	652.7	796.75 (607.13 to 1045.59)	16	347.4	460.56 (282.15 to 751.77)
	90-99	1	26.6	376.51 (53.04 to 2672.85)	-	-	-
Osteoporotic - Hip	No	245	8475.9	289.05 (255.03 to 327.61)	123	4165.1	295.31 (247.48 to 352.40)
	Yes	71	876.8	809.73 (641.68 to 1021.78)	29	463.8	625.28 (434.52 to 899.78)
Osteoporotic - Spine	No	191	7025.8	271.86 (235.91 to 313.28)	111	3475.6	319.37 (265.16 to 384.67)
	Yes	119	2149.6	553.59 (462.55 to 662.55)	39	1089.1	358.08 (261.63 to 490.10)
Gender	Female	266	7417.5	358.61 (318.01 to 404.40)	129	3679.8	350.56 (295.00 to 416.59)
	Male	50	1935.3	258.36 (195.82 to 340.88)	23	949.0	242.36 (161.05 to 364.71)
Parental History Hip Fracture	No	220	6281.5	350.24 (306.88 to 399.71)	118	3108.7	379.58 (316.92 to 454.64)
	Yes	96	3071.3	312.57 (255.90 to 381.79)	34	1520.2	223.66 (159.81 to 313.02)

[Table continues on the next page]

For peer review only

Risk Factor cont.		Derivation			Validation		
		No of incident cases	Total Person years	Crude Incidence Rate per 10000 person years (95% CI)	No of incident cases	Total Person years	Crude Incidence Rate per 10000 person years (95% CI)
Current Smoker	No	240	7279.1	329.71 (290.53 to 374.18)	103	3513.1	293.19 (241.70 to 355.65)
	Yes	76	2073.7	366.5 (292.71 to 458.90)	49	1115.8	439.16 (331.91 to 581.07)
Alcohol Consumption more than 3 units per day	No	293	8875.9	330.11 (294.39 to 370.15)	140	4399.7	318.2 (269.63 to 375.53)
	Yes	23	476.9	482.33 (320.52 to 725.83)	12	229.2	523.66 (297.39 to 922.09)
Glucocorticoid Use (3 months)	No	279	8184.5	340.89 (303.15 to 383.33)	132	3993.1	330.57 (278.73 to 392.06)
	Yes	37	1168.3	316.7 (229.47 to 437.11)	20	635.8	314.57 (202.95 to 487.59)
Menopause	No	68	1962.8	346.44 (273.15 to 439.39)	29	928.9	312.19 (216.94 to 449.24)
	Yes	198	5454.7	362.99 (315.79 to 417.24)	100	2750.9	363.52 (298.82 to 442.23)
Premature Menopause (<45 years)	No	280	8175.4	342.49 (304.64 to 385.05)	127	4032.1	314.97 (264.69 to 374.81)
	Yes	36	1177.4	305.76 (220.56 to 423.89)	25	596.8	418.92 (283.07 to 619.97)
BMI - low (<18.5)	No	289	8876.1	325.59 (290.14 to 365.38)	141	4367.0	322.87 (273.75 to 380.82)
	Yes	11	187.0	588.16 (325.72 to 1062.04)	4	124.0	322.63 (121.09 to 859.62)
Rheumatoid Arthritis	No	289	8457.8	341.70 (304.49 to 383.45)	139	4135.1	336.15 (284.66 to 396.94)
	Yes	27	895.0	301.69 (206.90 to 439.93)	13	493.8	263.29 (152.88 to 453.43)

[Table continues on the next page]

For peer review only - http://bmjopen.bmj.com/site/about/guidelines.xhtml

		Derivation			Validation		
**Risk Factor cont.**		**No of incident cases**	**Total Person years**	**Crude Incidence Rate per 10000 person years (95% CI)**	**No of incident cases**	**Total Person years**	**Crude Incidence Rate per 10000 person years (95% CI)**
Secondary Osteoporosis	No	262	7846.5	333.9 (295.83 to 376.89)	122	3853.4	316.60 (265.12 to 378.07)
	Yes	54	1506.2	358.51 (274.58 to 468.10)	30	775.4	386.89 (270.51 to 553.34)
Previous Fracture	No	144	6832.0	210.77 (179.01 to 248.17)	63	3319.4	189.79 (148.26 to 242.95)
	Yes	172	2520.8	682.32 (587.61 to 792.31)	89	1309.4	679.69 (552.18 to 836.64)
Previous Fracture, detail	None	144	6832.0	210.77 (179.01 to 248.17)	63	3319.4	189.79 (148.26 to 242.95)
	1 fracture	105	1919.6	546.99 (451.76 to 662.29)	52	1049.8	495.36 (377.47 to 650.07)
	2-4 fractures	57	557.9	1021.62 (788.04 to 1324.45)	33	236.2	1397.28 (993.37 to 1965.44)
	5+ fractures	10	43.2	2311.27 (1243.59 to 4295.60)	4	23.5	1701.81 (638.72 to 4534.31)
**Total**		**316**	**9352.8**	**337.87 (302.60 to 377.25)**	**152**	**4628.8**	**328.38 (280.11 to 384.96)**

For peer review only

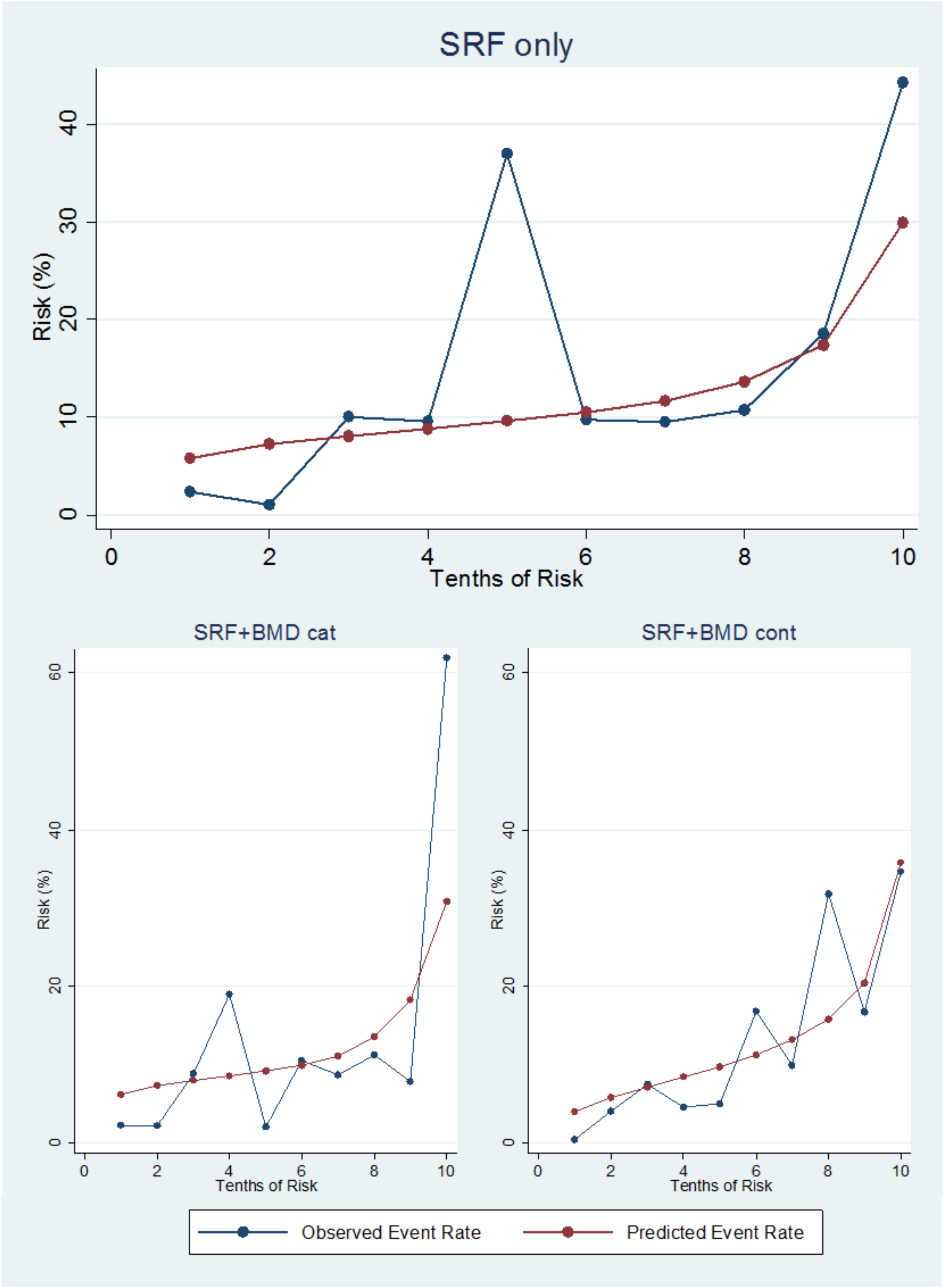

Supplementary Figure 1 Predicted and Observed risk by 10th of predicted risk for each risk prediction model in the derivation dataset.

TRIPOD Checklist: Prediction Model Development and Validation

Section/Topic	Item		Checklist Item	Page
**Title and abstract**				
Title	1	D;V	Identify the study as developing and/or validating a multivariable prediction model, the target population, and the outcome to be predicted.	1
Abstract	2	D;V	Provide a summary of objectives, study design, setting, participants, sample size, predictors, outcome, statistical analysis, results, and conclusions.	3
**Introduction**				
Background and objectives	3a	D;V	Explain the medical context (including whether diagnostic or prognostic) and rationale for developing or validating the multivariable prediction model, including references to existing models.	5
	3b	D;V	Specify the objectives, including whether the study describes the development or validation of the model or both.	5
**Methods**				
Source of data	4a	D;V	Describe the study design or source of data (e.g., randomized trial, cohort, or registry data), separately for the development and validation data sets, if applicable.	6
	4b	D;V	Specify the key study dates, including start of accrual; end of accrual; and, if applicable, end of follow-up.	6
Participants	5a	D;V	Specify key elements of the study setting (e.g., primary care, secondary care, general population) including number and location of centres.	6
	5b	D;V	Describe eligibility criteria for participants.	6
	5c	D;V	Give details of treatments received, if relevant.	-
Outcome	6a	D;V	Clearly define the outcome that is predicted by the prediction model, including how and when assessed.	6
	6b	D;V	Report any actions to blind assessment of the outcome to be predicted.	-
Predictors	7a	D;V	Clearly define all predictors used in developing or validating the multivariable prediction model, including how and when they were measured.	6/7
	7b	D;V	Report any actions to blind assessment of predictors for the outcome and other predictors.	-
Sample size	8	D;V	Explain how the study size was arrived at.	-
Missing data	9	D;V	Describe how missing data were handled (e.g., complete-case analysis, single imputation, multiple imputation) with details of any imputation method.	7
Statistical analysis methods	10a	D	Describe how predictors were handled in the analyses.	6/7
	10b	D	Specify type of model, all model-building procedures (including any predictor selection), and method for internal validation.	7
	10c	V	For validation, describe how the predictions were calculated.	6/7
	10d	D;V	Specify all measures used to assess model performance and, if relevant, to compare multiple models.	7/8
	10e	V	Describe any model updating (e.g., recalibration) arising from the validation, if done.	7
Risk groups	11	D;V	Provide details on how risk groups were created, if done.	8
Development vs. validation	12	V	For validation, identify any differences from the development data in setting, eligibility criteria, outcome, and predictors.	7
**Results**				
Participants	13a	D;V	Describe the flow of participants through the study, including the number of participants with and without the outcome and, if applicable, a summary of the follow-up time. A diagram may be helpful.	9
	13b	D;V	Describe the characteristics of the participants (basic demographics, clinical features, available predictors), including the number of participants with missing data for predictors and outcome.	9
	13c	V	For validation, show a comparison with the development data of the distribution of important variables (demographics, predictors and outcome).	10/11
Model development	14a	D	Specify the number of participants and outcome events in each analysis.	9
	14b	D	If done, report the unadjusted association between each candidate predictor and outcome.	11
Model specification	15a	D	Present the full prediction model to allow predictions for individuals (i.e., all regression coefficients, and model intercept or baseline survival at a given time point).	Suppl doc
	15b	D	Explain how to the use the prediction model.	Suppl doc
Model performance	16	D;V	Report performance measures (with CIs) for the prediction model.	13/14
Model-updating	17	V	If done, report the results from any model updating (i.e., model specification, model performance).	13/14
**Discussion**				
Limitations	18	D;V	Discuss any limitations of the study (such as nonrepresentative sample, few events per predictor, missing data).	15/16
Interpretation	19a	V	For validation, discuss the results with reference to performance in the development data, and any other validation data.	14
	19b	D;V	Give an overall interpretation of the results, considering objectives, limitations, results from similar studies, and other relevant evidence.	15/16
Implications	20	D;V	Discuss the potential clinical use of the model and implications for future research.	16
**Other information**				
Supplementary information	21	D;V	Provide information about the availability of supplementary resources, such as study protocol, Web calculator, and data sets.	Suppl doc
Funding	22	D;V	Give the source of funding and the role of the funders for the present study.	2

*Items relevant only to the development of a prediction model are denoted by D, items relating solely to a validation of a prediction model are denoted by V, and items relating to both are denoted D;V. We recommend using the TRIPOD Checklist in conjunction with the TRIPOD Explanation and Elaboration document.

For peer review only - http://bmjopen.bmj.com/site/about/guidelines.xhtml

BMJ Open

Does Bone Mineral Density Improve the Predictive Accuracy of Fracture Risk Assessment? A Prospective Cohort Study in Northern Denmark.

Journal:	*BMJ Open*
Manuscript ID	bmjopen-2017-018898.R2
Article Type:	Research
Date Submitted by the Author:	05-Feb-2018
Complete List of Authors:	Dhiman, Paula; University of Nottingham, School of Medicine<br>Andersen, Stig; Aalborg Universitetshospital, Department of Clinical Medicine<br>Vestergaard, Peter; Aalborg Universitetshospital, Department of Clinical Medicine and Endocrinology<br>Masud, Tahir ; University of Nottingham School of Medicine<br>Qureshi, Nadeem; University of Nottingham, Division of Primary Care
**Primary Subject Heading**:	General practice / Family practice
Secondary Subject Heading:	Research methods, Geriatric medicine, Public health
Keywords:	risk prediction, osteoporosis, fracture, predictive accuracy, bone mineral density

SCHOLARONE™ Manuscripts

only

Title

Does Bone Mineral Density Improve the Predictive Accuracy of Fracture Risk Assessment? A Prospective Cohort Study in Northern Denmark.

Authors list (names, affiliations, and position)

Paula Dhiman, Stig Andersen, Peter Vestergaard, Tahir Masud, Nadeem Qureshi

Division of Primary Care, University of Nottingham, Nottingham, NG7 2RD, UK

Paula Dhiman (Research Fellow)

Geriatric Medicine, Department of Clinical Medicine, Aalborg University Hospital, Hobrovej 18-22 Aalborg University Hospital, 9000 Aalborg, Denmark

Stig Andersen (Clinical Professor)

Department of Clinical Medicine and Endocrinology, Aalborg University Hospital Mølleparkvej 4, DK-9000 Aalborg, Denmark

Peter Vestergaard (Clinical Professor)

Geriatric Medicine, South Block, B Floor, Queens Medical Centre Campus, University of Nottingham, Nottingham NG7 2UH, UK

Tahir Masud (Honorary Professor)

Division of Primary Care, University of Nottingham, Nottingham, NG7 2RD, UK

Nadeem Qureshi (Clinical Professor)

Correspondence to: P Dhiman paula.dhiman@ndorms.ox.ac.uk

Copyright

The Corresponding Author has the right to grant on behalf of all authors and does grant on behalf of all authors, a worldwide license to the Publishers and its licensees in perpetuity, in all forms, formats and media (whether known now or created in the future), to i) publish, reproduce, distribute, display and store the Contribution, ii) translate the Contribution into other languages, create adaptations, reprints, include within collections and create summaries, extracts and/or, abstracts of the Contribution, iii) create any other derivative work(s) based on the Contribution, iv) to exploit all subsidiary rights in the Contribution, v) the inclusion of electronic links from the Contribution to third party material where-ever it may be located; and, vi) license any third party to do any or all of the above.

Exclusive License Statement

The Corresponding Author has the right to grant on behalf of all authors and does grant on behalf of all authors, an exclusive licence (or non exclusive for government employees) on a worldwide basis to the BMJ Publishing Group Ltd to permit this article (if accepted) to be published in BMJ editions and any other BMJPGL products and sublicenses such use and exploit all subsidiary rights, as set out in our licence.

For peer review only - http://bmjopen.bmj.com/site/about/guidelines.xhtml

Competing Interests

We have read and understood BMJ policy on declaration of interests and declare that we have no competing interests.

All authors have completed the ICMJE uniform disclosure form at www.icmje.org/coi_disclosure.pdf and declare: no support from any organisation for the submitted work; no financial relationships with any organisations that might have an interest in the submitted work in the previous three years; no other relationships or activities that could appear to have influenced the submitted work.

Transparency declaration

The lead author (Dr Dhiman) affirms that this manuscript is an honest, accurate, and transparent account of the study being reported; that no important aspects of the study have been omitted; and that any discrepancies from the study as planned (and, if relevant, registered) have been explained.

Ethical Approval

Ethics approval was given through the Region of North Jutland's from the Danish Data Protection Agency ("paraplyanmeldelse 2008-58-0028").

Funding

This study was funded through the National Institute for Health Research (NIHR), School for Primary Care Research (SPCR).

NIHR acknowledgement

This paper presents independent research funded by the National Institute for Health Research School for Primary Care Research (NIHR SPCR).

Disclaimer

The views expressed are those of the author(s) and not necessarily those of the NIHR, the NHS or the Department of Health.

Patient Involvement

Patients were not involved in the development of this research question.

Data Sharing Statement

Data sharing: Technical appendix and statistical code is available from the corresponding author at paula.dhiman@nottingham.ac.uk. Due to restrictions by the Danish Data Protection Agency, data can only be shared on an aggregated level and by special permission.

Contributorship

Contributors: PD wrote the statistical analysis plan, cleaned and analysed the data, and drafted and revised the paper. PV and SA provided the AURORA dataset for analysis and linked patients to the National Patient Registry of Denmark, they also reviewed and revised the draft paper. NQ and TM provided clinical expertise, and reviewed and revised the draft paper.

For peer review only - http://bmjopen.bmj.com/site/about/guidelines.xhtml

Abstract:

Objective: To evaluate the added predictive accuracy of bone mineral density (BMD) to fracture risk assessment.

Design: Prospective cohort study using data between 01/01/2010 and 31/12/2012.

Setting: North Denmark Osteoporosis Clinic of referred patients presenting with at least one fracture risk factor to the referring doctor.

Participants: Patients aged 40-90 years; had BMD T-score recorded at the hip; and not taking osteoporotic preventing drugs for more than 1-year prior to baseline.

Main outcome measures: Incident diagnoses of osteoporotic fractures (hip, spine, forearm, humerus, and pelvis) were identified using the National Patient Registry of Denmark during 01/01/2012-01/01/2014. Cox regression was used to develop a fracture model based on predictors in the Fracture Risk Assessment Tool (FRAX®), with and without, binary and continuous BMD. Change in Harrell's C-Index and Reclassification tables were used to describe the added statistical value of BMD.

Results: Adjusting for predictors included in FRAX®, osteoporotic patients (T-score≤-2.5) had 75% higher hazard of a fracture compared to patients with higher BMD (HR:1.75 (95% CI:1.28 to 2.38)). Forty-percent lower hazard was found per unit increase in continuous BMD T-score (HR:0.60 (95% CI:0.52 to 0.69)).

Accuracy improved marginally, and Harrell's C-Index increased by 1.2% when adding continuous BMD (0.76 to 0.77). Reclassification tables showed continuous BMD shifted 529 patients into different risk categories; 292 of these were reclassified correctly (57%; 95% CI:55% to 64%). Adding binary BMD however no improvement: Harrell's C-Index decreased by 0.6%.

Conclusions: Continuous bone mineral density marginally improves fracture risk assessment. Importantly, this was only found when using continuous BMD measurement for osteoporosis. It is suggested that future focus should be on evaluation of this risk factor using routinely collected data, and on the development of more clinically relevant methodology to assess the added value of a new risk factor

For peer review only

For peer review only - http://bmjopen.bmj.com/site/about/guidelines.xhtml

Article Summary

Strengths and Limitations:

- Addresses a research question recommended by The National Institute for Health and Care Excellence to investigate the added value of bone mineral density to fracture risk prediction.
- Investigates bone mineral density in both the commonly used, binary, and continuous format.
- Presents changes in calibration, discrimination, and reclassification to describe the added value of bone mineral density.
- Uses robustly collected data from Northern Denmark, with 3.2% missing data.
- As data is from a North Danish population, with at least one fracture risk factor, this limits generalisability of the results.

For peer review only

Introduction

Osteoporosis causes over 8.9 million fractures worldwide, of which over 4.5 million occur in the USA and Europe, and account for 2.8 million disability adjusted life years (1). Further, 1.2 million disability adjusted life years are accounted for by hip fractures, which are projected to increase to 6 million by 2050 (2).

Given this burden, and treatment options for osteoporosis, identifying patients at risk of an osteoporotic fracture is high priority amongst health policymakers to reduce the risk of future fracture (3). Risk prediction tools have been developed to aid in the identification of patients at risk. For example, the Fracture Risk Assessment Tool (FRAX®) and QFracture® are commonly used to assess fracture risk in patients based on pre-defined risk factors.

Bone mineral density (BMD), a measurement used to aid diagnosis of osteoporosis, has also been identified as a fracture risk factor (4-7) . Unlike some other fracture risk factors, treatment options (e.g. bisphosphonate medication) are available that reduces the fracture risk markedly when treatment is initiated based on low BMD.

English National guidelines (The National Institute for Health and Care Excellence (NICE)) for fracture risk assessment recommend treatment of osteoporosis to prevent fractures but have not included BMD as a mandatory risk factor for fracture risk prediction tools to incorporate (8). This is partly due to the lack of robust evidence and limited generalisability of current research, which has particularly focused on evaluating BMD in postmenopausal women evaluating the added value of BMD to existing fracture risk factors (5-7).

The National Institute of Clinical Health and Excellence also recognise this gap in the evidence and have recommended research to assess the added value of BMD as a risk factor in fracture risk assessment (9).

The aim of this study is to assess the value of BMD measurement in addition to the standard fracture risk factors used in the FRAX® risk model using a robustly collected prospective cohort.

For peer review only

Methods

This paper has been written in accordance to the TRIPOD checklist.

Patient Involvement

Patients were not involved in the development of this research question and were not involved in the design of this study.

Study Design and Data Source

A prospective cohort study was conducted using patients from the Aalborg University Hospital Record for Osteoporosis Risk Assessment (AURORA) dataset; patients were followed up using the National Patient Registry of Denmark.

The AURORA dataset consists of patients attending the Osteoporosis Clinic at Aalborg University Hospital after a referral from their primary care physician. A referral was offered to patients with at least one risk factor for osteoporosis (low BMI, previous fracture, parental hip fracture, smoking status, alcohol consumption, glucocorticoid use, rheumatoid arthritis, and secondary osteoporosis) or if they were aged 80 years and above. Further detail of the data collection has been described elsewhere (10). The Danish National Patient Registry which collects inpatient and outpatient data from all Danish hospitals, was linked to the AURORA dataset through unique patient identifiers

Ethics approval was given through the Region of North Jutland's from the Danish Data Protection Agency ("paraplyanmeldelse 2008-58-0028").

Cohort selection

Data collection for AURORA began 1st January 2010 and was collected for 3 years (up to 31st December 2012). Patients were included if they were aged 40-90 years; had a BMD T-score at the hip; and were not taking any osteoporotic preventing drugs or any bone sparing drugs for more than one year prior to baseline.

Primary Outcome

The primary outcome measure was an incident osteoporotic fracture during follow up (01/01/2012 to 01/01/2014); defined as a diagnosis of a fracture at the hip, spine, forearm, humerus, and pelvis. Fractures at these sites resulting from traffic, work, and sports related accidents were excluded from the study. Relevant fractures were identified in the Danish National Patient Registry, using the International Statistical Classification of Diseases, 10th Version codes (ICD-10 codes), which was developed using recognised database methodology for each fracture (11).

Fracture risk factors

Fracture risk factors, used in the FRAX® risk prediction model, were extracted at baseline. They were: age; gender; height (m); weight (kg); previous fracture; parental history of hip fracture, current smoking status; current alcohol consumption; glucocorticoid use (currently exposed for 3+ months); rheumatoid arthritis; and secondary osteoporosis (includes type I diabetes; osteogenesis imperfecta in adults; untreated, long standing hyperthyroidism;

For peer review only - http://bmjopen.bmj.com/site/about/guidelines.xhtml

hypogonadism; premature menopause (<45 years); chronic malnutrition; malabsorption; and chronic liver disease).

Bone Mineral Density

DXA scans were performed by trained technicians using Hologic Discovery A (Bedford, MA, USA). A daily QC programme was in place and in vivo CV using repositioning of patients was <1%. Total hip BMD was used as region of interest. Bone mineral density was added to the fracture risk prediction model twice, firstly, as a continuously measured T-score value, and secondly, as a binary risk factor, dichotomised at/above T-score threshold for osteoporosis and below threshold, -2.5 in T-score (manufacturers' normal range using normal material from T Kelly et al (12)) based on World Health Organisation (WHO) classifications (13). Calculated T-scores were gender specific.

Statistical Analysis

A complete case analysis was performed on the data; 3.2% of data was missing. The AURORA dataset was split into two using recognised methodology (14); where a random number was assigned to patients and based on a cut off, two-thirds was used to derive the risk models, and the remaining third was used to validate them.

Model derivation

Three Cox proportional hazards models were developed for the primary outcome, using a complete case analysis on the derivation dataset:

Model 1. Standard fracture risk factors only (without BMD)
Model 2. Standard fracture risk factors (with binary BMD)
Model 3. Standard fracture risk factors (with continuous BMD)

Graphical methods were used (log-log plots) to assess the proportional hazards assumption, and risk factors violating this assumption were added to the model as a time varying covariate.

Recognised methodology used in research studies was used to build the 3 risk prediction models (15, 16); the Kaplan Meier method was used to obtain 4-year fracture risk estimates for patients. Further detail on the conversion of the Cox proportional hazards models to risk prediction models has been provided in Supplementary Table 1.

Validation of Models

Four-year fracture risk was calculated from each model and the predictive performance of each risk prediction model was assessed by measures describing calibration, discrimination, and reclassification. These metrics were assessed using the validation cohort.

Calibration measures how well the predicted risk agrees with observed risk in the data. It plots the mean predicted and observed risk of fracture for each decile of predicted risk. The observed risk of fracture was derived from the 4 year Kaplan-Meier estimate. Good calibration indicates the predicted risk is close to the observed risk of the outcome.

Discrimination measures how well the risk prediction model differentiates between patients who have or have not observed the event in the study. This was quantified by the area under

For peer review only - http://bmjopen.bmj.com/site/about/guidelines.xhtml

the receiver operating characteristic (ROC) curve (AUC), given by Harrell's C-Index with higher values indicating better discrimination.

Reclassification tables (17) measures movement between risk categories when adding a new risk factor. Threshold for treatment at 4 years was set at a fracture risk level of 8.5%; to be comparable to the treatment threshold of 20% at 10 years. This was presented by the total percent of patients reclassified (incorrectly and correctly), and also the Net Reclassification Index (NRI) (18, 19). The NRI gives the net calculation of the changes in the right direction and a higher NRI indicates a better reclassifying model.

All analyses were carried out using Stata (version 12) (20).

For peer review only

For peer review only - http://bmjopen.bmj.com/site/about/guidelines.xhtml

Results

Characteristics of the data

The AURORA collected data on 7,912 patients; 1,795 patients were excluded comprising, 440 not aged between 40-90 years at baseline; 156 not having a recorded T-score value for the total hip at baseline; and 1,199 patients were taking anti-osteoporotic drug therapy for more than one year prior to baseline.

The study sample consisted of 6,117 patients; predominantly female (79.6%), and patients with a mean age of 62.9 (SD: 10.9) years. Two-thirds of this sample (n=4,093) was used for the derivation dataset and one-third (n=2,094) was used for the validation dataset. Table 1 presents the baseline characteristics of the study by derivation and validation dataset, and shows little difference between the datasets.

Patients in the derivation dataset had a median follow up time of 2.30 years [1.57, 2.99], and observed 318 (7.8%) osteoporotic fractures during follow up. Of these, 316 fractures were eligible for the analysis (2 patients had a fractures on or prior to baseline and were excluded). Patients contributed 9352.8 person years of observation, giving a total incidence rate of 337.87 per 10,000 person years (95% CI:302.60 to 377.25).

Fractures during follow up were predominantly found in the forearm (27.0%) and hip (17.9%). Higher fracture incidence rates were found in patients classed as osteoporotic, based on their T-score at both the total hip (809.73 per 10,000 person years (95% CI:641.68 to 1021.78)) and spine (L1-L4) (553.59 per 10,000 person years (95% CI:462.55 to 662.55)) (Supplementary Table 2).

For peer review only - http://bmjopen.bmj.com/site/about/guidelines.xhtml

Table 1. Baseline characteristics of the derivation and validation datasets, including missing data.

Characteristic			Derivation (n=4,093) No	Derivation %	Validation (n=2,024) No	Validation %
Gender	Female		3,266	79.8	1,602	79.2
	Male		827	20.2	422	20.8
Osteoporotic (Hip DXA)	No		3,683	90.0	1,820	89.9
	Yes		410	10.0	204	10.1
Osteoporotic Status (based on UK guidelines)	Normal		1,886	46.1	927	45.8
	Osteopenic		1,797	43.9	893	44.1
	Osteoporotic		410	10.0	204	10.1
Previous Fracture	No		2,935	71.7	1,423	70.3
	Yes		1,158	28.3	601	29.7
No of Previous Fractures	None		2,935	71.7	1,423	70.3
	1 fracture		862	21.1	467	23.1
	2-4 fractures		270	6.6	122	6.0
	5+ fractures		26	0.6	12	0.6
Parental History of Hip Fracture	No		2,755	67.3	1,359	67.1
	Yes		1,338	32.7	665	32.9
Current Smoking Status	other (non/ex)		3,182	77.7	1,529	75.5
	smoker		911	22.3	495	24.5
Alcohol Consumption	≤3 units per day		3,875	94.7	1,923	95.0
	>3 units per day		218	5.3	101	5.0
Glucocorticoid Use	No		3,577	87.4	1,741	86.0
	Yes		516	12.6	283	14.0
Rheumatoid Arthritis	No		3,686	90.1	1,801	88.0
	Yes		407	9.9	223	11.0
Other Bone Affecting Disease	No		2,382	58.2	1,139	56.3
	Yes		1,711	41.8	885	43.7
Secondary Osteoporosis	No		3,438	84.0	1,689	83.5
	Yes		655	16.0	335	16.6
	By disease					
	Type 1 diabetes	No	4,010	98.0	1,981	97.9
		Yes	83	2.0	43	2.1
	Osteogenesis	No	4,093	100	2,024	100
		Yes	0	0	0	0
	Hyperthyroidism	No	4,089	99.9	2,023	99.9
		Yes	4	0.1	1	0.1
	Malnutrition	No	4,090	99.9	2,023	99.9
		Yes	3	0.1	1	0.1
	Chronic Liver Disease	No	4,006	97.9	1,979	97.8
		Yes	87	2.1	45	2.2
	Menopause (Females only)**	No	853	26.1	405	25.3
		Yes	2,413	73.9	1,197	74.7

For peer review only - http://bmjopen.bmj.com/site/about/guidelines.xhtml

	Premature Menopause (<45years)***	No	1,904	78.9	941	78.6
		Yes	509	21.1	256	21.4
			**Mean**	**SD**	**Mean**	**SD**
Age (years)			62.9	10.9	63.0	11.0
Weight (kg)			72.1	15.5	72.2	15.9
	Missing		47	1.2	12	0.6
Height (m)			1.7	0.1	1.7	0.1
	Missing		131	3.2	61	3.0
BMI			26.4	5.0	26.4	5.1
	Missing		135	3.3	63	3.1
Hip DXA T-score			-1.1	1.1	-1.2	1.1

***out of patients with a fracture**
****proportion out of respective number of females**
*****proportion out of respective number of females with menopause**

Model development

The unadjusted analysis showed statistically significant association between BMD (continuous and binary) and osteoporotic fracture (HR=0.55; 95% CI: 0.50 to 0.61, p<0.001, HR=2.79; 95% CI: 2.11 to 3.67, p<0.001, respectively). Significant associations with fracture were also found with age (HR=1.03; 95% CI: 1.02 to 1.04, p<0.001), previous fracture (HR=3.38; 95% CI: 2.69 to 4.24, p<0.001), BMI (HR=0.97; 95% CI: 0.95 to 1.00, p=0.03), and gender (HR=0.73; 95% CI: 0.53 to 1.00, p=0.05). Further, a time-varying effect was found in patients with a previous fracture; hazard of a subsequent fracture was highest in the first year during follow up and decreased per year of follow up (p<0.001).

The adjusted analysis is presented in Table 2. Model 1 showed that of the standard risk factors, age and previous fracture were significantly associated with fracture; hazard of fracture increased by 2% per year increase in age (HR=1.02; 95% CI: 1.01 to 1.04); and increased almost 5 fold in patients with a previous fracture at time 0 years (HR=4.88; 95% CI: 3.37 to 7.08).

Hazard of fracture increased by 75% for patients classed as osteoporotic by their BMD score (Model 2, HR=1.75; 95% CI: 1.28 to 2.38). Hazard of fracture also decreased by 40% per SD improvement in BMD T-score (Model 3, HR=0.60; 95% CI: 0.52 to 0.69).

For peer review only

Table 2. Multivariable analysis for osteoporotic fracture in the derivation cohort. Data are adjusted hazard ratios and 95% confidence intervals.

Risk Factor		Adjusted Hazard Ratio (95% CI)		
		**Model 1: Standard Risk Factors only**	**Model 2: Standard Risk Factors + BMD (categorical)**	**Model 3: Standard Risk Factors + BMD (continuous)**
Age (years)		1.024 (1.013 to 1.036)	1.019 (1.007 to 1.031)	1.007 (0.995 to 1.019)
Gender	Female	Ref	Ref	Ref
	Male	0.754 (0.544 to 1.044)	0.796 (0.573 to 1.104)	0.851 (0.613 to 1.181)
BMI		0.978 (0.954 to 1.002)	0.989 (0.965 to 1.013)	1.027 (1.000 to 1.055)
Previous Fracture	No	Ref	Ref	Ref
	Yes (time = 0 years)	4.88 (3.336 to 7.078)	4.667 (3.214 to 6.778)	4.018 (2.763 to 5.842)
Parental History of Hip Fracture	No	Ref	Ref	Ref
	Yes	1.079 (0.834 to 1.397)	1.096 (0.847 to 1.419)	1.105 (0.854 to 1.430)
Current Smoker	No	Ref	Ref	Ref
	Yes	1.121 (0.852 to 1.475)	1.076 (0.817 to 1.417)	1.019 (0.774 to 1.342)
Alcohol Consumption (>3 units/day)	No	Ref	Ref	Ref
	Yes	1.414 (0.904 to 2.212)	1.440 (0.921 to 2.252)	1.459 (0.932 to 2.283)
Glucocorticoid Use	No	Ref	Ref	Ref
	Yes	1.080 (0.753 to 1.550)	1.052 (0.733 to 1.510)	1.038 (0.724 to 1.489)
Rheumatoid Arthritis	No	Ref	Ref	Ref
	Yes	1.098 (0.731 to 1.650)	1.089 (0.725 to 1.637)	1.116 (0.742 to 1.678)
Secondary Osteoporosis	No	Ref	Ref	Ref
	Yes	0.993 (0.729 to 1.354)	0.966 (0.708 to 1.317)	0.911 (0.667 to 1.243)
Osteoporotic	No	Ref	Ref	Ref
	Yes	-	1.745 (1.279 to 2.381)	-
Hip DXA T-score (SD)		-	-	0.600 (0.524 to 0.686)
Previous Fracture (TVC*)		0.635 (0.489 to 0.826)	0.639 (0.492 to 0.830)	0.644 (0.495 to 0.837)

*TVC = time varying covariate, value is interaction effect and 95% CI.

For peer review only - http://bmjopen.bmj.com/site/about/guidelines.xhtml

Model Validation

The 4-year predicted risk of fracture was calculated for all patients in the validation dataset; this was compared to the observed fracture outcome within the 4 year follow up.

Calibration and Discrimination

Calibration plots suggested some improvement when adding BMD measurement; particularly when including continuous BMD T-score measurement (Model 3; Supplementary Figure 1).

The largest change in discrimination was found when adding continuous BMD measurement to standard risk factors; Harrell's C-Index increased by 1.17% (Table 3). However, binary BMD measurement, as a measure for osteoporotic patients, was found to reduce Harrell's C-Index by -0.65%.

Table 3. Harrell's C-Index for Model 1, 2, 3, 4, and 5.

Model	Harrell's C-Index	Change in Harrell's C-Index (% change)*
**Model 1**: Standard fracture risk factors only (without BMD)	0.764 (0.718 to 0.810)	-
**Model 2**: Standard fracture risk factors only (with binary BMD)	0.759 (0.712 to 0.806)	-0.005 (-0.65%)
**Model 3**: Standard fracture risk factors only (with continuous BMD)	0.773 (0.732 to 0.814)	0.009 (1.17%)

*All change is measures against Model 1.

Reclassification

Reclassification tables indicated that adding continuous BMD measurement improved classification of patients into their correct risk categories. This was not found when adding binary BMD. Table 4 presents the reclassification table for Model 1 (standard fracture risk factors only) and Model 3 (standard risk factors with continuous BMD), using the 8.5% pre-specified risk threshold.

For peer review only - http://bmjopen.bmj.com/site/about/guidelines.xhtml

Table 4. Risk Reclassification Table comparing Model 1 (standard fracture risk factors alone) to Model 3 (standard fracture risk factors with continuous BMD measurement), using a clinical 8.5% risk cut off.

			**Model 3: SRF with continuous BMD**			
			<8.5%	≥8.5%	Total	Total No.(%) Reclassified
**Model 1: SRF without BMD**	<8.5%	No	391	227	618	227 (36.7%)
		%	63.3%	36.7%		
		No. Events	5	9		
		No. Non events	386	218		
		Observed Event Rate	1.3%	4.0%		
	≥8.5%	No	302	1,040	1,342	302 (22.5%)
		%	22.5%	77.5%		
		No. Events	10	121		
		No. Non events	292	919		
		Observed Event Rate	3.3%	11.6%		
	Total		693	1,267	1,960	529 (27.0%)

Of the 1,960 patients in the validation dataset, 27% (n=529) were reclassified into a different risk category when including continuous BMD into fracture risk prediction. Two percent (9/529) were found to be reclassified correctly into a higher risk group and 55% (292/529) were reclassified correctly into a lower risk group; indicating 22% (292/1342) of patients at high risk in Model 1, not accounting for BMD measurement, were no longer at high risk. The net reclassification improvement when adding continuous BMD to standard risk factors, was 0.03, which resulted from increased specificity (non-event NRI = 4%) and decreased sensitivity (event NRI: -1%) from Model 1 (Table 5).

Table 5. Summary of Net Reclassification Index (NRI) and Integrated Discrimination Index (IDI) for all comparisons between developed fracture risk prediction models.

Comparison	Event NRI	Non-Event NRI	Overall NRI
Model 1 vs. Model 2	-3.45%	2.09%	-0.01
Model 1 vs. Model 3	-0.69%	4.08%	0.03

For peer review only - http://bmjopen.bmj.com/site/about/guidelines.xhtml

Discussion

Summary of Findings

Bone mineral density showed significant association with fracture risk with a 40% decrease for each SD rise in BMD. However, this resulted in small improvements in calibration, discrimination, and reclassification. The c-index estimate was slightly higher with continuous BMD but this increase is not conclusive given the width of the confidence intervals. Despite the limited improvement found of 1% in discrimination when adding continuous BMD, reclassification tables showed 57% of reclassified patients moving into their correct risk group through improved specificity. Importantly, no improvement was found when adding BMD in a binary format.

Our findings are consistent with and corroborate with current literature (7, 21, 22). Specifically, a study conducted in the Netherlands with 4 year follow up, investigating the added value of BMD for hip fractures risk, found modest improvement in predictability (21). Further, two more recent studies also indicated limited added value of BMD to fracture risk prediction (7, 22).

Strengths and Limitations

Answering Evidence gap

To our knowledge, this is the first study to investigate the added value of BMD in a binary and continuous format, to standard fracture risk factors. Further, it is based on a larger sample size than other studies investigating BMD in addition to FRAX (7, 21, 22). It helps inform the NICE research recommendation to assess the added value of BMD to routine fracture risk assessment in primary care (23). It further highlights that the more commonly used for treatment decision making, binary format of BMD resulted in a loss of predictability in fracture risk prediction; based on comparable measures for discrimination and reclassification

Robustness of Data

The prospective cohort was well populated with key standard risk factors recorded: BMI, smoking status and alcohol consumption, and personal and parental fracture history. Other than 3.2% of missing data for BMI, in 6,117 patients, complete data was collected for all risk factors (including BMD T-score recorded at the total hip). Further, the cohort was linked to a national robust electronic health records. This Danish National Patient Registry allowed for outcome fracture to be identified and also provided data on the mechanism for the fracture; this helped more accurately phenotype osteoporotic fractures.

Generalisability

The generalisability is affected in a few ways. Firstly, the findings are based on a Danish cohort. Secondly, AURORA data was collected from patients who presented to their doctor with at least one fracture risk factor and were referred to the osteoporosis clinic; this led to a biased study sample with a higher risk of a fracture and increased age. This could overestimate fracture risk amongst patients in a primary care setting.

Methodology

For peer review only

Due to the increased age of the sample, death becomes a competing risk. However, information on death was not collected and could not be retrieved. This limited the analysis of the data as competing risks could not be accounted for which may again lead to an overestimation of fracture risk (24). However, as an independent study primarily assessing the added value of BMD through deriving and validating the fracture risk prediction models, this bias would be present in both analyses to compare derived risk models with and without BMD measurement.

The FRAX risk algorithm has not yet been published, therefore FRAX estimates could not be directly calculated for the cohort. Instead, the FRAX risk model was recalibrated on the dataset with and without BMD added. Further, fracture outcomes in this study included pelvic fractures which are increasingly recognised as low trauma fragility fractures [(25)], and used BMD taken at the total hip instead of at the femur neck as it is the gold standard in Denmark (26).

Internal validation was performed to validate the derived risk prediction models. This may lead to over optimistic results of the performance of the risk models (14). To account for this limitation, a commonly practised method which randomly assigns patients to the derivation and validation datasets was used; further, a similar 1:2 ratio was also used to split the data (27-29).

The study had a 4 year follow up which is shorter than other recognised risk models. To account for this, we adapted the 20% clinical risk threshold for 10 year fracture estimates to 8.5% for 4 year fracture estimates, assuming that risk is constant over time (30, 31).

Traditional methodology assessing the added value to risk factors to existing risk prediction models are criticised to be insensitive to change, to lack interpretability (32-35). This was shown when finding a 1% change in Harrell's C-Index and overlapping confidence intervals between models, limiting the interpretability of results. Reclassification analysis was thus also used to provide more clinically interpretable results.

Clinical implications

The most notable clinical implication is the more routine use of BMD measurement for fracture risk assessment. Further, evidence suggests continuous BMD adds better predictability compared to the binary format.

Future Research

Further research is recommended to evaluate the added value of BMD to fracture risk prediction; in particular in addition to QFracture risk factors and using primary care routinely collected data. However, a brief interrogation into the Clinical Practice Research Datalink, a routinely collected UK primary care database, showed poor availability of BMD measurement in patient records, and thus, strong limitations to potential analyses. Less than 1% of patients had BMD recorded from a sample of 60,658 patients aged 40-90; not on any osteoporotic treatment; and with complete data for age, gender, BMI, smoking status, and alcohol consumption. Thus, prior to UK analysis, BMD recording in primary care databases needs to improve.

For peer review only - http://bmjopen.bmj.com/site/about/guidelines.xhtml

Methodologically, as well as assessing the added value of BMD to standard risk factors, we should also explore the option to replace existing fracture risk factors with the BMD measurement; this has rarely been explored in the literature but should be considered in future analyses. We also recommend research to investigate the added value of BMD in a potentially more natural, 3 group format of BMD (osteopenic, normal, osteoporotic).

In addition, further research is recommended to develop current methodology used to assess the added value of BMD to provide more clinically relevant results, such as cost implications; and to allow for better comparability between new risk factors with respect to their added value, thus improving decision making.

Conclusion

Continuous BMD marginally improves fracture risk assessment. Importantly, this was only found when using continuous BMD measurement for osteoporosis. It seems that prediction models for fragility fracture risk may be improved only marginally, using present risk factor assessment and evaluations. It is suggested that future focus should be on additional risk factors and on the development of more clinically relevant methodology to assess the added value of a new risk factor.

For peer review only

For peer review only - http://bmjopen.bmj.com/site/about/guidelines.xhtml

References

1. Kanis JA on behalf of the World Health Organization Scientific Group (2007). Assessment of osteoporosis at the primary health-care level. Technical Report. World Health Organization Collaborating Centre for Metabolic Bone Diseases, University of Sheffield, UK; 2007.
2. WHO scientific group. Prevention and management of osteoporosis: report of a WHO scientific group. WHO Technical Report Series. 2003.
3. Barry P, Aspray T, Briers K, Collins GS, Compston J, Dockery F, et al. Osteoporosis: assessing the risk of fragility fracture. 2012. Contract No.: CG146.
4. Kanis JA, Borgstrom F, Laet CD, Johansson H, Johnell O, Jonsson B, et al. Assessment of fracture risk. Osteoporosis International. 2005;16(6):581-9.
5. Report of a WHO Study Group. Assessment of fracture risk and its application to screening for postmenopausal osteoporosis. 1994.
6. McCloskey E, Johansson H, Oden A, Kanis JA. Fracture risk assessment. Clinical biochemistry. 2012;45(12):887-93.
7. Briot K, Paternotte S, Kolta S, Eastell R, Felsenberg D, Reid DM, et al. FRAX: Prediction of Major Osteoporotic Fractures in Women from the General Population: The OPUS Study. PLoS ONE. 2013;8(12):e83436.
8. National Institute for Health and Care Excellence. Osteoporosis: assessing the risk of fragility fracture. Short clinical guideline - evidence and recommedation. London: National Clinical Guidance Centre. 2012.
9. National Institute for Health and Care Excellence. Osteoporosis: assessing the risk of fragility fracture. NICE short clinical guideline CG146.; 2012.
10. Andersen S, Laurberg P. Age discrimination in osteoporosis screening-data from the Aalborg University Hospital Record for Osteoporosis Risk Assessment (AURORA). Maturitas. 2014;77(4):330-5.
11. Dave S, Petersen I. Creating medical and drug code lists to identify cases in primary care databases. Pharmacoepidemiol Drug Saf. 2009;18(8):704-7.
12. Kelly TL. Bone mineral density reference databases for American men and women. Journal of Bone and Mineral Research. 1990;5(Suppl 2).
13. Kanis JA, Melton LJr, Christianson C, Johnston CC, Khaltaev N. The diagnosis of osteoporosis. Journal of Bone and Mineral Research. 1994;9(8):1137-41.
14. Moons KGM, Kengne AP, Woodward M, Royston P, Vergouwe Y, Altman DG, et al. Risk prediction models: I. Development, internal validation, and assessing the incremental value of a new (bio)marker. Heart. 2012.
15. Hippisley-Cox J, Coupland C. Derivation and validation of updated QFracture algorithm to predict risk of osteoporotic fracture in primary care in the United Kingdom: prospective open cohort study. Bmj. 2012;344:e3427.
16. Hippisley-Cox J, Coupland C, Vinogradova Y, Robson J, Minhas R, Sheikh A, et al. Predicting cardiovascular risk in England and Wales: prospective derivation and validation of QRISK2. British Medical Journal. 2008;336:1475-82.
17. Cook NR. Use and misuse of the receiver operating characteristic curve in risk prediction. Circulation. 2007;115(7):928-35.
18. Cook NR. Comments on 'Evaluating the added predictive ability of a new marker: From area under the ROC curve to reclassification and beyond' by M. J. Pencina et al., Statistics in Medicine. Statistics in Medicine. 2008;27(2):191-5.
19. Pencina MJ, D'Agostino RB, Sr., Steyerberg EW. Extensions of net reclassification improvement calculations to measure usefulness of new biomarkers. Statistics in Medicine. 2011;30(1):11-21.

For peer review only - http://bmjopen.bmj.com/site/about/guidelines.xhtml

20. StataCorp. Stata Statistical Software: Release 12. College Station, TX: StataCorp LP; 2011.
21. Burger H, de Laet CE, Weel AE, Hofman A, Pols HA. Added value of bone mineral density in hip fracture risk scores. Bone. 1999;25(3):369-74.
22. Marques A, Lucas R, Simoes E, Verstappen SMM, Jacobs JWG, da Silva JAP. Do we need bone mineral density to estimate osteoporotic fracture risk? A 10-year prospective multicentre validation study. RMD Open. 2017;3(2):e000509.
23. Barry P, Aspray T, Briers K, Collins GS, Compston J, Dockery F, et al. Osteoporosis: assessing the risk of fragility fracture (NICE Clinical Guidance 146). National Institute for Health and Care Excellence; 2012. Contract No.: CG146.
24. Satagopan JM, Ben-Porat L, Berwick M, Robson M, Kutler D, Auerbach AD. A note on competing risks in survival data analysis. Br J Cancer. 2004;91(7):1229-35.
25. Soles G, Ferguson T. Fragility fractures of the pelvis. Curr Rev Musculoskelet Med. 2012;5:222-8.
26. Abrahamsen B, Stilgren LS, Hermann AP, Tofteng CL, Barenholdt O, Vestergaard P, et al. Discordance between changes in bone mineral density measured at different skeletal sites in perimenopausal women--implications for assessment of bone loss and response to therapy: The Danish Osteoporosis Prevention Study. J Bone Miner Res. 2001;16(7):1212-9.
27. Hippisley-Cox J, Coupland C, Vinogradova Y, Robson J, May M, Brindle P. Derivation and validation of QRISK, a new cardiovascular disease risk score for the United Kingdom: prospective open cohort study. British Medical Journal. 2007;335(7611):136-41.
28. Ridker PM, Buring JE, Rifai N, Cook NR. Development and validation of improved algorithms for the assessment of global cardiovascular risk in women: the Reynolds Risk Score.[Erratum appears in JAMA. 2007 Apr 4;297(13):1433]. Jama. 2007;297(6):611-9.
29. Hippisley-Cox J, Coupland C, Robson J, Brindle P. Derivation, validation, and evaluation of a new QRISK model to estimate lifetime risk of cardiovascular disease: cohort study using QResearch database. British Medical Journal. 2010;341.
30. National Osteoporosis Foundation. Clinician's Guide to Prevention and Treatment of Osteoporosis. Washington, DC; 2013.
31. Thomsen K. Odense Fallers and Osteoporosis Study: Assessment of osteoporosis among older women presenting with falls. Denmark: Odense University Hospital; 2014.
32. McGeechan K, Macaskill P, Irwig L, Liew G, Wong TY. Assessing New Biomarkers and Predictive Models for Use in Clinical Practice A Clinician's Guide. Archives of Internal Medicine. 2008;168(21):2304-10.
33. Steyerberg EW, Pencina MJ, Lingsma HF, Kattan MW, Vickers AJ, Van Calster B. Assessing the incremental value of diagnostic and prognostic markers: a review and illustration. European Journal of Clinical Investigation. 2012;42(2):216-28.
34. Janes H, Pepe MS, Gu W. Assessing the Value of Risk Predictions by Using Risk Stratification Tables. Annals of Internal Medicine. 2008;149(10):751-W162.
35. Cook NR. Use and misuse of the receiver operating characteristic curve in risk prediction - Response. Circulation. 2007;116(6):E134-E.

For peer review only

For peer review only - http://bmjopen.bmj.com/site/about/guidelines.xhtml

Supplementary Information

Beta coefficients from each Cox regression model were used to create each fracture risk prediction model.

Once all 5 models were finalised, their beta coefficients were used to create 5 risk prediction models and calculate risk of fracture for each patient, using the following general equation:

$$\widehat{risk} = 1 - S_0(t)^{exp(\sum_{i=1}^{p} \beta_i X_i - \sum_{i=1}^{p} \beta_i \bar{X}_i)}$$

Where $\mathbf{S_0(t)}$ is the baseline survival rate at follow up time, **t**(for this example, a follow up time of 10 years will be used); beta ($\boldsymbol{\beta_i}$) are the regression coefficients for each included risk factor in the model (**i**); $\mathbf{X_i}$ is the observed data value for each risk factor; $\mathbf{\bar{X}_i}$ is the corresponding mean for each risk factor; and **p** is the total number of risk factors included in the model. Table A1 shows the formula for each risk prediction model explicitly.

For peer review only

Supplementary Table 1. Risk equations to calculate 4 year risk based on patient characteristics for each developed risk model.

**General Risk Equation to calculate 4 year risk**	$1 - 0.89^{exp(\sum_{i=1}^{p} \beta_i X_i - \sum_{i=1}^{p} \beta_i \bar{X}_i)}$
**Risk Model**	**Equation**
Model 1	where $\sum_{i=1}^{p} \beta_i X_i =$<br>0.0237745*age+-0.2826461*gender+-0.0225011*BMI+1.585278*previous fracture+<br>0.0762559*parental hip fracture+0.1138883*smoking status+0.0773898*glucocorticoid use+<br>0.3465287*alcohol consumption+0.0936966*rheumatoid arthritis+<br>-0.0069432*secondary osteoporosis+-0.4535108*(previous fracture*time)<br>where $\sum_{i=1}^{p} \beta_i \bar{X}_i =$<br>0.0237745*mean age+-0.2826461*mean gender+-0.0225011*mean BMI+<br>1.585278*mean previous fracture+0.0762559*mean parental hip fracture+<br>0.1138883*mean smoking status+0.0773898*mean glucocorticoid use+<br>0.3465287*mean alcohol consumption+0.0936966*mean rheumatoid arthritis+<br>-0.0069432*mean secondary osteoporosis+-0.4535108*mean (previous fracture*time)
Model 2	where $\sum_{i=1}^{p} \beta_i X_i =$<br>0.0186827*age+-0.228784*gender+-0.0113651*BMI+1.540559*previous fracture+<br>0.092011*parental hip fracture+0.0732564*smoking status+0.0508706*glucocorticoid use+<br>0.3649544*alcohol consumption+0.0854353*rheumatoid arthritis+ -0.0346885*secondary osteoporosis+0.5568944*osteoporosis+-0.4481145*(previous fracture*time)<br>where $\sum_{i=1}^{p} \beta_i \bar{X}_i =$<br>0.0186827*mean age+-0.228784*mean gender+-0.0113651*mean BMI+<br>1.540559*mean previous fracture+0.092011*mean parental hip fracture+<br>0.0732564*mean smoking status+0.0508706*mean glucocorticoid use+<br>0.3649544*mean alcohol consumption+0.0854353*mean rheumatoid arthritis+<br>-0.0346885*mean secondary osteoporosis+0.5568944*mean osteoporosis+<br>-0.4481145*mean (previous fracture*time)

[Table continues on the next page]

For peer review only

Risk Model cont.	Equation
Model 3	where $\sum_{i=1}^{p} \beta_i X_i =$<br>0.0071931*age+-0.1615582*gender+0.0268478*BMI+1.39069*previous fracture+<br>0.1000272*parental hip fracture+0.0192416*smoking status+0.0374944*glucocorticoid use+<br>0.3774416*alcohol consumption+0.1097646*rheumatoid arthritis+ -0.0932063*secondary<br>osteoporosis+-0.5110986*t-score+-0.4404955*(previous fracture*time)<br>where $\sum_{i=1}^{p} \beta_i \bar{X}_i =$<br>0.0071931*mean age+-0.1615582*mean gender+0.0268478*mean BMI+<br>1.39069*mean previous fracture+0.1000272*mean parental hip fracture+<br>0.0192416*mean smoking status+0.0374944*mean glucocorticoid use+<br>0.3774416*mean alcohol consumption+0.1097646*mean rheumatoid arthritis+<br>-0.0932063*mean secondary osteoporosis+-0.5110986*mean t-score+<br>-0.4404955*mean (previous fracture*time)

For peer review only

Supplementary Table 2. Crude fracture incidence rates for the derivation and validation datasets.

Risk Factor		Derivation			Validation		
		No of incident cases	Total Person years	Crude Incidence Rate per 10000 person years (95% CI)	No of incident cases	Total Person years	Crude Incidence Rate per 10000 person years (95% CI)
Age Category	40-49	17	1169.9	145.31 (90.33 to 233.74)	12	557.8	215.15 (122.19 to 378.84)
	50-59	70	2534.7	276.17 (218.49 to 349.07)	33	1311.0	251.71 (178.95 to 354.06)
	60-69	93	3062.9	303.63 (247.79 to 372.06)	42	1453.9	288.87 (213.48 to 390.88)
	70-79	83	1906.0	435.46 (351.17 to 539.98)	49	958.7	511.11 (386.29 to 676.26)
	80-89	52	652.7	796.75 (607.13 to 1045.59)	16	347.4	460.56 (282.15 to 751.77)
	90-99	1	26.6	376.51 (53.04 to 2672.85)	-	-	-
Osteoporotic - Hip	No	245	8475.9	289.05 (255.03 to 327.61)	123	4165.1	295.31 (247.48 to 352.40)
	Yes	71	876.8	809.73 (641.68 to 1021.78)	29	463.8	625.28 (434.52 to 899.78)
Osteoporotic - Spine	No	191	7025.8	271.86 (235.91 to 313.28)	111	3475.6	319.37 (265.16 to 384.67)
	Yes	119	2149.6	553.59 (462.55 to 662.55)	39	1089.1	358.08 (261.63 to 490.10)
Gender	Female	266	7417.5	358.61 (318.01 to 404.40)	129	3679.8	350.56 (295.00 to 416.59)
	Male	50	1935.3	258.36 (195.82 to 340.88)	23	949.0	242.36 (161.05 to 364.71)
Parental History Hip Fracture	No	220	6281.5	350.24 (306.88 to 399.71)	118	3108.7	379.58 (316.92 to 454.64)
	Yes	96	3071.3	312.57 (255.90 to 381.79)	34	1520.2	223.66 (159.81 to 313.02)

[Table continues on the next page]

For peer review only

Risk Factor cont.		Derivation: No of incident cases	Derivation: Total Person years	Derivation: Crude Incidence Rate per 10000 person years (95% CI)	Validation: No of incident cases	Validation: Total Person years	Validation: Crude Incidence Rate per 10000 person years (95% CI)
Current Smoker	No	240	7279.1	329.71 (290.53 to 374.18)	103	3513.1	293.19 (241.70 to 355.65)
	Yes	76	2073.7	366.5 (292.71 to 458.90)	49	1115.8	439.16 (331.91 to 581.07)
Alcohol Consumption more than 3 units per day	No	293	8875.9	330.11 (294.39 to 370.15)	140	4399.7	318.2 (269.63 to 375.53)
	Yes	23	476.9	482.33 (320.52 to 725.83)	12	229.2	523.66 (297.39 to 922.09)
Glucocorticoid Use (3 months)	No	279	8184.5	340.89 (303.15 to 383.33)	132	3993.1	330.57 (278.73 to 392.06)
	Yes	37	1168.3	316.7 (229.47 to 437.11)	20	635.8	314.57 (202.95 to 487.59)
Menopause	No	68	1962.8	346.44 (273.15 to 439.39)	29	928.9	312.19 (216.94 to 449.24)
	Yes	198	5454.7	362.99 (315.79 to 417.24)	100	2750.9	363.52 (298.82 to 442.23)
Premature Menopause (<45 years)	No	280	8175.4	342.49 (304.64 to 385.05)	127	4032.1	314.97 (264.69 to 374.81)
	Yes	36	1177.4	305.76 (220.56 to 423.89)	25	596.8	418.92 (283.07 to 619.97)
BMI - low (<18.5)	No	289	8876.1	325.59 (290.14 to 365.38)	141	4367.0	322.87 (273.75 to 380.82)
	Yes	11	187.0	588.16 (325.72 to 1062.04)	4	124.0	322.63 (121.09 to 859.62)
Rheumatoid Arthritis	No	289	8457.8	341.70 (304.49 to 383.45)	139	4135.1	336.15 (284.66 to 396.94)
	Yes	27	895.0	301.69 (206.90 to 439.93)	13	493.8	263.29 (152.88 to 453.43)

[Table continues on the next page]

For peer review only - http://bmjopen.bmj.com/site/about/guidelines.xhtml

Risk Factor cont.		Derivation No of incident cases	Derivation Total Person years	Derivation Crude Incidence Rate per 10000 person years (95% CI)	Validation No of incident cases	Validation Total Person years	Validation Crude Incidence Rate per 10000 person years (95% CI)
Secondary Osteoporosis	No	262	7846.5	333.9 (295.83 to 376.89)	122	3853.4	316.60 (265.12 to 378.07)
	Yes	54	1506.2	358.51 (274.58 to 468.10)	30	775.4	386.89 (270.51 to 553.34)
Previous Fracture	No	144	6832.0	210.77 (179.01 to 248.17)	63	3319.4	189.79 (148.26 to 242.95)
	Yes	172	2520.8	682.32 (587.61 to 792.31)	89	1309.4	679.69 (552.18 to 836.64)
Previous Fracture, detail	None	144	6832.0	210.77 (179.01 to 248.17)	63	3319.4	189.79 (148.26 to 242.95)
	1 fracture	105	1919.6	546.99 (451.76 to 662.29)	52	1049.8	495.36 (377.47 to 650.07)
	2-4 fractures	57	557.9	1021.62 (788.04 to 1324.45)	33	236.2	1397.28 (993.37 to 1965.44)
	5+ fractures	10	43.2	2311.27 (1243.59 to 4295.60)	4	23.5	1701.81 (638.72 to 4534.31)
**Total**		**316**	**9352.8**	**337.87 (302.60 to 377.25)**	**152**	**4628.8**	**328.38 (280.11 to 384.96)**

For peer review only

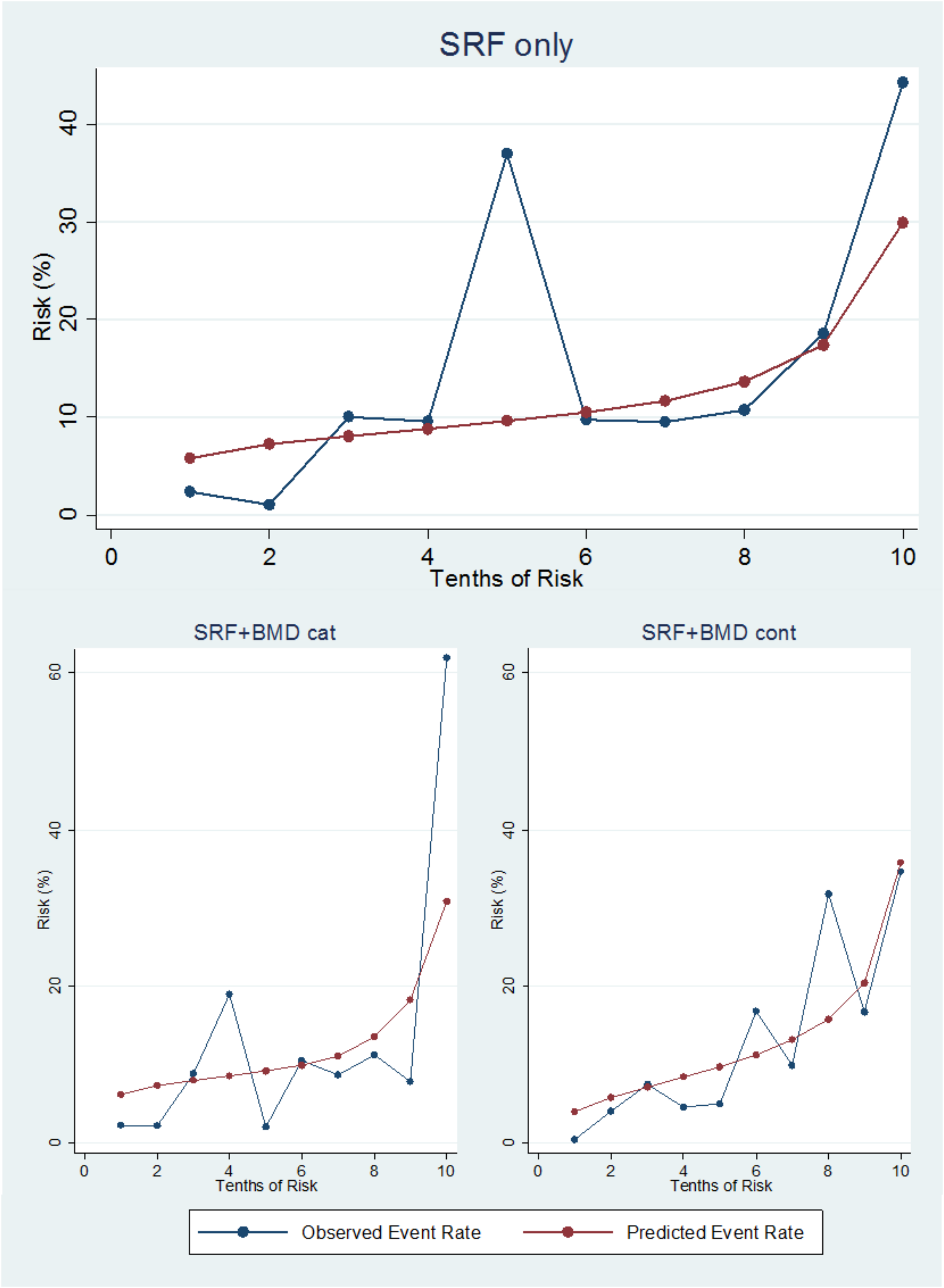

Supplementary Figure 1 Predicted and Observed risk by 10th of predicted risk for each risk prediction model in the derivation dataset.

TRIPOD Checklist: Prediction Model Development and Validation

Section/Topic	Item		Checklist Item	Page
**Title and abstract**				
Title	1	D;V	Identify the study as developing and/or validating a multivariable prediction model, the target population, and the outcome to be predicted.	1
Abstract	2	D;V	Provide a summary of objectives, study design, setting, participants, sample size, predictors, outcome, statistical analysis, results, and conclusions.	3
**Introduction**				
Background and objectives	3a	D;V	Explain the medical context (including whether diagnostic or prognostic) and rationale for developing or validating the multivariable prediction model, including references to existing models.	5
	3b	D;V	Specify the objectives, including whether the study describes the development or validation of the model or both.	5
**Methods**				
Source of data	4a	D;V	Describe the study design or source of data (e.g., randomized trial, cohort, or registry data), separately for the development and validation data sets, if applicable.	6
	4b	D;V	Specify the key study dates, including start of accrual; end of accrual; and, if applicable, end of follow-up.	6
Participants	5a	D;V	Specify key elements of the study setting (e.g., primary care, secondary care, general population) including number and location of centres.	6
	5b	D;V	Describe eligibility criteria for participants.	6
	5c	D;V	Give details of treatments received, if relevant.	-
Outcome	6a	D;V	Clearly define the outcome that is predicted by the prediction model, including how and when assessed.	6
	6b	D;V	Report any actions to blind assessment of the outcome to be predicted.	-
Predictors	7a	D;V	Clearly define all predictors used in developing or validating the multivariable prediction model, including how and when they were measured.	6/7
	7b	D;V	Report any actions to blind assessment of predictors for the outcome and other predictors.	-
Sample size	8	D;V	Explain how the study size was arrived at.	-
Missing data	9	D;V	Describe how missing data were handled (e.g., complete-case analysis, single imputation, multiple imputation) with details of any imputation method.	7
Statistical analysis methods	10a	D	Describe how predictors were handled in the analyses.	6/7
	10b	D	Specify type of model, all model-building procedures (including any predictor selection), and method for internal validation.	7
	10c	V	For validation, describe how the predictions were calculated.	6/7
	10d	D;V	Specify all measures used to assess model performance and, if relevant, to compare multiple models.	7/8
	10e	V	Describe any model updating (e.g., recalibration) arising from the validation, if done.	7
Risk groups	11	D;V	Provide details on how risk groups were created, if done.	8
Development vs. validation	12	V	For validation, identify any differences from the development data in setting, eligibility criteria, outcome, and predictors.	7
**Results**				
Participants	13a	D;V	Describe the flow of participants through the study, including the number of participants with and without the outcome and, if applicable, a summary of the follow-up time. A diagram may be helpful.	9
	13b	D;V	Describe the characteristics of the participants (basic demographics, clinical features, available predictors), including the number of participants with missing data for predictors and outcome.	9
	13c	V	For validation, show a comparison with the development data of the distribution of important variables (demographics, predictors and outcome).	10/11
Model development	14a	D	Specify the number of participants and outcome events in each analysis.	9
	14b	D	If done, report the unadjusted association between each candidate predictor and outcome.	11
Model specification	15a	D	Present the full prediction model to allow predictions for individuals (i.e., all regression coefficients, and model intercept or baseline survival at a given time point).	Suppl doc
	15b	D	Explain how to the use the prediction model.	Suppl doc
Model performance	16	D;V	Report performance measures (with CIs) for the prediction model.	13/14
Model-updating	17	V	If done, report the results from any model updating (i.e., model specification, model performance).	13/14
**Discussion**				
Limitations	18	D;V	Discuss any limitations of the study (such as nonrepresentative sample, few events per predictor, missing data).	15/16
Interpretation	19a	V	For validation, discuss the results with reference to performance in the development data, and any other validation data.	14
	19b	D;V	Give an overall interpretation of the results, considering objectives, limitations, results from similar studies, and other relevant evidence.	15/16
Implications	20	D;V	Discuss the potential clinical use of the model and implications for future research.	16
**Other information**				
Supplementary information	21	D;V	Provide information about the availability of supplementary resources, such as study protocol, Web calculator, and data sets.	Suppl doc
Funding	22	D;V	Give the source of funding and the role of the funders for the present study.	2

*Items relevant only to the development of a prediction model are denoted by D, items relating solely to a validation of a prediction model are denoted by V, and items relating to both are denoted D;V. We recommend using the TRIPOD Checklist in conjunction with the TRIPOD Explanation and Elaboration document.

For peer review only - http://bmjopen.bmj.com/site/about/guidelines.xhtml

BMJ Open

Does Bone Mineral Density Improve the Predictive Accuracy of Fracture Risk Assessment? A Prospective Cohort Study in Northern Denmark.

Journal:	*BMJ Open*
Manuscript ID	bmjopen-2017-018898.R3
Article Type:	Research
Date Submitted by the Author:	12-Mar-2018
Complete List of Authors:	Dhiman, Paula; University of Nottingham School of Medicine, Division of Primary Care<br>Andersen, Stig; Aalborg Universitetshospital, Geriatric Medicine, Department of Clinical Medicine<br>Vestergaard, Peter; Aalborg Universitetshospital, Department of Clinical Medicine and Endocrinology<br>Masud, Tahir ; University of Nottingham School of Medicine, Geriatric Medicine<br>Qureshi, Nadeem; University of Nottingham School of Medicine, Division of Primary Care
**Primary Subject Heading**:	General practice / Family practice
Secondary Subject Heading:	Research methods, Geriatric medicine, Public health
Keywords:	risk prediction, osteoporosis, fracture, predictive accuracy, bone mineral density

SCHOLARONE™
Manuscripts

For peer review only - http://bmjopen.bmj.com/site/about/guidelines.xhtml

Title

Does Bone Mineral Density Improve the Predictive Accuracy of Fracture Risk Assessment? A Prospective Cohort Study in Northern Denmark.

Authors list (names, affiliations, and position)

Paula Dhiman, Stig Andersen, Peter Vestergaard, Tahir Masud, Nadeem Qureshi

School of Medicine, Division of Primary Care, University of Nottingham, Nottingham, NG7 2RD, UK

Paula Dhiman (Research Fellow)

Geriatric Medicine, Department of Clinical Medicine, Aalborg University Hospital, Hobrovej 18-22 Aalborg University Hospital, 9000 Aalborg, Denmark

Stig Andersen (Clinical Professor)

Department of Clinical Medicine and Endocrinology, Aalborg University Hospital Mølleparkvej 4, DK-9000 Aalborg, Denmark

Peter Vestergaard (Clinical Professor)

Geriatric Medicine, South Block, B Floor, Queens Medical Centre Campus, University of Nottingham, Nottingham NG7 2UH, UK

Tahir Masud (Honorary Professor)

School of Medicine, Division of Primary Care, University of Nottingham, Nottingham, NG7 2RD, UK

Nadeem Qureshi (Clinical Professor)

Correspondence to: P Dhiman paula.dhiman@ndorms.ox.ac.uk

Copyright

The Corresponding Author has the right to grant on behalf of all authors and does grant on behalf of all authors, a worldwide license to the Publishers and its licensees in perpetuity, in all forms, formats and media (whether known now or created in the future), to i) publish, reproduce, distribute, display and store the Contribution, ii) translate the Contribution into other languages, create adaptations, reprints, include within collections and create summaries, extracts and/or, abstracts of the Contribution, iii) create any other derivative work(s) based on the Contribution, iv) to exploit all subsidiary rights in the Contribution, v) the inclusion of electronic links from the Contribution to third party material where-ever it may be located; and, vi) license any third party to do any or all of the above.

Exclusive License Statement

The Corresponding Author has the right to grant on behalf of all authors and does grant on behalf of all authors, an exclusive licence (or non exclusive for government employees) on a worldwide basis to the BMJ Publishing Group Ltd to permit this article (if accepted) to be published in BMJ editions and any other BMJPGL products and sublicenses such use and exploit all subsidiary rights, as set out in our licence.

For peer review only - http://bmjopen.bmj.com/site/about/guidelines.xhtml

Competing Interests

We have read and understood BMJ policy on declaration of interests and declare that we have no competing interests.

All authors have completed the ICMJE uniform disclosure form at www.icmje.org/coi_disclosure.pdf and declare: no support from any organisation for the submitted work; no financial relationships with any organisations that might have an interest in the submitted work in the previous three years; no other relationships or activities that could appear to have influenced the submitted work.

Transparency declaration

The lead author (Dr Dhiman) affirms that this manuscript is an honest, accurate, and transparent account of the study being reported; that no important aspects of the study have been omitted; and that any discrepancies from the study as planned (and, if relevant, registered) have been explained.

Ethical Approval

Ethics approval was given through the Region of North Jutland's from the Danish Data Protection Agency ("paraplyanmeldelse 2008-58-0028").

Funding

This study was funded through the National Institute for Health Research (NIHR), School for Primary Care Research (SPCR).

NIHR acknowledgement

This paper presents independent research funded by the National Institute for Health Research School for Primary Care Research (NIHR SPCR).

Disclaimer

The views expressed are those of the author(s) and not necessarily those of the NIHR, the NHS or the Department of Health.

Patient Involvement

Patients were not involved in the development of this research question.

Data Sharing Statement

Data sharing: Technical appendix and statistical code is available from the corresponding author at paula.dhiman@nottingham.ac.uk. Due to restrictions by the Danish Data Protection Agency, data can only be shared on an aggregated level and by special permission.

Contributorship

Contributors: PD wrote the statistical analysis plan, cleaned and analysed the data, and drafted and revised the paper. PV and SA provided the AURORA dataset for analysis and linked patients to the National Patient Registry of Denmark, they also reviewed and revised

For peer review only - http://bmjopen.bmj.com/site/about/guidelines.xhtml

the draft paper. NQ and TM provided clinical expertise, and reviewed and revised the draft paper.

For peer review only

For peer review only - http://bmjopen.bmj.com/site/about/guidelines.xhtml

Abstract:

Objective: To evaluate the added predictive accuracy of bone mineral density (BMD) to fracture risk assessment.

Design: Prospective cohort study using data between 01/01/2010 and 31/12/2012.

Setting: North Denmark Osteoporosis Clinic of referred patients presenting with at least one fracture risk factor to the referring doctor.

Participants: Patients aged 40-90 years; had BMD T-score recorded at the hip; and not taking osteoporotic preventing drugs for more than 1-year prior to baseline.

Main outcome measures: Incident diagnoses of osteoporotic fractures (hip, spine, forearm, humerus, and pelvis) were identified using the National Patient Registry of Denmark during 01/01/2012-01/01/2014. Cox regression was used to develop a fracture model based on predictors in the Fracture Risk Assessment Tool (FRAX®), with and without, binary and continuous BMD. Change in Harrell's C-Index and Reclassification tables were used to describe the added statistical value of BMD.

Results: Adjusting for predictors included in FRAX®, osteoporotic patients (T-score≤-2.5) had 75% higher hazard of a fracture compared to patients with higher BMD (HR:1.75 (95% CI:1.28 to 2.38)). Forty-percent lower hazard was found per unit increase in continuous BMD T-score (HR:0.60 (95% CI:0.52 to 0.69)).

Accuracy improved marginally, and Harrell's C-Index increased by 1.2% when adding continuous BMD (0.76 to 0.77). Reclassification tables showed continuous BMD shifted 529 patients into different risk categories; 292 of these were reclassified correctly (57%; 95% CI:55% to 64%). Adding binary BMD however no improvement: Harrell's C-Index decreased by 0.6%.

Conclusions: Continuous bone mineral density marginally improves fracture risk assessment. Importantly, this was only found when using continuous BMD measurement for osteoporosis. It is suggested that future focus should be on evaluation of this risk factor using routinely collected data, and on the development of more clinically relevant methodology to assess the added value of a new risk factor

For peer review only - http://bmjopen.bmj.com/site/about/guidelines.xhtml

Article Summary

Strengths and Limitations:

- Addresses a research question recommended by The National Institute for Health and Care Excellence to investigate the added value of bone mineral density to fracture risk prediction.
- Investigates bone mineral density in both the commonly used, binary, and continuous format.
- Presents changes in calibration, discrimination, and reclassification to describe the added value of bone mineral density.
- Uses robustly collected data from Northern Denmark, with 3.2% missing data.
- As data is from a North Danish population, with at least one fracture risk factor, this limits generalisability of the results.

For peer review only

For peer review only - http://bmjopen.bmj.com/site/about/guidelines.xhtml

Introduction

Osteoporosis causes over 8.9 million fractures worldwide, of which over 4.5 million occur in the USA and Europe, and account for 2.8 million disability adjusted life years (1). Further, 1.2 million disability adjusted life years are accounted for by hip fractures, which are projected to increase to 6 million by 2050 (2).

Given this burden, and treatment options for osteoporosis, identifying patients at risk of an osteoporotic fracture is high priority amongst health policymakers to reduce the risk of future fracture (3). Risk prediction tools have been developed to aid in the identification of patients at risk. For example, the Fracture Risk Assessment Tool (FRAX®) and QFracture® are commonly used to assess fracture risk in patients based on pre-defined risk factors.

Bone mineral density (BMD), a measurement used to aid diagnosis of osteoporosis, has also been identified as a fracture risk factor (4-7) . Unlike some other fracture risk factors, treatment options (e.g. bisphosphonate medication) are available that reduces the fracture risk markedly when treatment is initiated based on low BMD.

English National guidelines (The National Institute for Health and Care Excellence (NICE)) for fracture risk assessment recommend treatment of osteoporosis to prevent fractures but have not included BMD as a mandatory risk factor for fracture risk prediction tools to incorporate (8). This is partly due to the lack of robust evidence and limited generalisability of current research, which has particularly focused on evaluating BMD in postmenopausal women evaluating the added value of BMD to existing fracture risk factors (5-7).

The National Institute of Clinical Health and Excellence also recognise this gap in the evidence and have recommended research to assess the added value of BMD as a risk factor in fracture risk assessment (9).

The aim of this study is to assess the value of BMD measurement in addition to the standard fracture risk factors used in the FRAX® risk model using a robustly collected prospective cohort.

For peer review only

Methods

This paper has been written in accordance to the TRIPOD checklist.

Patient and Public Involvement

Patients and the public were not involved in the development of this research question and were not involved in the design of this study.

Study Design and Data Source

A prospective cohort study was conducted using patients from the Aalborg University Hospital Record for Osteoporosis Risk Assessment (AURORA) dataset; patients were followed up using the National Patient Registry of Denmark.

The AURORA dataset consists of patients attending the Osteoporosis Clinic at Aalborg University Hospital after a referral from their primary care physician. A referral was offered to patients with at least one risk factor for osteoporosis (low BMI, previous fracture, parental hip fracture, smoking status, alcohol consumption, glucocorticoid use, rheumatoid arthritis, and secondary osteoporosis) or if they were aged 80 years and above. Further detail of the data collection has been described elsewhere (10). The Danish National Patient Registry which collects inpatient and outpatient data from all Danish hospitals, was linked to the AURORA dataset through unique patient identifiers

Ethics approval was given through the Region of North Jutland's from the Danish Data Protection Agency ("paraplyanmeldelse 2008-58-0028").

Cohort selection

Data collection for AURORA began 1st January 2010 and was collected for 3 years (up to 31st December 2012). Patients were included if they were aged 40-90 years; had a BMD T-score at the hip; and were not taking any osteoporotic preventing drugs or any bone sparing drugs for more than one year prior to baseline.

Primary Outcome

The primary outcome measure was an incident osteoporotic fracture during follow up (01/01/2012 to 01/01/2014); defined as a diagnosis of a fracture at the hip, spine, forearm, humerus, and pelvis. Fractures at these sites resulting from traffic, work, and sports related accidents were excluded from the study. Relevant fractures were identified in the Danish National Patient Registry, using the International Statistical Classification of Diseases, 10th Version codes (ICD-10 codes), which was developed using recognised database methodology for each fracture (11).

Fracture risk factors

Fracture risk factors, used in the FRAX® risk prediction model, were extracted at baseline. They were: age; gender; height (m); weight (kg); previous fracture; parental history of hip fracture, current smoking status; current alcohol consumption; glucocorticoid use (currently exposed for 3+ months); rheumatoid arthritis; and secondary osteoporosis (includes type I diabetes; osteogenesis imperfecta in adults; untreated, long standing hyperthyroidism;

For peer review only - http://bmjopen.bmj.com/site/about/guidelines.xhtml

hypogonadism; premature menopause (<45 years); chronic malnutrition; malabsorption; and chronic liver disease).

Bone Mineral Density

DXA scans were performed by trained technicians using Hologic Discovery A (Bedford, MA, USA). A daily QC programme was in place and in vivo CV using repositioning of patients was <1%. Total hip BMD was used as region of interest. Bone mineral density was added to the fracture risk prediction model twice, firstly, as a continuously measured T-score value, and secondly, as a binary risk factor, dichotomised at/above T-score threshold for osteoporosis and below threshold, -2.5 in T-score (manufacturers' normal range using normal material from T Kelly et al (12)) based on World Health Organisation (WHO) classifications (13). Calculated T-scores were gender specific.

Statistical Analysis

A complete case analysis was performed on the data; 3.2% of data was missing. The AURORA dataset was split into two using recognised methodology (14); where a random number was assigned to patients and based on a cut off, two-thirds was used to derive the risk models, and the remaining third was used to validate them.

Model derivation

Three Cox proportional hazards models were developed for the primary outcome, using a complete case analysis on the derivation dataset:

Model 1. Standard fracture risk factors only (without BMD)
Model 2. Standard fracture risk factors (with binary BMD)
Model 3. Standard fracture risk factors (with continuous BMD)

Graphical methods were used (log-log plots) to assess the proportional hazards assumption, and risk factors violating this assumption were added to the model as a time varying covariate.

Recognised methodology used in research studies was used to build the 3 risk prediction models (15, 16); the Kaplan Meier method was used to obtain 4-year fracture risk estimates for patients. Further detail on the conversion of the Cox proportional hazards models to risk prediction models has been provided in Supplementary Table 1.

Validation of Models

Four-year fracture risk was calculated from each model and the predictive performance of each risk prediction model was assessed by measures describing calibration, discrimination, and reclassification. These metrics were assessed using the validation cohort.

Calibration measures how well the predicted risk agrees with observed risk in the data. It plots the mean predicted and observed risk of fracture for each decile of predicted risk. The observed risk of fracture was derived from the 4 year Kaplan-Meier estimate. Good calibration indicates the predicted risk is close to the observed risk of the outcome.

Discrimination measures how well the risk prediction model differentiates between patients who have or have not observed the event in the study. This was quantified by the area under

For peer review only - http://bmjopen.bmj.com/site/about/guidelines.xhtml

the receiver operating characteristic (ROC) curve (AUC), given by Harrell's C-Index with higher values indicating better discrimination.

Reclassification tables (17) measures movement between risk categories when adding a new risk factor. Threshold for treatment at 4 years was set at a fracture risk level of 8.5%; to be comparable to the treatment threshold of 20% at 10 years. This was presented by the total percent of patients reclassified (incorrectly and correctly), and also the Net Reclassification Index (NRI) (18, 19). The NRI gives the net calculation of the changes in the right direction and a higher NRI indicates a better reclassifying model.

All analyses were carried out using Stata (version 12) (20).

For peer review only

For peer review only - http://bmjopen.bmj.com/site/about/guidelines.xhtml

Results

Characteristics of the data

The AURORA collected data on 7,912 patients; 1,795 patients were excluded comprising, 440 not aged between 40-90 years at baseline; 156 not having a recorded T-score value for the total hip at baseline; and 1,199 patients were taking anti-osteoporotic drug therapy for more than one year prior to baseline.

The study sample consisted of 6,117 patients; predominantly female (79.6%), and patients with a mean age of 62.9 (SD: 10.9) years. Two-thirds of this sample (n=4,093) was used for the derivation dataset and one-third (n=2,094) was used for the validation dataset. Table 1 presents the baseline characteristics of the study by derivation and validation dataset, and shows little difference between the datasets.

Patients in the derivation dataset had a median follow up time of 2.30 years [1.57, 2.99], and observed 318 (7.8%) osteoporotic fractures during follow up. Of these, 316 fractures were eligible for the analysis (2 patients had a fractures on or prior to baseline and were excluded). Patients contributed 9352.8 person years of observation, giving a total incidence rate of 337.87 per 10,000 person years (95% CI:302.60 to 377.25).

Fractures during follow up were predominantly found in the forearm (27.0%) and hip (17.9%). Higher fracture incidence rates were found in patients classed as osteoporotic, based on their T-score at both the total hip (809.73 per 10,000 person years (95% CI:641.68 to 1021.78)) and spine (L1-L4) (553.59 per 10,000 person years (95% CI:462.55 to 662.55)) (Supplementary Table 2).

For peer review only

Table 1. Baseline characteristics of the derivation and validation datasets, including missing data.

Characteristic			Derivation (n=4,093)		Validation (n=2,024)	
			No	%	No	%
Gender	Female		3,266	79.8	1,602	79.2
	Male		827	20.2	422	20.8
Osteoporotic (Hip DXA)	No		3,683	90.0	1,820	89.9
	Yes		410	10.0	204	10.1
Osteoporotic Status (based on UK guidelines)	Normal		1,886	46.1	927	45.8
	Osteopenic		1,797	43.9	893	44.1
	Osteoporotic		410	10.0	204	10.1
Previous Fracture	No		2,935	71.7	1,423	70.3
	Yes		1,158	28.3	601	29.7
No of Previous Fractures	None		2,935	71.7	1,423	70.3
	1 fracture		862	21.1	467	23.1
	2-4 fractures		270	6.6	122	6.0
	5+ fractures		26	0.6	12	0.6
Parental History of Hip Fracture	No		2,755	67.3	1,359	67.1
	Yes		1,338	32.7	665	32.9
Current Smoking Status	other (non/ex)		3,182	77.7	1,529	75.5
	smoker		911	22.3	495	24.5
Alcohol Consumption	≤3 units per day		3,875	94.7	1,923	95.0
	>3 units per day		218	5.3	101	5.0
Glucocorticoid Use	No		3,577	87.4	1,741	86.0
	Yes		516	12.6	283	14.0
Rheumatoid Arthritis	No		3,686	90.1	1,801	88.0
	Yes		407	9.9	223	11.0
Other Bone Affecting Disease	No		2,382	58.2	1,139	56.3
	Yes		1,711	41.8	885	43.7
Secondary Osteoporosis	No		3,438	84.0	1,689	83.5
	Yes		655	16.0	335	16.6
	By disease					
	Type 1 diabetes	No	4,010	98.0	1,981	97.9
		Yes	83	2.0	43	2.1
	Osteogenesis	No	4,093	100	2,024	100
		Yes	0	0	0	0
	Hyperthyroidism	No	4,089	99.9	2,023	99.9
		Yes	4	0.1	1	0.1
	Malnutrition	No	4,090	99.9	2,023	99.9
		Yes	3	0.1	1	0.1
	Chronic Liver Disease	No	4,006	97.9	1,979	97.8
		Yes	87	2.1	45	2.2
	Menopause (Females only)**	No	853	26.1	405	25.3
		Yes	2,413	73.9	1,197	74.7

For peer review only - http://bmjopen.bmj.com/site/about/guidelines.xhtml

	Premature Menopause (<45years)***	No	1,904	78.9	941	78.6
		Yes	509	21.1	256	21.4
			**Mean**	**SD**	**Mean**	**SD**
Age (years)			62.9	10.9	63.0	11.0
Weight (kg)			72.1	15.5	72.2	15.9
	Missing		47	1.2	12	0.6
Height (m)			1.7	0.1	1.7	0.1
	Missing		131	3.2	61	3.0
BMI			26.4	5.0	26.4	5.1
	Missing		135	3.3	63	3.1
Hip DXA T-score			-1.1	1.1	-1.2	1.1

*out of patients with a fracture
**proportion out of respective number of females
***proportion out of respective number of females with menopause

Model development

The unadjusted analysis showed statistically significant association between BMD (continuous and binary) and osteoporotic fracture (HR=0.55; 95% CI: 0.50 to 0.61, p<0.001, HR=2.79; 95% CI: 2.11 to 3.67, p<0.001, respectively). Significant associations with fracture were also found with age (HR=1.03; 95% CI: 1.02 to 1.04, p<0.001), previous fracture (HR=3.38; 95% CI: 2.69 to 4.24, p<0.001), BMI (HR=0.97; 95% CI: 0.95 to 1.00, p=0.03), and gender (HR=0.73; 95% CI: 0.53 to 1.00, p=0.05). Further, a time-varying effect was found in patients with a previous fracture; hazard of a subsequent fracture was highest in the first year during follow up and decreased per year of follow up (p<0.001).

The adjusted analysis is presented in Table 2. Model 1 showed that of the standard risk factors, age and previous fracture were significantly associated with fracture; hazard of fracture increased by 2% per year increase in age (HR=1.024; 95% CI: 1.013 to 1.036); and increased almost 5 fold in patients with a previous fracture at time 0 years (HR=4.881; 95% CI: 3.336 to 7.078).

Hazard of fracture increased by 75% for patients classed as osteoporotic by their BMD score (Model 2, HR=1.745; 95% CI: 1.279 to 2.381). Hazard of fracture also decreased by 40% per SD improvement in BMD T-score (Model 3, HR=0.600; 95% CI: 0.524 to 0.686).

For peer review only - http://bmjopen.bmj.com/site/about/guidelines.xhtml

Table 2. Multivariable analysis for osteoporotic fracture in the derivation cohort. Data are adjusted hazard ratios and 95% confidence intervals.

Risk Factor		Adjusted Hazard Ratio (95% CI)		
		**Model 1: Standard Risk Factors only**	**Model 2: Standard Risk Factors + BMD (categorical)**	**Model 3: Standard Risk Factors + BMD (continuous)**
Age (years)		1.024 (1.013 to 1.036)	1.019 (1.007 to 1.031)	1.007 (0.995 to 1.019)
Gender	Female	Ref	Ref	Ref
	Male	0.754 (0.544 to 1.044)	0.796 (0.573 to 1.104)	0.851 (0.613 to 1.181)
BMI		0.978 (0.954 to 1.002)	0.989 (0.965 to 1.013)	1.027 (1.000 to 1.055)
Previous Fracture	No	Ref	Ref	Ref
	Yes (time = 0 years)	4.881 (3.336 to 7.078)	4.667 (3.214 to 6.778)	4.018 (2.763 to 5.842)
Parental History of Hip Fracture	No	Ref	Ref	Ref
	Yes	1.079 (0.834 to 1.397)	1.096 (0.847 to 1.419)	1.105 (0.854 to 1.430)
Current Smoker	No	Ref	Ref	Ref
	Yes	1.121 (0.852 to 1.475)	1.076 (0.817 to 1.417)	1.019 (0.774 to 1.342)
Alcohol Consumption (>3 units/day)	No	Ref	Ref	Ref
	Yes	1.414 (0.904 to 2.212)	1.440 (0.921 to 2.252)	1.459 (0.932 to 2.283)
Glucocorticoid Use	No	Ref	Ref	Ref
	Yes	1.080 (0.753 to 1.550)	1.052 (0.733 to 1.510)	1.038 (0.724 to 1.489)
Rheumatoid Arthritis	No	Ref	Ref	Ref
	Yes	1.098 (0.731 to 1.650)	1.089 (0.725 to 1.637)	1.116 (0.742 to 1.678)
Secondary Osteoporosis	No	Ref	Ref	Ref
	Yes	0.993 (0.729 to 1.354)	0.966 (0.708 to 1.317)	0.911 (0.667 to 1.243)
Osteoporotic	No	Ref	Ref	Ref
	Yes	-	1.745 (1.279 to 2.381)	-
Hip DXA T-score (SD)		-	-	0.600 (0.524 to 0.686)
Previous Fracture (TVC*)		0.635 (0.489 to 0.826)	0.639 (0.492 to 0.830)	0.644 (0.495 to 0.837)

*TVC = time varying covariate, value is interaction effect and 95% CI.

For peer review only - http://bmjopen.bmj.com/site/about/guidelines.xhtml

Model Validation

The 4-year predicted risk of fracture was calculated for all patients in the validation dataset; this was compared to the observed fracture outcome within the 4 year follow up.

Calibration and Discrimination

Calibration plots suggested some improvement when adding BMD measurement; particularly when including continuous BMD T-score measurement (Model 3; Supplementary Figure 1).

The largest change in discrimination was found when adding continuous BMD measurement to standard risk factors; Harrell's C-Index increased by 1.17% (Table 3). However, binary BMD measurement, as a measure for osteoporotic patients, was found to reduce Harrell's C-Index by -0.65%.

Table 3. Harrell's C-Index for Model 1, 2, 3, 4, and 5.

Model	Harrell's C-Index	Change in Harrell's C-Index (% change)*
**Model 1**: Standard fracture risk factors only (without BMD)	0.764 (0.718 to 0.810)	-
**Model 2**: Standard fracture risk factors only (with binary BMD)	0.759 (0.712 to 0.806)	-0.005 (-0.65%)
**Model 3**: Standard fracture risk factors only (with continuous BMD)	0.773 (0.732 to 0.814)	0.009 (1.17%)

*All change is measures against Model 1.

Reclassification

Reclassification tables indicated that adding continuous BMD measurement may improve classification of patients into their correct risk categories. This was not found when adding binary BMD. Table 4 presents the reclassification table for Model 1 (standard fracture risk factors only) and Model 3 (standard risk factors with continuous BMD), using the 8.5% pre-specified risk threshold.

For peer review only - http://bmjopen.bmj.com/site/about/guidelines.xhtml

Table 4. Risk Reclassification Table comparing Model 1 (standard fracture risk factors alone) to Model 3 (standard fracture risk factors with continuous BMD measurement), using a clinical 8.5% risk cut off.

			Model 3: SRF with continuous BMD			
			<8.5%	≥8.5%	Total	Total No.(%) Reclassified
**Model 1: SRF without BMD**	<8.5%	No	391	227	618	227 (36.7%)
		%	63.3%	36.7%		
		No. Events	5	9		
		No. Non events	386	218		
		Observed Event Rate	1.3%	4.0%		
	≥8.5%	No	302	1,040	1,342	302 (22.5%)
		%	22.5%	77.5%		
		No. Events	10	121		
		No. Non events	292	919		
		Observed Event Rate	3.3%	11.6%		
	Total		693	1,267	1,960	529 (27.0%)

Of the 1,960 patients in the validation dataset, 27% (n=529) were reclassified into a different risk category when including continuous BMD into fracture risk prediction. Two percent (9/529) were found to be reclassified correctly into a higher risk group and 55% (292/529) were reclassified correctly into a lower risk group; indicating 22% (292/1342) of patients at high risk in Model 1, not accounting for BMD measurement, were no longer at high risk. The net reclassification improvement when adding continuous BMD to standard risk factors, was 0.03, which resulted from increased specificity (non-event NRI = 4%) and decreased sensitivity (event NRI: -1%) from Model 1 (Table 5).

Table 5. Summary of Net Reclassification Index (NRI) and Integrated Discrimination Index (IDI) for all comparisons between developed fracture risk prediction models.

Comparison	Event NRI	Non-Event NRI	Overall NRI
Model 1 vs. Model 2	-3.45%	2.09%	-0.01
Model 1 vs. Model 3	-0.69%	4.08%	0.03

For peer review only

For peer review only - http://bmjopen.bmj.com/site/about/guidelines.xhtml

Discussion

Summary of Findings

Bone mineral density showed significant association with fracture risk with a 40% decrease for each SD rise in BMD. However, this resulted in small improvements in calibration, discrimination, and reclassification. The c-index estimate was slightly higher with continuous BMD but this increase is not conclusive given the width of the confidence intervals. Despite the limited improvement found of 1% in discrimination when adding continuous BMD, reclassification tables showed 57% of reclassified patients moving into their correct risk group through improved specificity. Importantly, no improvement was found when adding BMD in a binary format.

Our findings are consistent with and corroborate with current literature (7, 21, 22). Specifically, a study conducted in the Netherlands with 4 year follow up, investigating the added value of BMD for hip fractures risk, found modest improvement in predictability (21). Further, two more recent studies also indicated limited added value of BMD to fracture risk prediction (7, 22).

Strengths and Limitations

Answering Evidence gap

To our knowledge, this is the first study to investigate the added value of BMD in a binary and continuous format, to standard fracture risk factors. Further, it is based on a larger sample size than other studies investigating BMD in addition to FRAX (7, 21, 22). It helps inform the NICE research recommendation to assess the added value of BMD to routine fracture risk assessment in primary care (23). It further highlights that the more commonly used for treatment decision making, binary format of BMD resulted in a loss of predictability in fracture risk prediction; based on comparable measures for discrimination and reclassification

Robustness of Data

The prospective cohort was well populated with key standard risk factors recorded: BMI, smoking status and alcohol consumption, and personal and parental fracture history. Other than 3.2% of missing data for BMI, in 6,117 patients, complete data was collected for all risk factors (including BMD T-score recorded at the total hip). Further, the cohort was linked to a national robust electronic health records. This Danish National Patient Registry allowed for outcome fracture to be identified and also provided data on the mechanism for the fracture; this helped more accurately phenotype osteoporotic fractures.

Generalisability

The generalisability is affected in a few ways. Firstly, the findings are based on a Danish cohort. Secondly, AURORA data was collected from patients who presented to their doctor with at least one fracture risk factor and were referred to the osteoporosis clinic; this led to a biased study sample with a higher risk of a fracture and increased age. This could overestimate fracture risk amongst patients in a primary care setting.

Methodology

For peer review only - http://bmjopen.bmj.com/site/about/guidelines.xhtml

Due to the increased age of the sample, death becomes a competing risk. However, information on death was not collected and could not be retrieved. This limited the analysis of the data as competing risks could not be accounted for which may again lead to an overestimation of fracture risk (24). However, as an independent study primarily assessing the added value of BMD through deriving and validating the fracture risk prediction models, this bias would be present in both analyses to compare derived risk models with and without BMD measurement.

The FRAX risk algorithm has not yet been published, therefore FRAX estimates could not be directly calculated for the cohort. Instead, the FRAX risk model was recalibrated on the dataset with and without BMD added. Further, fracture outcomes in this study included pelvic fractures which are increasingly recognised as low trauma fragility fractures [(25)], and used BMD taken at the total hip instead of at the femur neck as it is the gold standard in Denmark (26).

Internal validation was performed to validate the derived risk prediction models. This may lead to over optimistic results of the performance of the risk models (14). To account for this limitation, a commonly practised method which randomly assigns patients to the derivation and validation datasets was used; further, a similar 1:2 ratio was also used to split the data (27-29).

The study had a 4 year follow up which is shorter than other recognised risk models. To account for this, we adapted the 20% clinical risk threshold for 10 year fracture estimates to 8.5% for 4 year fracture estimates, assuming that risk is constant over time (30, 31).

Traditional methodology assessing the added value to risk factors to existing risk prediction models are criticised to be insensitive to change, to lack interpretability (32-35). This was shown when finding a 1% change in Harrell's C-Index and overlapping confidence intervals between models, limiting the interpretability of results. Reclassification analysis was thus also used to provide more clinically interpretable results.

Clinical implications

The most notable clinical implication is the more routine use of BMD measurement for fracture risk assessment. Further, evidence suggests continuous BMD adds better predictability compared to the binary format.

Future Research

Further research is recommended to evaluate the added value of BMD to fracture risk prediction; in particular in addition to QFracture risk factors and using primary care routinely collected data. However, a brief interrogation into the Clinical Practice Research Datalink, a routinely collected UK primary care database, showed poor availability of BMD measurement in patient records, and thus, strong limitations to potential analyses. Less than 1% of patients had BMD recorded from a sample of 60,658 patients aged 40-90; not on any osteoporotic treatment; and with complete data for age, gender, BMI, smoking status, and alcohol consumption. Thus, prior to UK analysis, BMD recording in primary care databases needs to improve.

For peer review only - http://bmjopen.bmj.com/site/about/guidelines.xhtml

Methodologically, as well as assessing the added value of BMD to standard risk factors, we should also explore the option to replace existing fracture risk factors with the BMD measurement; this has rarely been explored in the literature but should be considered in future analyses. We also recommend research to investigate the added value of BMD in a potentially more natural, 3 group format of BMD (osteopenic, normal, osteoporotic).

In addition, further research is recommended to develop current methodology used to assess the added value of BMD to provide more clinically relevant results, such as cost implications; and to allow for better comparability between new risk factors with respect to their added value, thus improving decision making.

Conclusion

Continuous BMD marginally improves fracture risk assessment. Importantly, this was only found when using continuous BMD measurement for osteoporosis. It seems that prediction models for fragility fracture risk may be improved only marginally, using present risk factor assessment and evaluations. It is suggested that future focus should be on additional risk factors and on the development of more clinically relevant methodology to assess the added value of a new risk factor.

For peer review only

For peer review only - http://bmjopen.bmj.com/site/about/guidelines.xhtml

References

1. Kanis JA on behalf of the World Health Organization Scientific Group (2007). Assessment of osteoporosis at the primary health-care level. Technical Report. World Health Organization Collaborating Centre for Metabolic Bone Diseases, University of Sheffield, UK; 2007.
2. WHO scientific group. Prevention and management of osteoporosis: report of a WHO scientific group. WHO Technical Report Series. 2003.
3. Barry P, Aspray T, Briers K, Collins GS, Compston J, Dockery F, et al. Osteoporosis: assessing the risk of fragility fracture. 2012. Contract No.: CG146.
4. Kanis JA, Borgstrom F, Laet CD, Johansson H, Johnell O, Jonsson B, et al. Assessment of fracture risk. Osteoporosis International. 2005;16(6):581-9.
5. Report of a WHO Study Group. Assessment of fracture risk and its application to screening for postmenopausal osteoporosis. 1994.
6. McCloskey E, Johansson H, Oden A, Kanis JA. Fracture risk assessment. Clinical biochemistry. 2012;45(12):887-93.
7. Briot K, Paternotte S, Kolta S, Eastell R, Felsenberg D, Reid DM, et al. FRAX: Prediction of Major Osteoporotic Fractures in Women from the General Population: The OPUS Study. PLoS ONE. 2013;8(12):e83436.
8. National Institute for Health and Care Excellence. Osteoporosis: assessing the risk of fragility fracture. Short clinical guideline - evidence and recommedation. London: National Clinical Guidance Centre. 2012.
9. National Institute for Health and Care Excellence. Osteoporosis: assessing the risk of fragility fracture. NICE short clinical guideline CG146.; 2012.
10. Andersen S, Laurberg P. Age discrimination in osteoporosis screening-data from the Aalborg University Hospital Record for Osteoporosis Risk Assessment (AURORA). Maturitas. 2014;77(4):330-5.
11. Dave S, Petersen I. Creating medical and drug code lists to identify cases in primary care databases. Pharmacoepidemiol Drug Saf. 2009;18(8):704-7.
12. Kelly TL. Bone mineral density reference databases for American men and women. Journal of Bone and Mineral Research. 1990;5(Suppl 2).
13. Kanis JA, Melton LJr, Christianson C, Johnston CC, Khaltaev N. The diagnosis of osteoporosis. Journal of Bone and Mineral Research. 1994;9(8):1137-41.
14. Moons KGM, Kengne AP, Woodward M, Royston P, Vergouwe Y, Altman DG, et al. Risk prediction models: I. Development, internal validation, and assessing the incremental value of a new (bio)marker. Heart. 2012.
15. Hippisley-Cox J, Coupland C. Derivation and validation of updated QFracture algorithm to predict risk of osteoporotic fracture in primary care in the United Kingdom: prospective open cohort study. Bmj. 2012;344:e3427.
16. Hippisley-Cox J, Coupland C, Vinogradova Y, Robson J, Minhas R, Sheikh A, et al. Predicting cardiovascular risk in England and Wales: prospective derivation and validation of QRISK2. British Medical Journal. 2008;336:1475-82.
17. Cook NR. Use and misuse of the receiver operating characteristic curve in risk prediction. Circulation. 2007;115(7):928-35.
18. Cook NR. Comments on 'Evaluating the added predictive ability of a new marker: From area under the ROC curve to reclassification and beyond' by M. J. Pencina et al., Statistics in Medicine. Statistics in Medicine. 2008;27(2):191-5.
19. Pencina MJ, D'Agostino RB, Sr., Steyerberg EW. Extensions of net reclassification improvement calculations to measure usefulness of new biomarkers. Statistics in Medicine. 2011;30(1):11-21.

For peer review only - http://bmjopen.bmj.com/site/about/guidelines.xhtml

20. StataCorp. Stata Statistical Software: Release 12. College Station, TX: StataCorp LP; 2011.
21. Burger H, de Laet CE, Weel AE, Hofman A, Pols HA. Added value of bone mineral density in hip fracture risk scores. Bone. 1999;25(3):369-74.
22. Marques A, Lucas R, Simoes E, Verstappen SMM, Jacobs JWG, da Silva JAP. Do we need bone mineral density to estimate osteoporotic fracture risk? A 10-year prospective multicentre validation study. RMD Open. 2017;3(2):e000509.
23. Barry P, Aspray T, Briers K, Collins GS, Compston J, Dockery F, et al. Osteoporosis: assessing the risk of fragility fracture (NICE Clinical Guidance 146). National Institute for Health and Care Excellence; 2012. Contract No.: CG146.
24. Satagopan JM, Ben-Porat L, Berwick M, Robson M, Kutler D, Auerbach AD. A note on competing risks in survival data analysis. Br J Cancer. 2004;91(7):1229-35.
25. Soles G, Ferguson T. Fragility fractures of the pelvis. Curr Rev Musculoskelet Med. 2012;5:222-8.
26. Abrahamsen B, Stilgren LS, Hermann AP, Tofteng CL, Barenholdt O, Vestergaard P, et al. Discordance between changes in bone mineral density measured at different skeletal sites in perimenopausal women--implications for assessment of bone loss and response to therapy: The Danish Osteoporosis Prevention Study. J Bone Miner Res. 2001;16(7):1212-9.
27. Hippisley-Cox J, Coupland C, Vinogradova Y, Robson J, May M, Brindle P. Derivation and validation of QRISK, a new cardiovascular disease risk score for the United Kingdom: prospective open cohort study. British Medical Journal. 2007;335(7611):136-41.
28. Ridker PM, Buring JE, Rifai N, Cook NR. Development and validation of improved algorithms for the assessment of global cardiovascular risk in women: the Reynolds Risk Score.[Erratum appears in JAMA. 2007 Apr 4;297(13):1433]. Jama. 2007;297(6):611-9.
29. Hippisley-Cox J, Coupland C, Robson J, Brindle P. Derivation, validation, and evaluation of a new QRISK model to estimate lifetime risk of cardiovascular disease: cohort study using QResearch database. British Medical Journal. 2010;341.
30. National Osteoporosis Foundation. Clinician's Guide to Prevention and Treatment of Osteoporosis. Washington, DC; 2013.
31. Thomsen K. Odense Fallers and Osteoporosis Study: Assessment of osteoporosis among older women presenting with falls. Denmark: Odense University Hospital; 2014.
32. McGeechan K, Macaskill P, Irwig L, Liew G, Wong TY. Assessing New Biomarkers and Predictive Models for Use in Clinical Practice A Clinician's Guide. Archives of Internal Medicine. 2008;168(21):2304-10.
33. Steyerberg EW, Pencina MJ, Lingsma HF, Kattan MW, Vickers AJ, Van Calster B. Assessing the incremental value of diagnostic and prognostic markers: a review and illustration. European Journal of Clinical Investigation. 2012;42(2):216-28.
34. Janes H, Pepe MS, Gu W. Assessing the Value of Risk Predictions by Using Risk Stratification Tables. Annals of Internal Medicine. 2008;149(10):751-W162.
35. Cook NR. Use and misuse of the receiver operating characteristic curve in risk prediction - Response. Circulation. 2007;116(6):E134-E.

For peer review only

For peer review only - http://bmjopen.bmj.com/site/about/guidelines.xhtml

Supplementary Information

Beta coefficients from each Cox regression model were used to create each fracture risk prediction model.

Once all 5 models were finalised, their beta coefficients were used to create 5 risk prediction models and calculate risk of fracture for each patient, using the following general equation:

$$\widehat{risk} = 1 - S_0(t)^{exp(\sum_{i=1}^{p} \beta_i X_i - \sum_{i=1}^{p} \beta_i \bar{X}_i)}$$

Where $\mathbf{S_0}(\mathbf{t})$ is the baseline survival rate at follow up time, **t**(for this example, a follow up time of 10 years will be used); beta ($\boldsymbol{\beta}_\mathbf{i}$) are the regression coefficients for each included risk factor in the model (**i**); $\mathbf{X_i}$ is the observed data value for each risk factor; $\bar{\mathbf{X}}_\mathbf{i}$ is the corresponding mean for each risk factor; and **p** is the total number of risk factors included in the model. Table A1 shows the formula for each risk prediction model explicitly.

For peer review only

Supplementary Table 1. Risk equations to calculate 4 year risk based on patient characteristics for each developed risk model.

**General Risk Equation to calculate 4 year risk**	$1-0.89^{exp(\sum_{i=1}^{p}\beta_i X_i - \sum_{i=1}^{p}\beta_i \bar{X}_i)}$
**Risk Model**	**Equation**
Model 1	where $\sum_{i=1}^{p}\beta_i X_i =$<br>0.0237745*age+-0.2826461*gender+-0.0225011*BMI+1.585278*previous fracture+<br>0.0762559*parental hip fracture+0.1138883*smoking status+0.0773898*glucocorticoid use+<br>0.3465287*alcohol consumption+0.0936966*rheumatoid arthritis+<br>-0.0069432*secondary osteoporosis+-0.4535108*(previous fracture*time)<br>where $\sum_{i=1}^{p}\beta_i \bar{X}_i =$<br>0.0237745*mean age+-0.2826461*mean gender+-0.0225011*mean BMI+<br>1.585278*mean previous fracture+0.0762559*mean parental hip fracture+<br>0.1138883*mean smoking status+0.0773898*mean glucocorticoid use+<br>0.3465287*mean alcohol consumption+0.0936966*mean rheumatoid arthritis+<br>-0.0069432*mean secondary osteoporosis+-0.4535108*mean (previous fracture*time)
Model 2	where $\sum_{i=1}^{p}\beta_i X_i =$<br>0.0186827*age+-0.228784*gender+-0.0113651*BMI+1.540559*previous fracture+<br>0.092011*parental hip fracture+0.0732564*smoking status+0.0508706*glucocorticoid use+<br>0.3649544*alcohol consumption+0.0854353*rheumatoid arthritis+ -0.0346885*secondary osteoporosis+0.5568944*osteoporosis+-0.4481145*(previous fracture*time)<br>where $\sum_{i=1}^{p}\beta_i \bar{X}_i =$<br>0.0186827*mean age+-0.228784*mean gender+-0.0113651*mean BMI+<br>1.540559*mean previous fracture+0.092011*mean parental hip fracture+<br>0.0732564*mean smoking status+0.0508706*mean glucocorticoid use+<br>0.3649544*mean alcohol consumption+0.0854353*mean rheumatoid arthritis+<br>-0.0346885*mean secondary osteoporosis+0.5568944*mean osteoporosis+<br>-0.4481145*mean (previous fracture*time)

[Table continues on the next page]

For peer review only

Risk Model cont.	Equation
Model 3	where $\sum_{i=1}^{p} \beta_i X_i =$ 0.0071931*age+-0.1615582*gender+0.0268478*BMI+1.39069*previous fracture+ 0.1000272*parental hip fracture+0.0192416*smoking status+0.0374944*glucocorticoid use+ 0.3774416*alcohol consumption+0.1097646*rheumatoid arthritis+ -0.0932063*secondary osteoporosis+-0.5110986*t-score+-0.4404955*(previous fracture*time)<br>where $\sum_{i=1}^{p} \beta_i \bar{X}_i =$ 0.0071931*mean age+-0.1615582*mean gender+0.0268478*mean BMI+ 1.39069*mean previous fracture+0.1000272*mean parental hip fracture+ 0.0192416*mean smoking status+0.0374944*mean glucocorticoid use+ 0.3774416*mean alcohol consumption+0.1097646*mean rheumatoid arthritis+ -0.0932063*mean secondary osteoporosis+-0.5110986*mean t-score+ -0.4404955*mean (previous fracture*time)

For peer review only

Supplementary Table 2. Crude fracture incidence rates for the derivation and validation datasets.

Risk Factor		Derivation			Validation		
		No of incident cases	Total Person years	Crude Incidence Rate per 10000 person years (95% CI)	No of incident cases	Total Person years	Crude Incidence Rate per 10000 person years (95% CI)
Age Category	40-49	17	1169.9	145.31 (90.33 to 233.74)	12	557.8	215.15 (122.19 to 378.84)
	50-59	70	2534.7	276.17 (218.49 to 349.07)	33	1311.0	251.71 (178.95 to 354.06)
	60-69	93	3062.9	303.63 (247.79 to 372.06)	42	1453.9	288.87 (213.48 to 390.88)
	70-79	83	1906.0	435.46 (351.17 to 539.98)	49	958.7	511.11 (386.29 to 676.26)
	80-89	52	652.7	796.75 (607.13 to 1045.59)	16	347.4	460.56 (282.15 to 751.77)
	90-99	1	26.6	376.51 (53.04 to 2672.85)	-	-	-
Osteoporotic - Hip	No	245	8475.9	289.05 (255.03 to 327.61)	123	4165.1	295.31 (247.48 to 352.40)
	Yes	71	876.8	809.73 (641.68 to 1021.78)	29	463.8	625.28 (434.52 to 899.78)
Osteoporotic - Spine	No	191	7025.8	271.86 (235.91 to 313.28)	111	3475.6	319.37 (265.16 to 384.67)
	Yes	119	2149.6	553.59 (462.55 to 662.55)	39	1089.1	358.08 (261.63 to 490.10)
Gender	Female	266	7417.5	358.61 (318.01 to 404.40)	129	3679.8	350.56 (295.00 to 416.59)
	Male	50	1935.3	258.36 (195.82 to 340.88)	23	949.0	242.36 (161.05 to 364.71)
Parental History Hip Fracture	No	220	6281.5	350.24 (306.88 to 399.71)	118	3108.7	379.58 (316.92 to 454.64)
	Yes	96	3071.3	312.57 (255.90 to 381.79)	34	1520.2	223.66 (159.81 to 313.02)

[Table continues on the next page]

For peer review only

Risk Factor cont.		Derivation: No of incident cases	Derivation: Total Person years	Derivation: Crude Incidence Rate per 10000 person years (95% CI)	Validation: No of incident cases	Validation: Total Person years	Validation: Crude Incidence Rate per 10000 person years (95% CI)
Current Smoker	No	240	7279.1	329.71 (290.53 to 374.18)	103	3513.1	293.19 (241.70 to 355.65)
	Yes	76	2073.7	366.5 (292.71 to 458.90)	49	1115.8	439.16 (331.91 to 581.07)
Alcohol Consumption more than 3 units per day	No	293	8875.9	330.11 (294.39 to 370.15)	140	4399.7	318.2 (269.63 to 375.53)
	Yes	23	476.9	482.33 (320.52 to 725.83)	12	229.2	523.66 (297.39 to 922.09)
Glucocorticoid Use (3 months)	No	279	8184.5	340.89 (303.15 to 383.33)	132	3993.1	330.57 (278.73 to 392.06)
	Yes	37	1168.3	316.7 (229.47 to 437.11)	20	635.8	314.57 (202.95 to 487.59)
Menopause	No	68	1962.8	346.44 (273.15 to 439.39)	29	928.9	312.19 (216.94 to 449.24)
	Yes	198	5454.7	362.99 (315.79 to 417.24)	100	2750.9	363.52 (298.82 to 442.23)
Premature Menopause (<45 years)	No	280	8175.4	342.49 (304.64 to 385.05)	127	4032.1	314.97 (264.69 to 374.81)
	Yes	36	1177.4	305.76 (220.56 to 423.89)	25	596.8	418.92 (283.07 to 619.97)
BMI - low (<18.5)	No	289	8876.1	325.59 (290.14 to 365.38)	141	4367.0	322.87 (273.75 to 380.82)
	Yes	11	187.0	588.16 (325.72 to 1062.04)	4	124.0	322.63 (121.09 to 859.62)
Rheumatoid Arthritis	No	289	8457.8	341.70 (304.49 to 383.45)	139	4135.1	336.15 (284.66 to 396.94)
	Yes	27	895.0	301.69 (206.90 to 439.93)	13	493.8	263.29 (152.88 to 453.43)

[Table continues on the next page]

For peer review only

Risk Factor cont.		Derivation: No of incident cases	Derivation: Total Person years	Derivation: Crude Incidence Rate per 10000 person years (95% CI)	Validation: No of incident cases	Validation: Total Person years	Validation: Crude Incidence Rate per 10000 person years (95% CI)
Secondary Osteoporosis	No	262	7846.5	333.9 (295.83 to 376.89)	122	3853.4	316.60 (265.12 to 378.07)
	Yes	54	1506.2	358.51 (274.58 to 468.10)	30	775.4	386.89 (270.51 to 553.34)
Previous Fracture	No	144	6832.0	210.77 (179.01 to 248.17)	63	3319.4	189.79 (148.26 to 242.95)
	Yes	172	2520.8	682.32 (587.61 to 792.31)	89	1309.4	679.69 (552.18 to 836.64)
Previous Fracture, detail	None	144	6832.0	210.77 (179.01 to 248.17)	63	3319.4	189.79 (148.26 to 242.95)
	1 fracture	105	1919.6	546.99 (451.76 to 662.29)	52	1049.8	495.36 (377.47 to 650.07)
	2-4 fractures	57	557.9	1021.62 (788.04 to 1324.45)	33	236.2	1397.28 (993.37 to 1965.44)
	5+ fractures	10	43.2	2311.27 (1243.59 to 4295.60)	4	23.5	1701.81 (638.72 to 4534.31)
**Total**		**316**	**9352.8**	**337.87 (302.60 to 377.25)**	**152**	**4628.8**	**328.38 (280.11 to 384.96)**

For peer review only

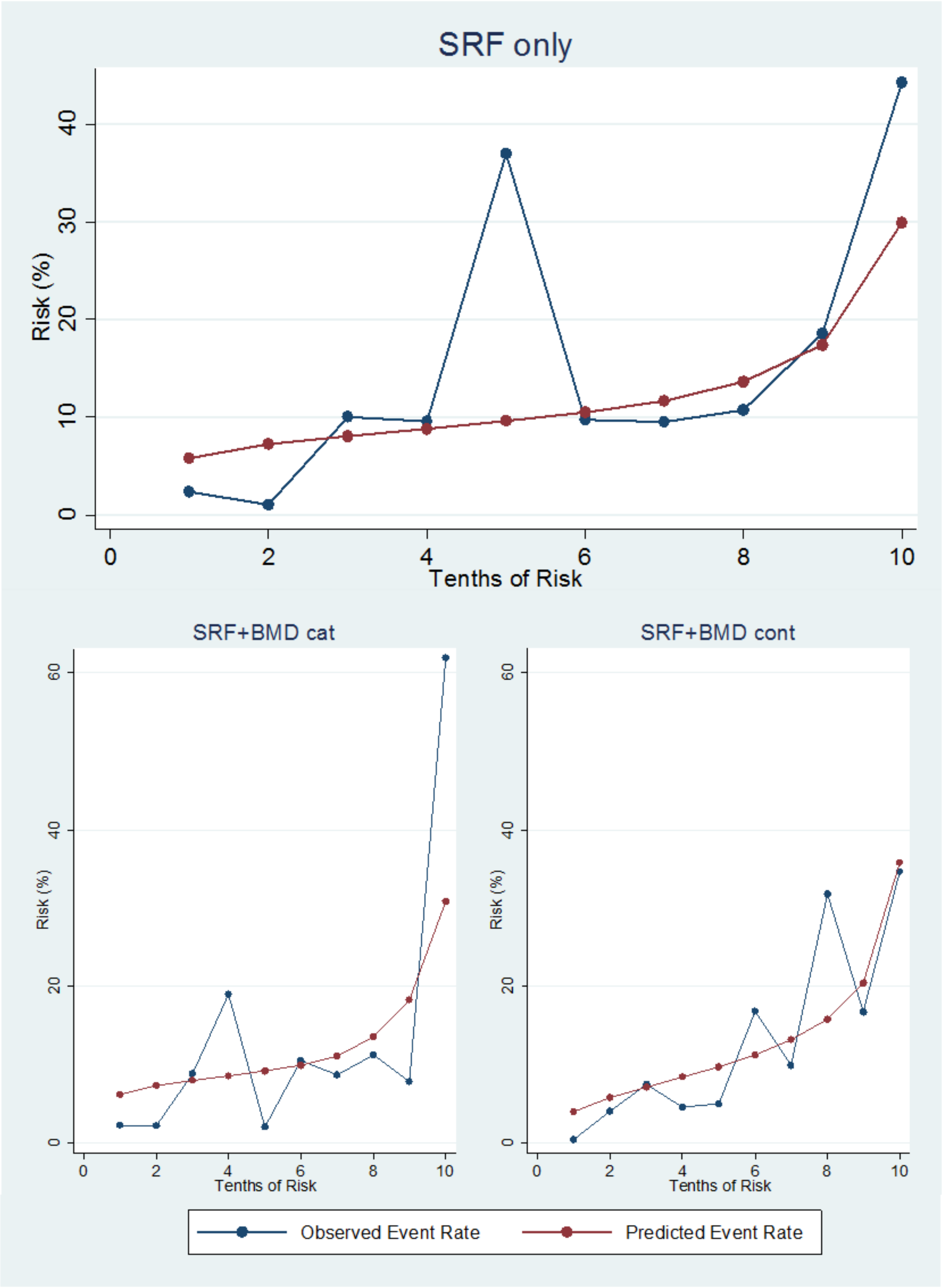

Supplementary Figure 1 Predicted and Observed risk by 10th of predicted risk for each risk prediction model in the derivation dataset.

TRIPOD Checklist: Prediction Model Development and Validation

Section/Topic	Item		Checklist Item	Page
**Title and abstract**				
Title	1	D;V	Identify the study as developing and/or validating a multivariable prediction model, the target population, and the outcome to be predicted.	1
Abstract	2	D;V	Provide a summary of objectives, study design, setting, participants, sample size, predictors, outcome, statistical analysis, results, and conclusions.	3
**Introduction**				
Background and objectives	3a	D;V	Explain the medical context (including whether diagnostic or prognostic) and rationale for developing or validating the multivariable prediction model, including references to existing models.	5
	3b	D;V	Specify the objectives, including whether the study describes the development or validation of the model or both.	5
**Methods**				
Source of data	4a	D;V	Describe the study design or source of data (e.g., randomized trial, cohort, or registry data), separately for the development and validation data sets, if applicable.	6
	4b	D;V	Specify the key study dates, including start of accrual; end of accrual; and, if applicable, end of follow-up.	6
Participants	5a	D;V	Specify key elements of the study setting (e.g., primary care, secondary care, general population) including number and location of centres.	6
	5b	D;V	Describe eligibility criteria for participants.	6
	5c	D;V	Give details of treatments received, if relevant.	-
Outcome	6a	D;V	Clearly define the outcome that is predicted by the prediction model, including how and when assessed.	6
	6b	D;V	Report any actions to blind assessment of the outcome to be predicted.	-
Predictors	7a	D;V	Clearly define all predictors used in developing or validating the multivariable prediction model, including how and when they were measured.	6/7
	7b	D;V	Report any actions to blind assessment of predictors for the outcome and other predictors.	-
Sample size	8	D;V	Explain how the study size was arrived at.	-
Missing data	9	D;V	Describe how missing data were handled (e.g., complete-case analysis, single imputation, multiple imputation) with details of any imputation method.	7
Statistical analysis methods	10a	D	Describe how predictors were handled in the analyses.	6/7
	10b	D	Specify type of model, all model-building procedures (including any predictor selection), and method for internal validation.	7
	10c	V	For validation, describe how the predictions were calculated.	6/7
	10d	D;V	Specify all measures used to assess model performance and, if relevant, to compare multiple models.	7/8
	10e	V	Describe any model updating (e.g., recalibration) arising from the validation, if done.	7
Risk groups	11	D;V	Provide details on how risk groups were created, if done.	8
Development vs. validation	12	V	For validation, identify any differences from the development data in setting, eligibility criteria, outcome, and predictors.	7
**Results**				
Participants	13a	D;V	Describe the flow of participants through the study, including the number of participants with and without the outcome and, if applicable, a summary of the follow-up time. A diagram may be helpful.	9
	13b	D;V	Describe the characteristics of the participants (basic demographics, clinical features, available predictors), including the number of participants with missing data for predictors and outcome.	9
	13c	V	For validation, show a comparison with the development data of the distribution of important variables (demographics, predictors and outcome).	10/11
Model development	14a	D	Specify the number of participants and outcome events in each analysis.	9
	14b	D	If done, report the unadjusted association between each candidate predictor and outcome.	11
Model specification	15a	D	Present the full prediction model to allow predictions for individuals (i.e., all regression coefficients, and model intercept or baseline survival at a given time point).	Suppl doc
	15b	D	Explain how to the use the prediction model.	Suppl doc
Model performance	16	D;V	Report performance measures (with CIs) for the prediction model.	13/14
Model-updating	17	V	If done, report the results from any model updating (i.e., model specification, model performance).	13/14
**Discussion**				
Limitations	18	D;V	Discuss any limitations of the study (such as nonrepresentative sample, few events per predictor, missing data).	15/16
Interpretation	19a	V	For validation, discuss the results with reference to performance in the development data, and any other validation data.	14
	19b	D;V	Give an overall interpretation of the results, considering objectives, limitations, results from similar studies, and other relevant evidence.	15/16
Implications	20	D;V	Discuss the potential clinical use of the model and implications for future research.	16
**Other information**				
Supplementary information	21	D;V	Provide information about the availability of supplementary resources, such as study protocol, Web calculator, and data sets.	Suppl doc
Funding	22	D;V	Give the source of funding and the role of the funders for the present study.	2

*Items relevant only to the development of a prediction model are denoted by D, items relating solely to a validation of a prediction model are denoted by V, and items relating to both are denoted D;V. We recommend using the TRIPOD Checklist in conjunction with the TRIPOD Explanation and Elaboration document.

For peer review only - http://bmjopen.bmj.com/site/about/guidelines.xhtml
